# E3 Ligase Ligands in Successful PROTACs: An Overview of Syntheses and Linker Attachment Points

**DOI:** 10.3389/fchem.2021.707317

**Published:** 2021-07-05

**Authors:** Aleša Bricelj, Christian Steinebach, Robert Kuchta, Michael Gütschow, Izidor Sosič

**Affiliations:** ^1^ Faculty of Pharmacy, University of Ljubljana, Ljubljana, Slovenia; ^2^ Pharmaceutical Institute, University of Bonn, Bonn, Germany

**Keywords:** bifunctional molecules, cereblon, drug synthesis, E3 ligase ligands, linker attachment, PROTACs, von Hippel–Lindau

## Abstract

Proteolysis-targeting chimeras (PROTACs) have received tremendous attention as a new and exciting class of therapeutic agents that promise to significantly impact drug discovery. These bifunctional molecules consist of a target binding unit, a linker, and an E3 ligase binding moiety. The chemically-induced formation of ternary complexes leads to ubiquitination and proteasomal degradation of target proteins. Among the plethora of E3 ligases, only a few have been utilized for the novel PROTAC technology. However, extensive knowledge on the preparation of E3 ligands and their utilization for PROTACs has already been acquired. This review provides an in-depth analysis of synthetic entries to functionalized ligands for the most relevant E3 ligase ligands, i.e. CRBN, VHL, IAP, and MDM2. Less commonly used E3 ligase and their ligands are also presented. We compare different preparative routes to E3 ligands with respect to feasibility and productivity. A particular focus was set on the chemistry of the linker attachment by discussing the synthetic opportunities to connect the E3 ligand at an appropriate exit vector with a linker to assemble the final PROTAC. This comprehensive review includes many facets involved in the synthesis of such complex molecules and is expected to serve as a compendium to support future synthetic attempts towards PROTACs.

## Introduction

The ubiquitin-proteasome system (UPS) plays a cardinal role in maintaining intracellular protein homeostasis by eliminating misfolded, damaged, and worn-out proteins (Amm et al., 2014). This process consists of a cascade of distinct steps, starting with ubiquitin activation by enzyme E1. Ubiquitin is then passed to the E2 or ubiquitin-conjugating enzyme by *trans*-thioesterification. Subsequently, E3 ubiquitin ligase promotes the transfer of ubiquitin onto a lysine of the substrate protein. Ubiquitin’s own internal lysine residues allow binding of additional ubiquitins, resulting in polyubiquitin tags, which serve as a signal for protein degradation via the 26S proteasome (Kleiger and Mayor, 2014).

**FIGURE 1:**
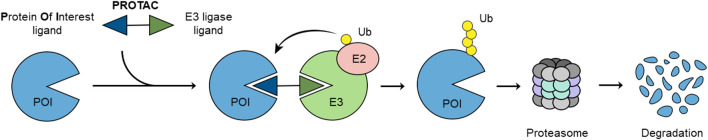
PROTAC-induced degradation of target proteins.

Hijacking the UPS and utilizing its functions to degrade the selected protein of interest (POI) has been made possible by proteolysis-targeting chimeras (PROTACs) (Burslem and Crews, 2020) (Figure 1). These hetero-bifunctional molecules are composed of a POI ligand connected to an E3 ubiquitin ligase ligand by a linker (Figure 1) (Pettersson and Crews, 2019). A functional PROTAC instigates the formation of a ternary complex POI-PROTAC-E3 ligase, which results in the ubiquitination of the POI, followed by proteasomal degradation (Scheepstra et al., 2019). This new modality began accumulating recognition and significance in medicinal chemistry since 2001 when the first proof-of-concept experiments were published (Sakamoto et al., 2001; Burslem and Crews, 2020).

## E3 Ligases

The human genome includes two members of the E1 enzyme family, roughly 40 E2s, and more than 600 E3 ubiquitin ligases (Kleiger and Mayor, 2014). E3 ligases represent a crucial element in protein ubiquitination due to their role in substrate selection and modulation of the cascade's efficiency (Buetow and Huang, 2016; Zheng and Shabek, 2017). They are categorized into three classes, based on their mechanism of ubiquitin transfer. The first and the most abundant class includes approximately 600 RING (Really Interesting New Gene) E3 ligases, which catalyze the direct transfer of ubiquitin from E2 to a substrate. In contrast, the less represented E3 classes HECT (Homologous to E6AP C-terminus) and RBR (RING-between-RING) form a thioester intermediate with ubiquitin *via* a catalytic cysteine before the transfer to the substrate protein (Buetow and Huang, 2016). Although our understanding of substrate recognition and regulation of ubiquitination is incomplete, the genome’s selection of roughly 600 E3 ligases is capable of ubiquitinating a much larger number of protein substrates in a controlled manner with ample specificity (Fisher and Phillips, 2018).

Despite the vast selection of known E3 ligases, only a handful have been successfully utilized in PROTAC compounds (Burslem and Crews, 2020). Following the first utilization of a poorly permeable phosphopeptide moiety to hijack Skp1–Cullin–F box complex (SCF^β-TRCP^) to degrade methionine aminopeptidase-2 (Sakamoto et al., 2001), and targeting the von Hippel-Lindau (VHL) tumor suppressor protein with a seven amino acid long sequence ALAPYIP (Schneekloth, et al., 2004), the field has evolved tremendously, resulting in numerous small-molecule E3 ligands, that allow for the development of cell-permeable and biologically active PROTACs (Sun X. et al., 2019). The first of its kind was a PROTAC targeting the androgen receptor, using nutlin (Figure 8) to recruit the mouse double minute 2 homologue (MDM2) E3 ligase (Schneekloth et al., 2008). Following that, the number of successfully degraded targets using various E3 ligases, such as cellular inhibitor of apoptosis (cIAP) (Itoh et al., 2010), VHL, and cereblon (CRBN) (Sun X. et al., 2019), steeply increased. More recently, additional E3s were explored and used successfully in degraders, *i.e*., RING-type zinc-finger protein 114 (RNF114) (Spradlin et al., 2019), damage-specific DNA binding protein 1 (DDB1)-CUL4 associated factor 16 (DCAF16) (Zhang et al., 2019), and Kelch-like ECH-associated protein 1 (KEAP1) (Tong et al., 2020a). However, the majority of recently reported PROTACs still utilize either VHL or CRBN as E3 ligases (Burslem and Crews, 2020); a fact that is corroborated by a high number of different synthetic approaches to obtain these PROTAC building blocks.

Various aspects of degraders have been extensively reviewed in the scientific literature in recent years (Toure and Crews, 2016; Lai and Crews, 2017; An and Fu, 2018; Sun X. et al., 2019; Pettersson and Crews, 2019; Schapira et al., 2019). However, a thorough overview of synthetic efforts leading to the most commonly used ligands for E3 ligases has not been done. Therefore, in this review, we focus on E3 ligase ligands utilized in successful PROTACs. More precisely, we overview the synthetic routes to obtain the E3 ligands and illustrate the possible linker attachment points and types of bonds used to connect the ligands with linkers. The preparation of specific building blocks was reported, as expected, in many publications. However, if no yields were reported or the authors only referred to the original or previously described work, these publications were not referenced in this paper. In addition, for the most commonly used ligases, a statistical overview of the prevalence of E3 ligase ligands and linker attachment options utilized in PROTACs is provided, along with highlighting the contributions of these building blocks to the physicochemical properties of final PROTAC molecules. This review provides the reader with a concise picture of the current state and enables all newcomers to the field a quick go-to-guide in terms of synthetic access to PROTAC building blocks. We hope that this thorough overview will aid in future successful contributions in the protein degradation field.

### Cereblon

CRBN is a 442-amino acid protein that forms a Cullin-4-RING E3 ubiquitin ligase (CRL4) complex and interacts with the adaptor protein damaged DNA–binding protein 1 (DDB1) (Ito et al., 2011; Chamberlain et al., 2014). Within the CRL4 complex, CRBN acts as a substrate-specificity receptor (Chamberlain et al., 2014). Known ligands for CRBN include thalidomide and other derived immunomodulatory imide drugs (IMiDs). Upon binding of IMiDs to CRBN, the E3 ubiquitin ligase activity of CRBN is re-modulated (Zhu et al., 2011; Chamberlain et al., 2014; Krönke et al., 2014; Krönke et al., 2015). As a result, an increase in the recruitment of the transcription factors Ikaros (IKZF1) and Aiolos (IKZF3) occurs, which leads to their subsequent ubiquitination and proteasomal degradation. This interaction and its outcome are responsible for the antiproliferative effects of thalidomide, lenalidomide, and pomalidomide in multiple myeloma (Chamberlain et al., 2014; Krönke et al., 2014; Krönke et al., 2015).

**FIGURE 2:**
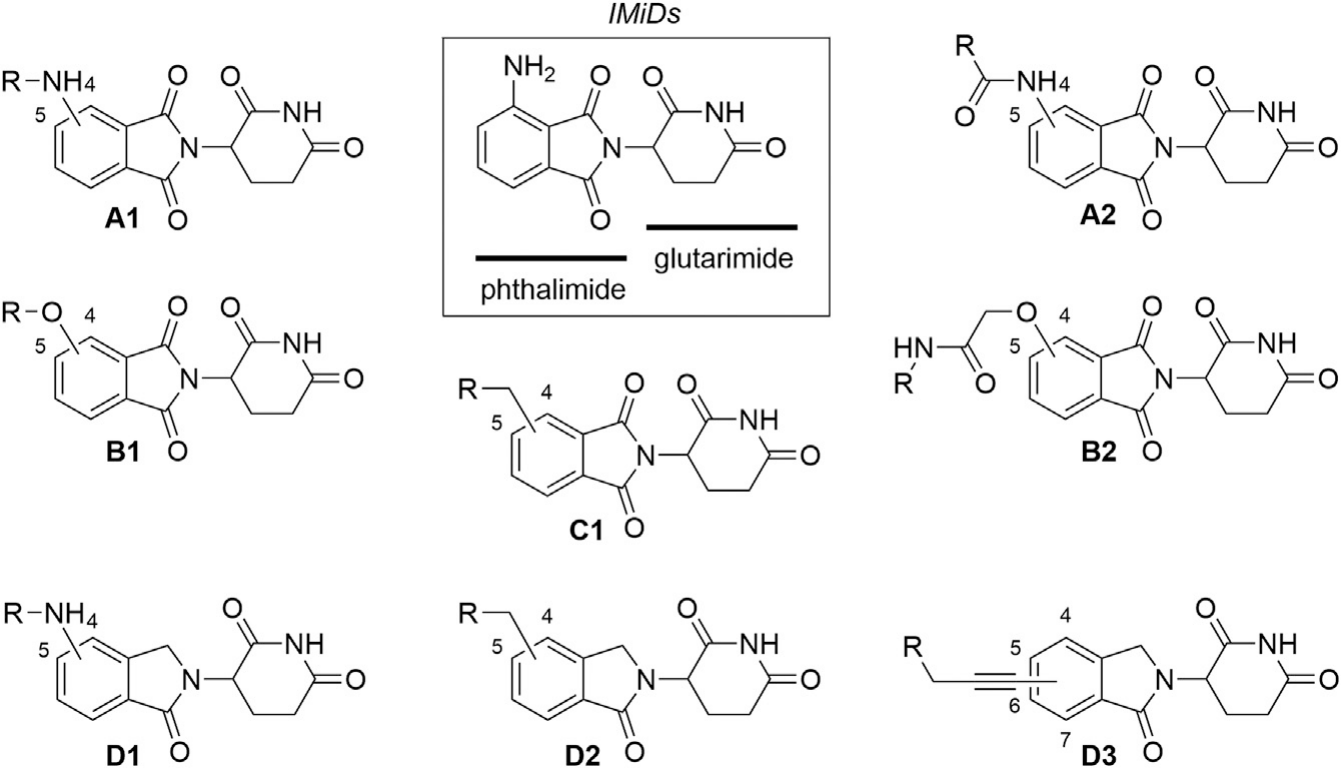
Commonly utilized thalidomide-derived CRBN ligands and possible linker attachment styles. **(A1**–**A2)** pomalidomide derivatives; **(B1**–**B2)** 4-hydroxythalidomide derivatives; **(C1)** alkyl type attachment to thalidomide; **(D1**–**D3)** lenalidomide derivatives.

To date, CRBN has been successfully utilized as the E3 ligase in PROTAC targeting more than 30 different proteins, ranging from those involved in various cancers (Sun X. et al., 2019) and immune disorders (Bassi et al., 2018), to neurodegenerative disease-associated protein Tau (Silva et al., 2019), and even hepatitis C virus protein NS3 (de Wispelaere et al., 2019). The collection of CRBN ligands with different linker attachment options are presented in Figure 2. The majority of CRBN-targeting PROTACs employ derivatives of pomalidomide (Figure 2, **A1**, **A2**), 4-hydroxythalidomide (Figure 2, **B1**, **B2**), alkyl-connected thalidomide derivatives (Figure 2, **C1**), or lenalidomide (Figure 2, **D1**–**D3**). However, alternatives are possible, and these include examples with substitution at position 5 of the phthalimide fragment. For organisational purposes and clarity of this section, the complexes between CRBN ligands and linkers are categorized based on the structure of the ligase ligand and further based on the bond type for linker attachment.

#### Pomalidomide-Based Ligands

We categorized the possible synthetic routes based on the common phthalic anhydride precursor, as most syntheses start from either 3-fluorophthalic anhydride, which is then subjected to condensation with the glutarimide ring and subsequent nucleophilic substitution by a linker with a primary amine, or 3-nitrophtalic anhydride, which is subsequently reduced to pomalidomide.

##### 3-Fluorophthalic Anhydride as a Precursor for Pomalidomide-Based Derivatives

**SCHEME 1:**
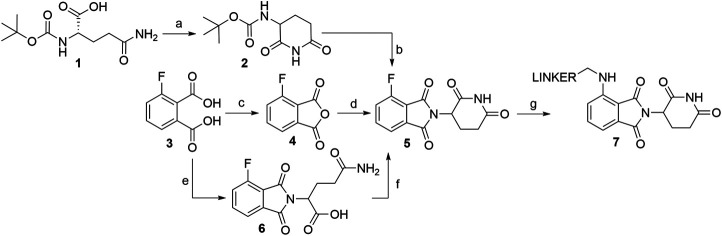
Syntheses of compound **7**. Reagents and conditions: a) CDI, DMAP, THF, reflux, 4 h, 84% yield (Steinebach et al., 2018); b) **4**, NaOAc, AcOH, reflux, 6 h, 89% yield; (Steinebach et al., 2018); c) Ac_2_O, reflux, 2 h, 92% yield (An et al., 2019); d) 3-aminopiperidine-2,6-dione hydrochloride, Et_3_N, THF, 80°C, 69% yield (An et al., 2019); d) 3-aminopiperidine-2,6-dione hydrochloride, NaOAc, AcOH, 140°C, 12 h, 88% yield (Zhou et al., 2018); e) l-glutamine, DMF, 90°C, 8 h, 53% yield (Lu et al., 2015); f) CDI, DMAP, MeCN, reflux, 5 h, 26% yield (Lu et al., 2015); g) reagents, conditions, and yields are collected in Table 1.

Several options are available to obtain pomalidomide-based PROTAC precursors when 3-fluorophthalic anhydride (**4**) is used as the main synthon. The glutarimide subunit can be incorporated into 4-fluorothalidomide (**5**) by using compound **2**, which can be formed easily by converting Boc-Gln-OH (**1**) into **2** *via* an intramolecular coupling (Scheme 1, steps a-b) (Steinebach et al., 2018). Another option to afford the desired precursor **2** over three steps with a 57% yield was presented where l-glutamine was used as starting material (Varala and Adapa, 2005). Alternatively, 3-aminopiperidine-2,6-dione hydrochloride can be used in place of **2** (Zhou et al., 2018; An et al., 2019). The 3-fluorophthalic anhydride (**4**) is usually used as a commercially available building block. However, it can be easily prepared in high yield by refluxing 3-fluorophthalic acid (**3**) in acetic anhydride (Zhou et al., 2018). Following condensation of the glutarimide subunit with 3-fluorophthalic anhydride (**4**)**,** 4-fluorothalidomide (**5**) was obtained in a higher yield by using NaOAc in AcOH under reflux conditions (Steinebach et al., 2018; Zhou et al., 2018), rather than by a method using Et_3_N in THF at 80°C (An et al., 2019) (Scheme 1, step d). An alternative synthesis towards **5** was reported where l-glutamine was reacted with 3-fluorophthalic acid (**3**) to form **6**, followed by a CDI-mediated intramolecular cyclization (Scheme 1, steps e–f). However, the desired product **5** was obtained in an approximately 14% overall yield using this approach (Lu et al., 2015). Compound **5** then allows for simple linker introduction using primary amines and DIPEA in DMF at 90°C, leading to alkylated pomalidomide derivatives **7** (Scheme 1, step g) (Lu et al., 2015; Steinebach et al., 2018; An et al., 2019). It was reported to replace DMF with DMSO for the linker attachment step because of the thermal decomposition of DMF at high temperatures in the presence of a tertiary amine, forming dimethylamine, which can result in the formation of the undesired 4-(dimethylamino)-thalidomide (Steinebach et al., 2018; Steinebach et al., 2019). Recent advances showed that performing the nucleophilic aromatic substitution of compound **5** with primary or secondary amines at elevated temperatures (130°C) generally resulted in a higher yield of desired pomalidomide derivatives (Brownsey et al., 2021).

**TABLE 1:** Reagents, conditions, and yields for converting compound 5 to 7 (Scheme 1, step g).

Paper	Reagents and conditions	Yield
Steinebach et al. (2019)	LINKER-NH_2,_ DIPEA, DMSO, 90°C, 10 h	18–76% for linkers used
An et al. (2019)	LINKER-NH_2,_ DIPEA, DMF, 90°C, 6 h	25% yield for linkers used
Lu et al. (2015)	LINKER-NH_2,_ DIPEA, DMF, 90°C, 12 h	31% yield for linkers used
Brownsey et al. (2021)	LINKER-NH_2_, DIPEA, DMSO, 130°C, 16 h	43–92% yield for linkers used

**SCHEME 2:**
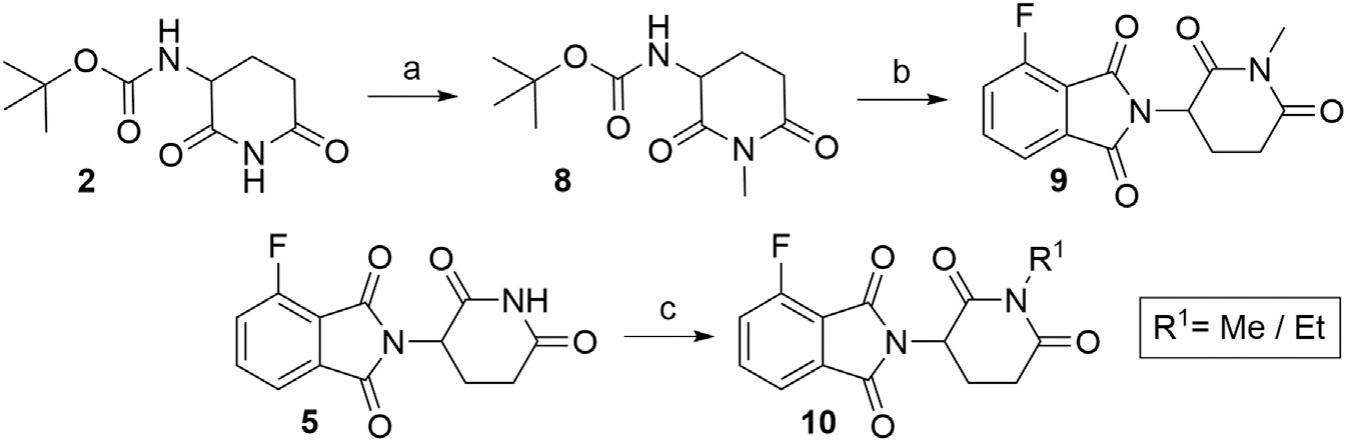
Syntheses of compounds **9** and **10**, precursors to prepare negative controls. Reagents and conditions: a) K_2_CO_3_, MeI, DMF, rt, 2 h, 29% yield; b) **3**, NaOAc, AcOH, reflux, 6 h, 82% yield (Steinebach et al., 2018); c) MeOH, PPh_3_, DIAD, THF, sonication bath, 1 h, 25% yield (Steinebach et al., 2018); c) EtI, K_2_CO_3_, acetone, reflux, 3 h, 65% yield (An et al., 2019).

Numerous studies include thalidomide derivatives with *N*-alkylated glutarimide ring (e.g., compounds **9** and **10**, Scheme 2) as negative controls since they are incapable of binding to CRBN (Buhimschi et al., 2018). Two options are presented for synthesizing such negative controls, the first being the alkylation of glutarimide moiety **8** before conjugation into the final 4-fluorothalidomide (**9**) (Steinebach et al., 2018). Alternatively, the imide nitrogen of **5** can be alkylated after the condensation of glutarimide and phthalimide parts (An et al., 2019), or a methyl group can be introduced *via* Mitsunobu reaction (Steinebach et al., 2018).

**SCHEME 3:**
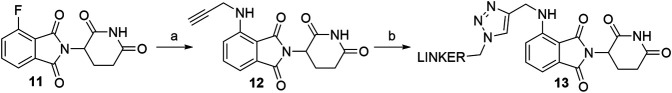
Preparation of pomalidomide derivative available for azide-alkyne cycloaddition click reaction. Reagents and conditions: a) propargylamine, DIPEA, DMF, 90°C, 12 h, 30% yield; b) linker-N_3_, CuSO_4_, Na ascorbate, H_2_O/*t*-BuOH, rt, 16 h, 40–83% yield for linkers used (Wu et al., 2019).

A significant number of reported PROTACs incorporate a triazole fragment (e.g., compound **13**, Scheme 3; compound **34**, Scheme 7; compound **39**, Scheme 8) as a result of utilizing click reactions between azides and alkynes (e.g., compound **12** in Scheme 3), under conditions for a typical copper-catalyzed Huisgen 1,3-dipolar cycloaddition. Deemed the ‘privileged scaffold for PROTACs’, triazoles represent numerous advantages since they are easily accessible in high yields under mild reaction conditions, which are highly compatible with other functional groups (Xia et al., 2019).

##### 3-Nitrophthalic Anhydride as a Precursor for Pomalidomide-Based Derivatives

**SCHEME 4:**
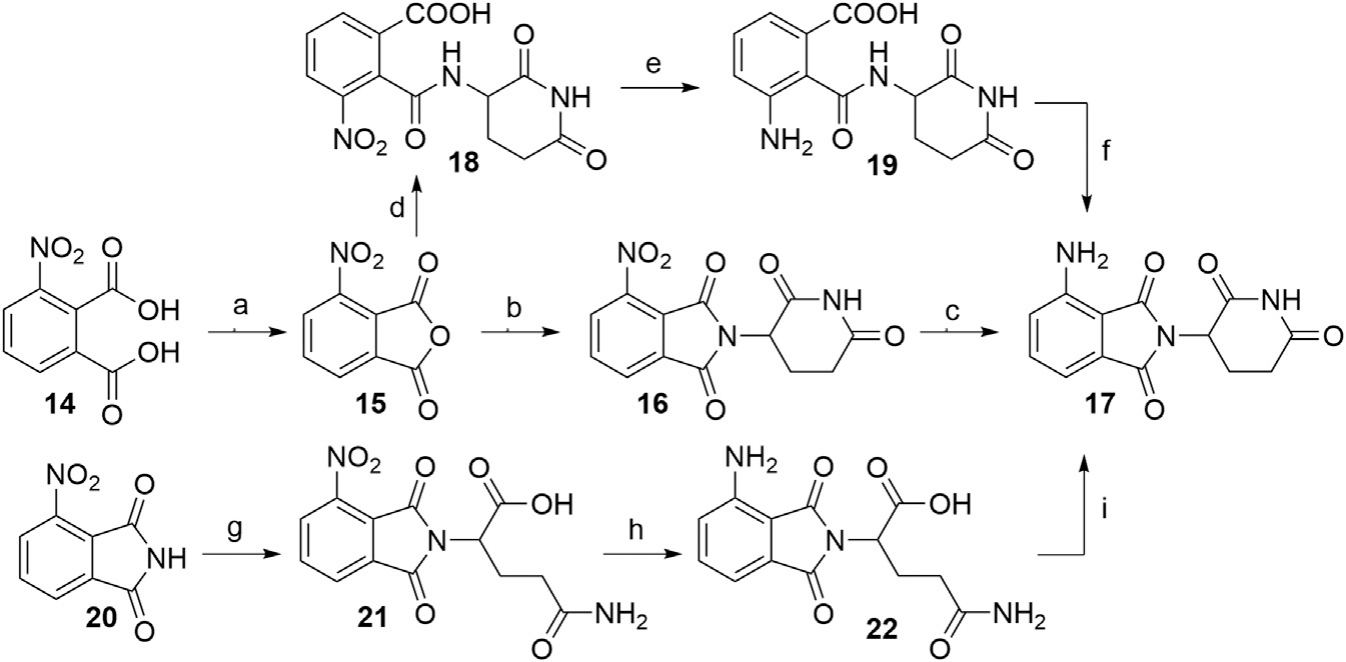
Alternative routes for the synthesis of pomalidomide **17**. Reagents and conditions: a) Ac_2_O, reflux, 2 h, 93% yield (Huang et al., 2016); b) *tert*-butyl (2,6-dioxopiperidin-3-yl)carbamate **2**, NaOAc, AcOH, reflux, 6 h, 95% yield (Steinebach et al., 2018); b) 3-aminopiperidine-2,6-dione hydrochloride, NaOAc, AcOH, 130°C, 48 h, 92% yield (Rana et al., 2019); b) 3-aminopiperidine-2,6-dione trifluoroacetate, Et_3_N, THF, 80°C, 6 h, 69% yield (Chen et al., 2018); b) 3-aminopiperidine-2,6-dione hydrochloride, NaOAc, AcOH, 80°C, 12 h, 73% yield (Huang et al., 2016); c) Pd/C, H_2_, DMF, rt, 24 h, 99% yield (Steinebach et al., 2018); c) Fe, NH_4_Cl, EtOH/H_2_O, rt, overnight, 44% yield (Chen et al., 2018); c) HCOONH_4_, Pd/C, MeOH, rt, 2 h, 68% yield (Huang et al., 2016); d) 3-aminopiperidine-2,6-dione hydrochloride, Et_3_N, THF, ≤20°C, 30 min, 91% yield; e) Pd/C, H_2_, 145 psi, MeOH, rt, 30 min, quant.; f) MeOH, reflux, 2 h, 71% yield (Huang et al., 2016); g) glutamine, DMF, 80–87°C, 8 h, 70% yield (Huang et al., 2016); h) Pd/C, H_2_, 50 psi, MeOH, 2.5 h, 10% yield; i) CDI, MeCN, reflux, 4.5 h, 88% yield (Huang et al., 2016).

When 3-nitrophthalic anhydride (**15**) was used as the starting compound (either commercially available or prepared from **14**) (Huang et al., 2016), it was usually immediately condensed with the glutarimide ring into 4-nitrothalidomide (**16**). Similarly to synthesizing 4-fluorothalidomide (**5**), this condensation yielded the desired product in a significantly higher yield if the reaction was performed under NaOAc, AcOH/reflux conditions (Steinebach et al., 2018; Rana et al., 2019), instead of treating the mixture with Et_3_N in THF (Chen et al., 2018). The following reduction to pomalidomide (**17**) was notably more efficient using Pd/C-catalyzed hydrogenation with reports of near quantitative yield (Steinebach et al., 2018), in contrast to using iron-ammonium chloride (Chen et al., 2018) or Pd/C and ammonium formate (Huang et al., 2016) as reducing agents (Scheme 4, steps a–c). Another option to obtain **17** is through the synthesis of intermediate **18**, its reduction to **19**, and final cyclization to the desired product with an overall yield of 65% (Scheme 4, steps d–f). Although reported to be efficient, practical and environmentally friendly (Huang et al., 2016), the yield was still inferior to the route *via* **16**, which had an overall yield of 94% (Steinebach et al., 2018). Alternatively, Huang et al. reported a method where the 3-nitrophthalimide (**20**) was first reacted with glutamine *via* amination to yield **21**, which was then reduced to **22**, and finally cyclized under CDI-mediated conditions. This resulted in a low pomalidomide (**17**) yield, mostly due to the poor reduction conversion (Scheme 4, steps g–i) (Huang et al., 2016).

**SCHEME 5:**
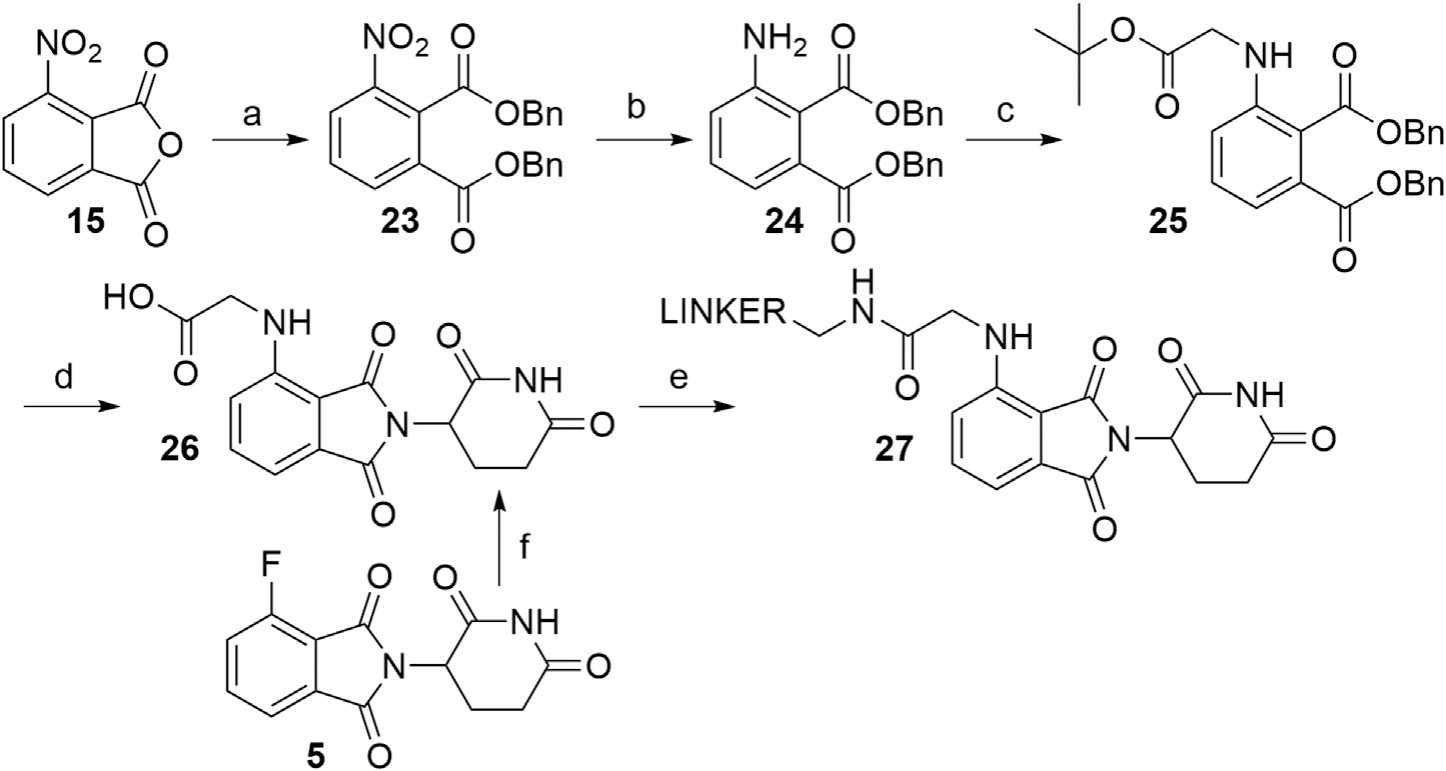
Alternative route to pomalidomide derivatives, containing a two carbon spacer. Reagents and conditions: a) i. TsOH × H_2_O, BnOH, 100°C, 12 h; ii. BnBr, KI, KHCO_3_, DMF, 100°C, 6 h, 80% yield; b) SnCl_2_ × 2 H_2_O, EtOAc, 50°C, 12 h; 90% yield; c) *tert*-butyl bromoacetate, DIPEA, DMF, 90°C, 12 h, 40% yield; d) i. Pd/C, H_2_, EtOH, rt; ii. 3-aminopiperidine-2,6-dione hydrochloride, pyridine, 110°C, overnight; iii. TFA, rt, 2 h, 40% yield for 3 steps; e) linker-NH_2_, HATU, DIPEA; DMF; rt, 2 h, 75% yield for linker used (Zhou et al., 2018); f) i. *tert*-butyl 2-aminoacetate, DIPEA, DMSO, 90°C, 24 h, 68% yield; ii. TFA, CH_2_Cl_2_, rt, overnight, quant (Powell et al., 2018).

The route towards pomalidomide derivatives with a two-carbon spacer was nicely elaborated (Zhou et al., 2018) (Scheme 5). Treating 3-nitrophthalic anhydride (**15**) with benzyl alcohol and benzyl bromide, reducing the nitro group with stannous chloride, and alkylating the resulting amine group with *tert*-butyl bromoacetate yielded compound **25**. This intermediate was then condensed with 3-aminopiperidine-2,6-dione hydrochloride into an *N*-alkylated pomalidomide derivative **26**, containing a two-carbon spacer, that allows the attachment of primary amine linkers *via* the formation of an amide bond (Scheme 5, steps a–e) (Zhou et al., 2018). Alternatively, the synthesis of **26** was described by treating 4-fluorothalidomide (**5**) with *tert*-butyl 2-aminoacetate and cleaving the protecting ester with TFA in a 68% overall yield (Scheme 5, step f) (Powell et al., 2018).

##### Linker Attachment to Pomalidomide

**SCHEME 6:**
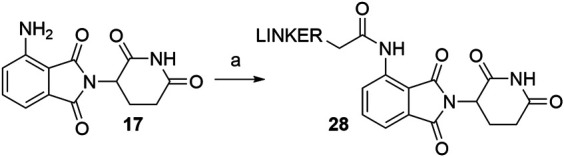
Linker attachment to pomalidomide (**17**) through amide bond formation. Reagents and conditions: a) linker-COCl, THF, reflux, various times and yields (Lai et al., 2016; Buhimschi et al., 2018; Li et al., 2018; Rana et al., 2019).

Coupling pomalidomide (**17**) with the desired linkers was described by numerous authors, utilizing various acyl chloride-bearing linkers in THF under reflux for a various amount of time (Scheme 6). The exact conditions and reported yields are collected in Table 2 and may provide a better understanding of the achievable yield range (Lai et al., 2016; Buhimschi et al., 2018; Li et al., 2018; Rana et al., 2019, 6). It should be noted that a side reaction can occur, i.e., acylation of the imide nitrogen as described (Man et al., 2003). In contrast, alkylation of pomalidomide with alkyl halides is considered to be an inferior strategy for linker attachment due to the low yield and poor chemoselectivity of the reaction (Brownsey et al., 2021).

**TABLE 2:** Reaction times and yields for the conversion of compound **17** to **28** (Scheme 6).

Paper	Reagents time	Yield
Buhimschi et al. (2018)	4 h	86–95% yield for linkers used
Rana et al. (2019)	Overnight	78% yield for linker used
Lai et al. (2016)	4 h	58–91% yield for linkers used
Li et al. (2018)	4 h	58–63% yield for linkers used

**SCHEME 7:**
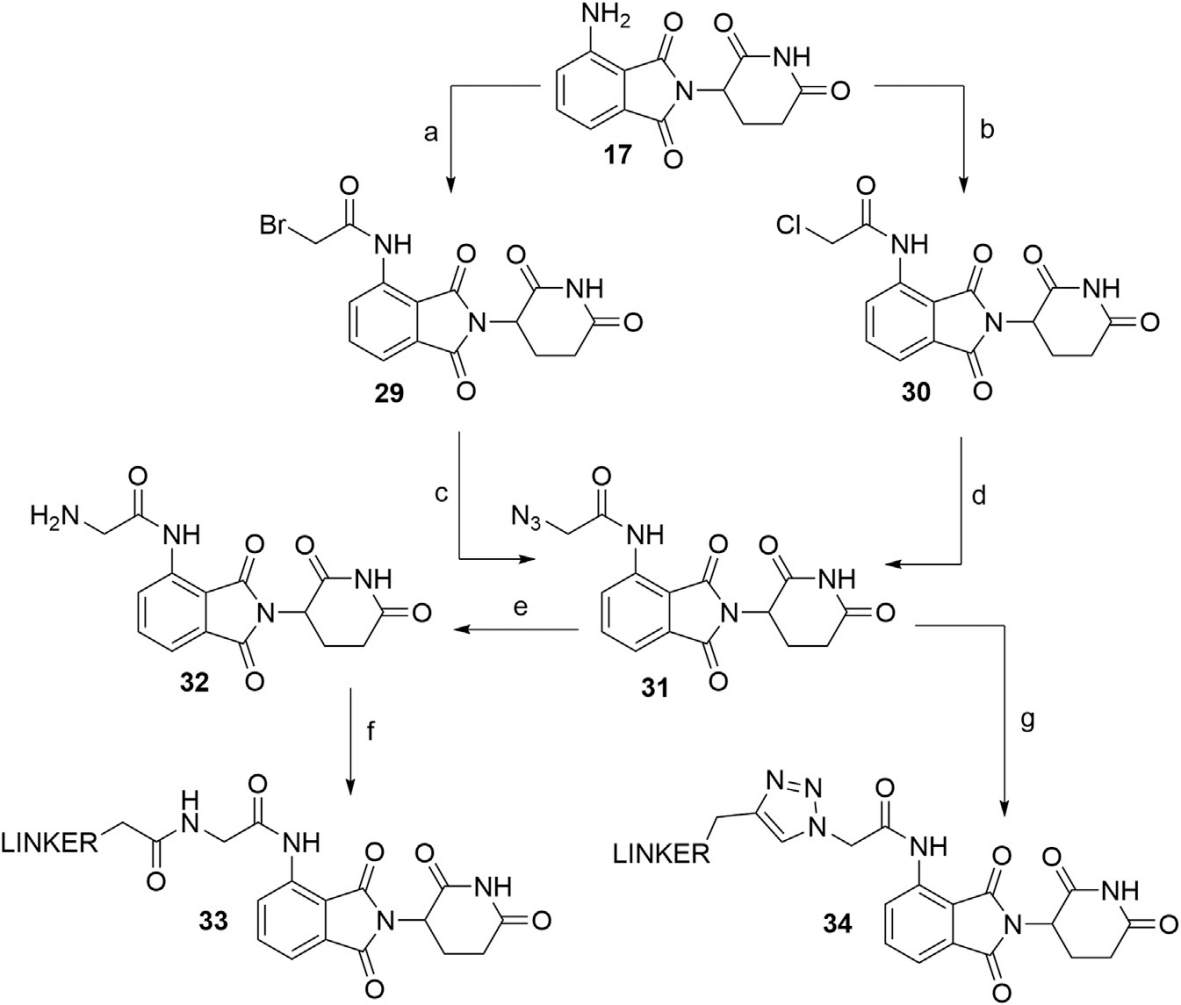
Alternate linker attachment to pomalidomide **17** through amide bond formation. Reagents and conditions: a) bromoacetyl chloride, THF; reflux, overnight, 97% yield (Chen et al., 2019); b) chloroacetyl chloride, THF, reflux, overnight, 87% yield (Chen et al., 2018); c) NaN_3_, acetone, reflux, overnight, 87% yield (Chen et al., 2019); d) NaN_3_, acetone, reflux, overnight, 76% yield (Chen et al., 2018); e) Pd/C, H_2_, MeOH, rt, 6 h, 64% yield; f) carboxylic acid linker, TBTU, Et_3_N, DMF, 50°C, 24 h, 42–59% yield for conjugates used (Chen et al., 2019); g) linker-CH_2_-C≡CH, CuSO_4_ × 5 H_2_O, sodium ascorbate, THF/H_2_O, rt, overnight, 42–80% yield for linkers used (Chen et al., 2018).

As an alternative approach to *N*-acylated derivatives, pomalidomide (**17**) was reacted with bromoacetyl chloride to obtain **29** or chloroacetyl chloride to obtain **30**. Compounds **29** and **30** were then refluxed with NaN_3_ in acetone overnight to form azide **31** in a 84% (Chen et al., 2019) and 76% (Chen et al., 2018) yield over two steps. The azide was then reduced to amine **32**, which presents an attachment point for carboxylic acid linkers *via* amide bond formation (Scheme 7, steps e–f) (Chen et al., 2019). On the other hand, azide **31** was also subjected to click reaction conditions together with a propargyl linker-POI ligand conjugate to form a triazole ring and final PROTAC compounds of type **34** (Scheme 7, step g) (Chen et al., 2018).

##### 5-Aminothalidomide Derivatives

**SCHEME 8:**
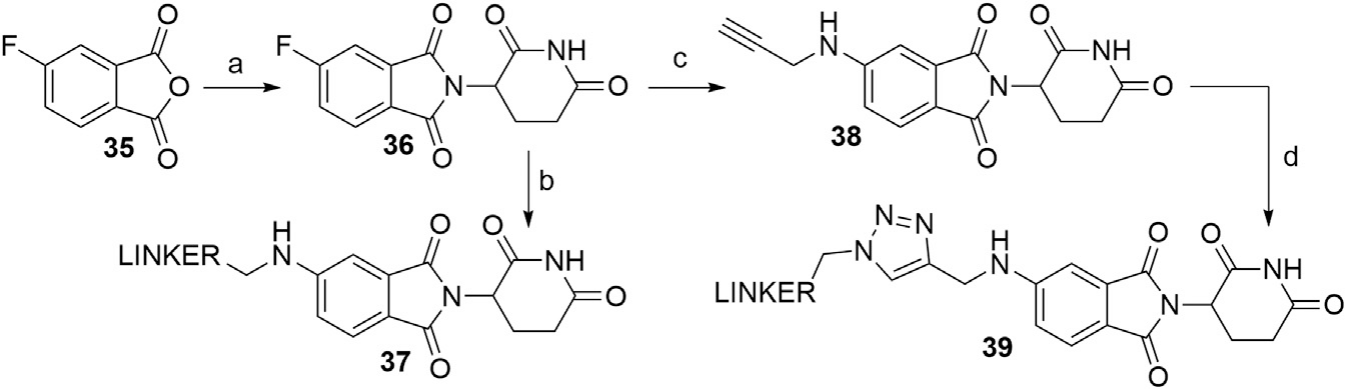
Syntheses of 5-aminothalidomide-based conjugates. Reagents and conditions: a) 3-aminopiperidine-2,6-dione hydrochloride, KOAc, AcOH, 90°C, overnight, 88% yield; b) linker-NH_2_, DIPEA, NMP, 90°C, overnight, 12–23% yield for linkers used (Ishoey et al., 2018); c) propargylamine, DIPEA, DMF, 90°C, 12 h, 17% yield; d) linker-N_3_, CuSO_4_ × 5 H_2_O, Na ascorbate, H_2_O/*t*-BuOH, rt, 16 h, 30–49% yield for linkers used (Wu et al., 2019).

Derivatives of 5-aminothalidomide are less commonly utilized in PROTACs, despite that this substitution pattern still presents a valid option for targeting CRBN (Sun X. et al., 2019). Reagents, conditions and yields for the synthesis of 5-fluorothalidomide (**36**) are comparable to those in Scheme 1 for the preparation of the 4-fluoro analog. The introduction of primary amine linkers by heating the mixture of **36** and the chosen linker alongside DIPEA to obtain 5-aminothalidomide derivatives **37** has been reported (Scheme 8, step b) (Ishoey et al., 2018). Interestingly, the yields for this aromatic nucleophilic substitution were notably lower in comparison with reactions on a 4-fluoro analog. Using propargylamine as the nucleophile provided compound **38**, which again offered a facile option for attaching an azide linker-POI ligand conjugate to form final PROTACs of type **39** (Wu et al., 2019) (Scheme 8, steps c–d).

**SCHEME 9:**
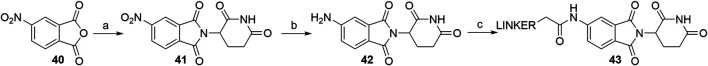
Synthesis of 5-aminothalidomide **42** and linker attachment through amide bond formation. Reagents and conditions: a) 3-aminopiperidine-2,6-dione trifluoroacetate, AcOH, reflux, 2 h, 58% yield; b) Pd/C, H_2_, rt, 20 h, 80% yield (Capitosti et al., 2003); c) linker-COCl, THF, reflux, 4 h, 44% yield for linker used (Note: yield includes a following Finkelstein reaction on the linkers) (Buhimschi et al., 2018).

An alternative synthesis of 5-aminothalidomide (**42**) was achieved by condensing 4-nitrophthalic anhydride (**40**) with 3-aminopiperidine-2,6-dione trifluoroacetate to form **41,** followed by reduction to the desired product **42** (Capitosti et al., 2003) (Scheme 9, steps a–b). Interestingly, the reported yield for the condensation step is lower than those reported for the synthesis of pomalidomide precursor **16** (Scheme 4, step b) (Capitosti et al., 2003). Acyl chlorides were employed to attach the desired linker (Buhimschi et al., 2018) (Scheme 9, step c).

#### 4-Hydroxythalidomide-Based Ligands

##### 4-Hydroxythalidomide with Ether-Bound Linkers

**SCHEME 10:**
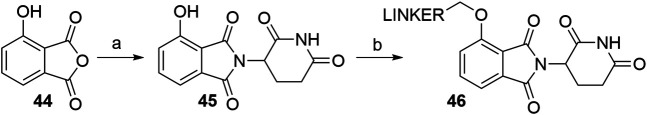
Syntheses of 4-hydroxythalidomide **45** and linker attachment. Reagents and conditions: a) 3-aminopiperidine-2,6-dione hydrochloride, KOAc, AcOH, reflux, 24 h, 96% yield (Robb et al., 2017); a) 3-aminopiperidine-2,6-dione hydrochloride, Et_3_N, toluene, reflux, 12 h, 90% yield (Zhou et al., 2018); a) 3-aminopiperidine-2,6-dione, pyridine, 110°C, 14 h, 88% yield (Jiang et al., 2019); a) i. 3-aminopiperidine-2,6-dione, Et_3_N, DMF, reflux, 4 h; ii. DCC, reflux, 72 h, 83% yield (Papatzimas et al., 2019); a) i. 3-aminopiperidine-2,6-dione hydrochloride, THF, reflux, 24h; ii. EDC, DMAP, reflux, 24 h, 84% yield (Chessum et al., 2018); a) *tert*-butyl (2,6-dioxopiperidin-3-yl)carbamate **2**, CF_3_CH_2_OH, 150°C, 2 h, 86% yield (Burslem et al., 2018); b) reagents, conditions, and yields are collected in Table 3.

Using 3-hydroxyphthalic anhydride (**44**) as starting material, the condensation with the glutarimide ring is possible with various reaction conditions (Scheme 10, step a). The highest yield for the desired product 4-hydroxythalidomide (**45**) was described to be 96% (KOAc, AcOH, reflux) (Robb et al., 2017), whereas yields for other reported procedures span between 83 and 90% (Burslem et al., 2018; Chessum et al., 2018; Zhou et al., 2018; Jiang et al., 2019; Papatzimas et al., 2019), which is comparable to the reactions used for the synthesis of 4-fluoro- and 4-nitrothalidomide (Schemes 1 and 4). Derivatization of **45** (Scheme 10, step b) is possible by attaching iodo (Robb et al., 2017), bromo (Zhou et al., 2018), or tosylate (Rana et al., 2019) groups as linker termini to form ether bond-containing derivatives **46** Linkers with terminal hydroxyl group can be attached to **45** *via* Mitsunobu reaction (Chessum et al., 2018).

**TABLE 3:** Reagents, conditions, and yields for the conversion of **45** to **46** (Scheme 10, step b).

Paper	Reagents and conditions	Yield
Zhou et al. (2018)	Linker-Br, KI, NaHCO_3_, DMF, 60°C, 12 h	76% yield for linker used
Rana et al. (2019)	Linker-OTs, DMF, 80°C, 16 h	43% yield for linker used
Robb et al. (2017)	Linker-I, NaHCO_3_, DMF, 70°C, 6 h	32% yield for linker used (Note: yield includes a following Boc deprotection step)
Chessum et al. (2018)	Linker-OH, PPh_3_, DBAD, THF, rt, 2 h	27–41% yield for linkers used

##### 4-Hydroxythalidomide Used in In-Cell Self-Assembly CLIPTACs

Intracellular formation of PROTAC molecules is possible by the so-called in-cell self-assembly CLIPTACs, an example of which was described for the degradation of bromodomain-containing protein 4 (BRD4) and extracellular signal-regulated kinase 1/2. In this case, 4-hydroxythalidomide was tagged with tetrazine, while the ligands for the POIs were tagged with *trans*-cyclo-octene. The combination of the two precursors underwent a bio-orthogonal click reaction to form the active chimera intracellularly. Utilizing this concept might overcome the cellular permeability issues of some PROTACs since the two small precursor molecules have a higher ability to pass through cellular membranes than one large compound (Lebraud et al., 2016).

**SCHEME 11:**
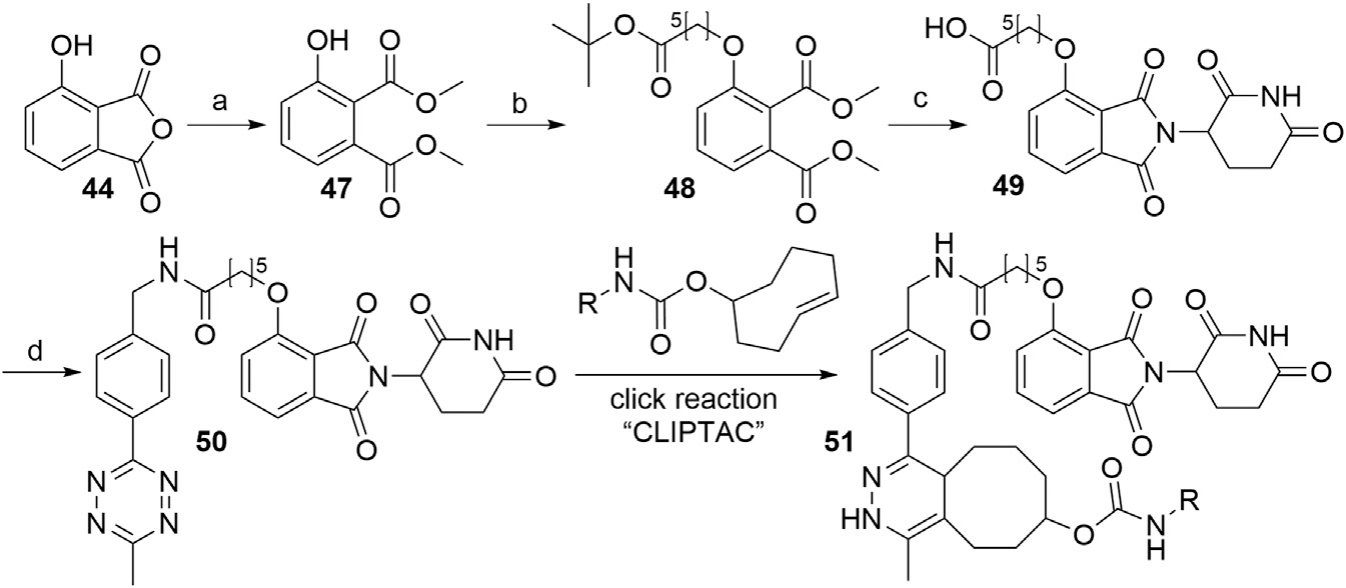
Synthesis of CLIPTAC component **50**. Reagents and conditions: a) i. MeOH, reflux, 3 h; ii. MeI, NaHCO_3_, DMF, 55°C, 3h, 94% yield; b) *tert*-butyl 6-hydroxyhexanoate, PPh_3_, DIAD, THF, rt, 18 h; c) i. 1 M NaOH, THF/MeOH, rt, 2 h; ii. 3-aminopiperidine-2,6-dione hydrochloride, pyridine, 110°C, 17 h; iii. TFA, rt, 3 h, 10% yield over 4 steps; d) methyltetrazine amine, HATU, DIPEA, DMF, rt, 2 h, 57% yield (Lebraud et al., 2016).

As presented in Scheme 11, methanolysis and subsequent methylation of 3-hydroxyphthalic anhydride (**44**) yielded dimethyl ester **47**, which was then alkylated under Mitsunobu conditions leading to *O*-alkylated derivative **48**. This represents an alternative to most syntheses, in which the linker attachment is performed only after the thalidomide portion of the molecule is fully assembled. Basic reaction conditions resulted in the hydrolysis of the methyl esters of **48**, followed by the condensation with the glutarimide ring and *tert*-butyl ester cleavage under acidic conditions to obtain 4-*O*-alkylated thalidomide derivative **49**. Amide coupling for the attachment of the tetrazine moiety yielded compound **50**, which was finally reacted intracellularly with *trans*-cyclo-octene, bound to the POI ligand (Lebraud et al., 2016).

##### 4-Hydroxythalidomide Derivatives With a Two-Carbon Spacer

**SCHEME 12:**
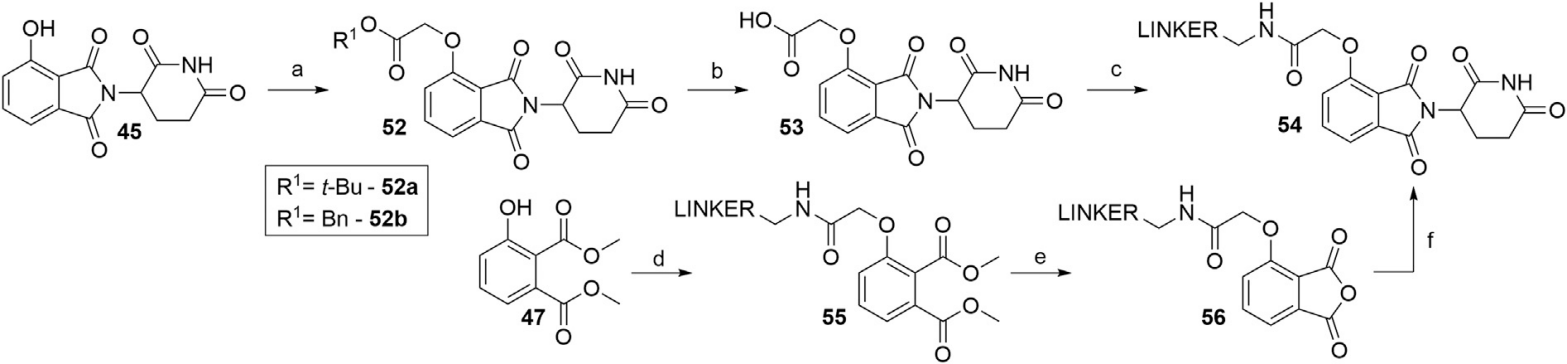
Syntheses of 4-hydroxythalidomide derivatives with a CO-CH_2_ spacer and amide bond-connected linker. Reagents and conditions: a) *tert*-butyl 2-hydroxyacetate, PPh_3_, DTBAD, THF, 0°C to rt, overnight, 75% yield (Chessum et al., 2018); a) *tert*-butyl bromoacetate, KI, KHCO_3_, DMF, 60°C, 12 h, 80% yield (Zhou et al., 2018); a) *tert*-butyl bromoacetate, K_2_CO_3_, DMF, rt, 2 h, 93% yield (Remillard et al., 2017); a) benzyl glycolate, PPh_3_, DIAD, THF, 0°C to rt, 18 h, 73% yield (Lohbeck and Miller, 2016); b) HCO_2_H, CH_2_Cl_2_, 40°C, overnight, 54% yield (Chessum et al., 2018); b) TFA, rt, 4 h, 84% yield (Remillard et al., 2017); b) Pd/C, H_2_, MeOH, rt, 3 h, quant. (Lohbeck and Miller, 2016); c) reagents, conditions, and yields are collected in Table 4; d) linker-CH_2_-NH-CO-CH_2_Cl, Cs_2_CO_3_, MeCN, 80°C, 12 h, 70% yield for linker used; e) 3 M NaOH, EtOH, 80°C, 2 h; f) 3-aminopiperidine-2,6-dione, pyridine, reflux, 12 h, 23% yield for linker used (Note: yield includes a following Boc deprotection on the linker) (Fischer et al., 2014).

Alkylating the 4-hydroxyl group of **45** with *tert*-butyl bromoacetate or benzyl glycolate and subsequent removal of the protecting group produces compound **53**, a standard building block, containing a flexible ‘spacer’, which is ready for linker attachment *via* an amide bond (Scheme 12). The highest yield of over two steps to obtain **53** was reported to be 78% (Remillard et al., 2017). In another study, a yield of only 41% was reached, primarily due to the low conversion rate of Boc-protected derivative **52a** to **53** using formic acid (Chessum et al., 2018). A synthesis of **53** was reported by using benzyl glycolate and Mitsunobu conditions, which yielded the desired product **52b** in 73% (Lohbeck and Miller, 2016). Coupling reaction yields span between 34 and 85% for a selection of different linkers (Table 4) (Lohbeck and Miller, 2016; Remillard et al., 2017; Chessum et al., 2018; Zhou et al., 2018, Fischer et al., 2014). Importantly, the selective alkylation of the phenolic group was confirmed by means of HMBC spectra (Lohbeck and Miller, 2016). Alternatively, a 2-chloro-*N*-acetamide-bearing linker was attached onto phenol **47** to obtain an *O*-alkylated ester **55**. This compound was then first converted to **56**, followed by condensation with 3-aminopiperidine-2,6-dione to form **54** (Scheme 12, steps d–f). The overall yield of this reaction sequence was approximately 16% (Fischer et al., 2014).

**TABLE 4:** Reagents, conditions, and yields for the conversion of **53** to **54** (Scheme 12, step c).

Paper	Reagents and conditions	Yield
Zhou et al. (2018)	Linker-NH_2_, HATU, DIPEA, DMF, rt, 2 h	85% yield for linker used
Chessum et al. (2018)	Linker-NH_2_, HATU, DIPEA, DMF, rt, overnight	72–81% yield for linkers used
Remillard et al. (2017)	Linker-NH_2_, HATU, DIPEA, DMF, rt, 19 h	81% yield for linker used
Lohbeck and Miller (2016)	Linker-NH_2_, HATU, DIPEA, DMF, rt, 2–4.5 h	34–85% yield for linkers used

#### Alkyl-Connected Thalidomide Derivatives

**SCHEME 13:**
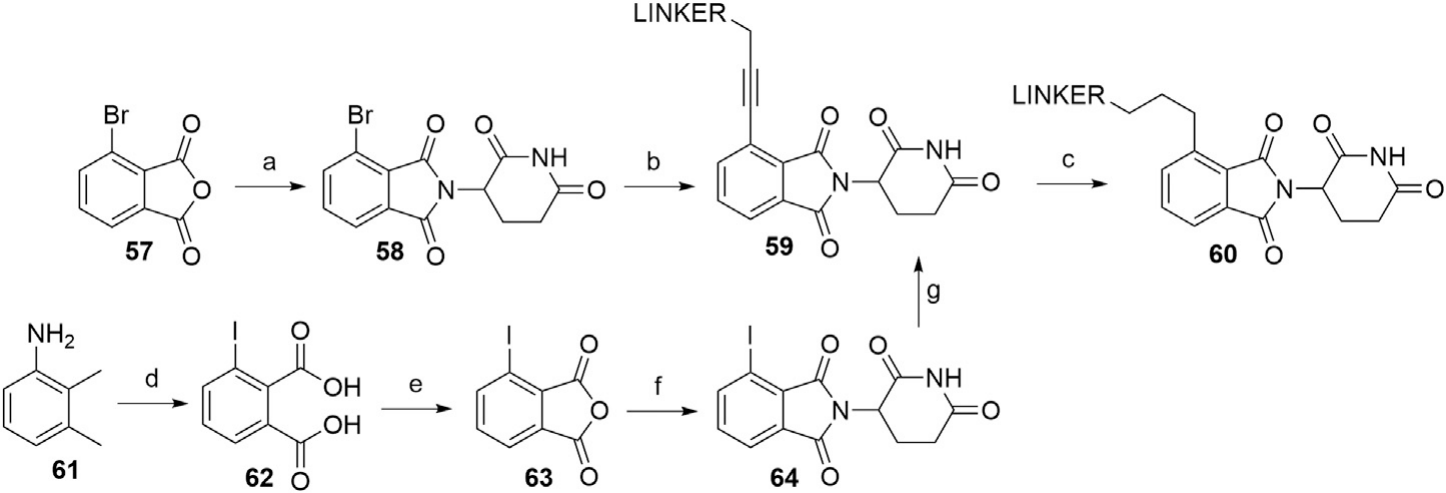
Syntheses of thalidomide derivatives with alkyl-connected linkers. Reagents and conditions: a) 3-aminopiperidine-2,6-dione hydrochloride, NaOAc, AcOH, 140°C, 12 h, 80% yield; b) linker-CH_2_-C≡CH, Pd(PPh_3_)_2_Cl_2_, CuI, Et_3_N, DMF, 70°C, 3 h, 72% yield for linker used (Zhou et al., 2018); b) linker-CH_2_-C≡CH, Pd(PPh_3_)_2_Cl_2_, CuI, Et_3_N, THF, 70°C, 12 h, 87–89% for linkers used (Su et al., 2019); c) Pd/C, H_2_, EtOH, rt, 12 h, (80%. Note: yield includes a following Boc deprotection on the linker) (Zhou et al., 2018); d) i. HCl, NaNO_2_, KI, H_2_O, -15 to 55°C, 5 min, then rt, 16 h, 66% yield; ii. KMnO_4_, H_2_O, 80°C, 4 days, 41% yield; e) Ac_2_O, reflux, 3 h, 68% yield; f) 3-aminopiperidine-2,6-dione trifluoroacetate, Et_3_N, THF, reflux, 24 h, 56% yield (Stewart et al., 2010); f) 3-aminopiperidine-2,6-dione trifluoroacetate, Et_3_N, THF, reflux, 88% yield (Yeung et al., 2011); g) linker-CH_2_-C≡CH, Pd(PPh_3_)_2_Cl_2_, CuI, DIPEA, THF, reflux, 4–22 h, 21–78% yield for linkers used (Stewart et al., 2010).

PROTACs that utilize alkyl-linked thalidomide derivatives to hijack CRBN include both the alkyne-containing linkers **59**, as well as the reduced analogs **60**, which provide either a more rigid or a more flexible connection between the linker and the ligase ligand (Scheme 13) (Zhou et al., 2018; Su et al., 2019). By following the standard procedure for condensation (NaOAc, AcOH, reflux) of the glutarimide ring with 3-bromophthalic anhydride (**57**), 4-bromothalidomide (**58**) was prepared in a straightforward fashion (Zhou et al., 2018)**.** Propargyl-containing linkers were then attached employing Sonogashira coupling to give derivatives **59** in 72–89% yield (Zhou et al., 2018; Su et al., 2019). The alkyne group was then efficiently reduced using Pd/C-catalyzed hydrogenation (Scheme 13, steps a–c) (Zhou et al., 2018). In place of 4-bromothalidomide (**58**), the iodo analog **64** could be used, which was synthesized by subjecting 2,3-dimethylaniline (**61**) to a Sandmeyer-type iodination and potassium permanganate-mediated oxidation to yield **62**. This was then treated with acetic anhydride (forming **63**) and finally combined with the glutarimide moiety to obtain **64** (Scheme 13, steps d–f) (Stewart et al., 2010; Yeung et al., 2011). Relatively lower yields were noted for the Sonogashira coupling using 4-iodothalidomide (**64**) (Stewart et al., 2010), in comparison to reports by other authors for reactions with 4-bromothalidomide (**58**).

#### Lenalidomide-Based Ligands

**SCHEME 14:**
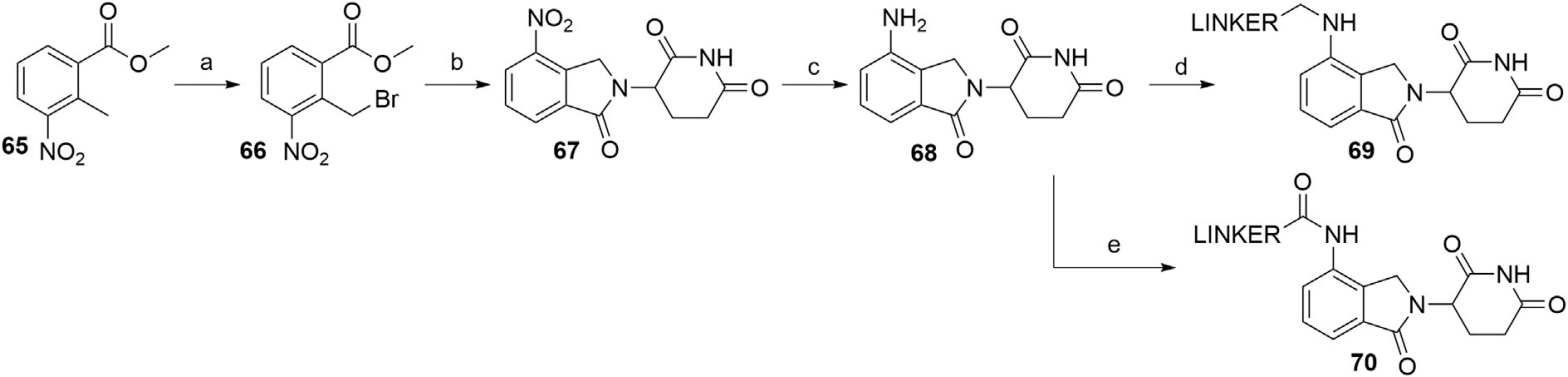
Syntheses of lenalidomide (**68**), *N*-alkylated lenalidomide derivatives **69**, and *N*-acylated derivatives **70**. Reagents and conditions: a) NBS, AIBN, CCl_4_, reflux, 8 h, 88% yield (Balaev et al., 2013); a) NBS, AIBN, MeOAc, reflux, 18 h, 98% yield (Ponomaryov et al., 2015); a) NBS, AIBN, CCl_4_, reflux, 24h, 49% yield (Chaulet et al., 2011); b) 3-aminopiperidine-2,6-dione, Et_3_N, DMF, 50°C, 15 h, 86% yield (Balaev et al., 2013); b) 3-aminopiperidine-2,6-dione hydrochloride, K_2_CO_3_, NMP, 35°C for 1 h, then 55 °C for 18 h, 89% yield (Ponomaryov et al., 2015); 3-aminopiperidine-2,6-dione, Et_3_N, MeCN, 55°C, 18 h, 57% yield (Chaulet et al., 2011); c) Pd(OH)_2_, H_2_, dioxane, 50–60°C, 78% yield (Balaev et al., 2013); c) Fe, NH_4_Cl, H_2_O, EtOH, 80°C, 4 h, 75% yield (Ponomaryov et al., 2015); c) Pd/C, H_2_, MeOH, DMF, quant. (Chaulet et al., 2011); d) linker-I or linker-Br, DIPEA, NMP, 110°C, 12 h, 48–84% yield for linkers used (Note: yield includes a following Boc deprotection on the linker) (Qiu et al., 2019); e) linker-CO_2_H, pyridine, POCl_3_, MeCN, rt, 3 h, about 40% yield for linkers used (Zhang et al., 2020a).

Utilizing lenalidomide-based ligands poses some advantages over using thalidomide and its derivatives to hijack CRBN, as the absence of one phthalimide carbonyl group results in a decreased TPSA, better physicochemical properties, and a higher metabolic and chemical stability (Hoffmann et al., 2013). Additionally, some lenalidomide-based PROTACs displayed a higher level of induced target degradation than their pomalidomide-based counterparts (Qiu et al., 2019). Compound **66** was obtained by bromination of the starting nitrobenzene derivative **65** with *N*-bromosuccinimide (NBS) in CCl_4_ using azobisisobutyronitrile (AIBN) as an initiator of radical bromination, with reported yields of 88% (Balaev et al., 2013) and 49% (Chaulet et al., 2011). An alternative, high-yielding (98%) and green approach for this bromination was presented, where the reaction was carried out in a non-halogenated solvent, i.e., methyl acetate (Ponomaryov et al., 2015). The following condensation with the glutarimide ring was achieved by the addition of a base and heating the solution at 50–55°C, yielding 57% (Et_3_N in MeCN; Chaulet et al., 2011), 86% (Et_3_N in DMF; Balaev et al., 2013), and 89% (K_2_CO_3_ in NMP, Ponomaryov et al., 2015) of the desired nitro product **67**. Optimal conditions for the subsequent reduction to lenalidomide (**68**), i.e., a Pd/C-catalyzed hydrogenation, were described (Chaulet et al., 2011). Alternatives include using Pd(OH)_2_ (Balaev et al., 2013) or iron-ammonium chloride (Ponomaryov et al., 2015), but both procedures led to the product in a lower yield. Selective derivatization of the 4-amino position of lenalidomide (**68**) were performed with of bromo or iodo linkers and DIPEA in NMP at 110°C for 12 h to yield derivatives **69** (Scheme 14) (Qiu et al., 2019). Carboxylic acid linkers were attached *via an* amide bond to form derivatives **70** (Zhang F. et al., 2020).

**SCHEME 15:**
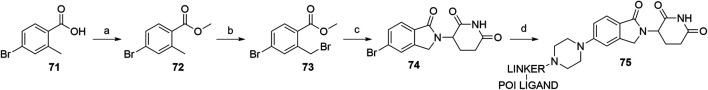
Synthesis of 5-amino derivatives with sterically hindered linker attachment points. Reagents and conditions: a) MeOH, H_2_SO_4_, 65°C, 18 h, 95% yield; b) NBS, AIBN, MeCN, 85°C, 18 h, 66% yield; c) 3-aminopiperidine-2,6-dione hydrochloride, Et_3_N, rt, 25 h, 44% yield (Hansen et al., 2021, 9000); c) 3-aminopiperidine-2,6-dione hydrochloride, DIPEA, MeCN, 80°C, 48 h, 85% yield; d) POI ligand-linker-piperazine conjugate, Pd-PEPPSI-IHept^Cl^, Cs_2_CO_3_, dioxane, 100°C, 3.5 h, 27% yield for linker-POI ligand conjugate used (Hayhow et al., 2020).

Lenalidomide-based ligands are also obtainable through derivatization of 4- and 5-bromo substituted analogs. The synthesis of compound **74** was accomplished from starting with benzoic acid derivative **71**, which was first converted into a methyl ester **72** and then brominated using NBS and AIBN in MeCN to yield compound **73** (Hansen et al., 2021). The following condensation with the glutarimide ring was achieved by the addition of a base, specifically Et_3_N (Hansen et al., 2021) or DIPEA (Hayhow et al., 2020), and 5-bromo lenalidomide derivative **74** was obtained. Subsequent derivatization was possible through Buchwald-Hartwig amination, allowing the attachment of sterically hindered linkers containing a piperazine moiety, which are commonly used in numerous latest PROTACs (Hayhow et al., 2020; Hansen et al., 2021, 9000; Crew et al., 2018a; Crew et al., 2018b). Additionally, the Buchwald-Hartwig protocol with various secondary amines gave yields ranging from 21 to 87% (Scheme 15) (Hayhow et al., 2020).

#### Alkyl-Connected Lenalidomide Derivatives

**SCHEME 16:**
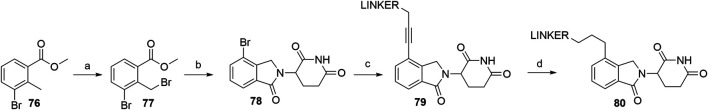
Syntheses of lenalidomide derivatives with alkyl-connected linkers. Reagents and conditions: a) NBS, AIBN, CHCl_3_, reflux, 5 h, 77% yield (Note: the yield was calculated from the following step) (Sun et al., 2019b); a) NBS, dibenzoyl peroxide, benzene, reflux, 6 h, 90% yield (Zhou et al., 2018); b) 3-aminopiperidine-2,6-dione hydrochloride, Et_3_N, THF, 80°C, 6 h, 69% yield (Sun et al., 2019b); b) 3-aminopiperidine-2,6-dione hydrochloride, NaOAc, AcOH, 140°C, 12 h, 88% yield (Zhou et al., 2018); c) reagents, conditions, and yields are collected in Table 5; d) Pd/C, H_2_, MeOH/DMF, rt, 12 h, 63% for linker used (Sun et al., 2019b); d) Pd/C, H_2_, EtOH, rt, 2 h, 85% for linker used (Li et al., 2019).

Similarly to alkyl-connected thalidomide derivatives (“Alkyl-Connected Thalidomide Derivatives” Section), their lenalidomide analogs are used in PROTACs with both the alkyne-type connection (**79**) and the reduced linkage (**80**) (Scheme 16) between the ligase ligand and linker (Su et al., 2019; Wang et al., 2019). The synthesis of these compounds was nicely described recently (Sun Y. et al., 2019). Methyl 3-bromo-2-methylbenzoate (**76**) was subjected to radical bromination using NBS and AIBN in CHCl_3_ to yield **77** (Sun Y. et al., 2019). A higher yield of 90% was reported for a similar radical bromination reaction, where benzene was used as the solvent (Zhou et al., 2018). After condensation with the glutarimide ring to yield bromo-lenalidomide (**78**), linker attachment was achieved through the Sonogashira cross-coupling reaction to afford compounds **79**, with yields spanning between 41 and 81%, depending on the linker used (Sun Y. et al., 2019; Li et al., 2019; Wang et al., 2019). The reduction to **80** was carried out through Pd/C-catalyzed hydrogenation (Sun Y. et al., 2019; Li et al., 2019) (Scheme 16).

**TABLE 5:** Reagents, conditions, and yields for the conversion of **78** to **79** (Scheme 16, step c).

Paper	Reagents and conditions	Yield
Wang et al. (2019)	Linker-CH_2_-C≡CH, Pd(PPh_3_)_2_Cl_2_, CuI, Et_3_N, DMF, 80°C, overnight	81% yield for linker used
Sun et al. (2019b)	Linker-CH_2_-C≡CH, Pd(PPh_3_)_2_Cl_2_, CuI, Et_3_N, DMF, 80°C, 3 h	41% yield for linker used
Li et al. (2019)	Linker-CH_2_-C≡CH, Pd(PPh_3_)_2_Cl_2_, CuI, Et_3_N, DMF, 80°C, overnight	64% yield for linker used

**SCHEME 17:**
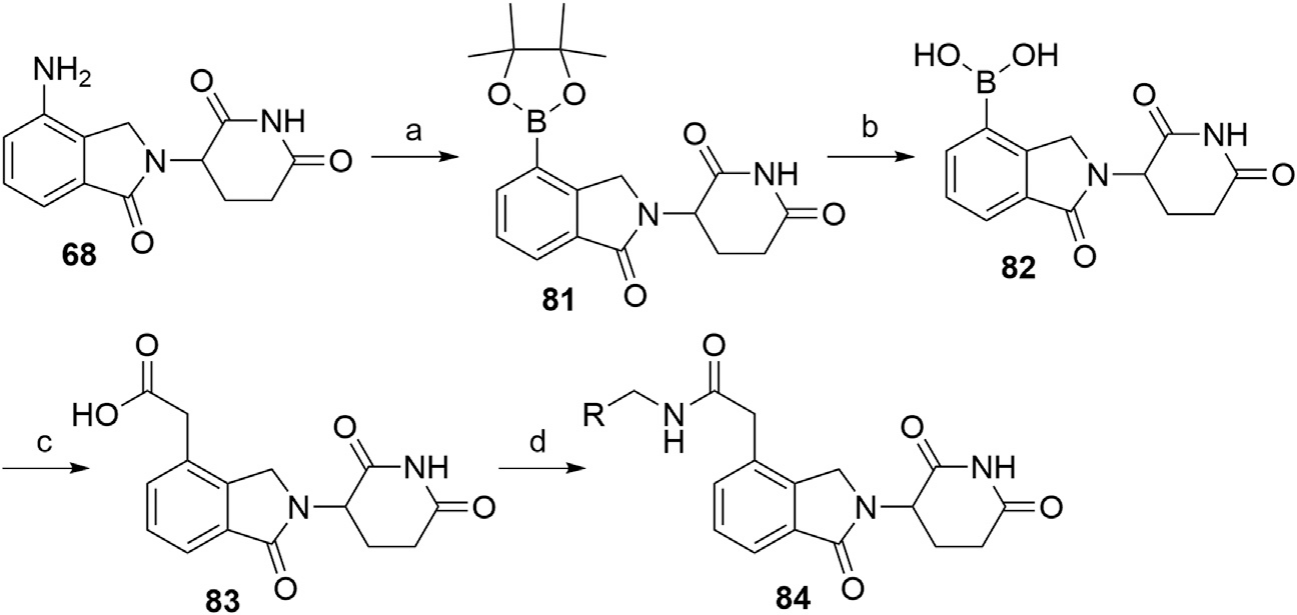
Alternative syntheses of lenalidomide derivatives with alkyl-connected linkers. Reagents and conditions: a) *t*-BuONO, bis(pinacolato)diboron, dibenzoyl peroxide, MeCN, rt, 4 h, 76% yield; b) i. NaIO_4_, THF, H_2_O, rt, 2 h; ii. 1 M HCl, rt, 18 h, 67% yield; c) i. *tert-*butyl bromoacetate, Pd(PPh_3_)_4_, CsF, DME, CH_2_Cl_2_, reflux, 18 h, 46% yield; ii. TFA, CH_2_Cl_2_, rt, 2 h, quant.; d) linker-NH_2_, HATU, DIPEA, DMF, rt, overnight, 66–89% yield for amines used (Xiao et al., 2020).

Alkyl-connected lenalidomide analogs can also be synthesized *via* the Suzuki cross-coupling reaction (Xiao et al., 2020)*.* The amino group of lenalidomide (**68**) group was converted into an arylboronic ester **81** through a metal-free pinacol borylation reaction under Sandmeyer-type transformation (Scheme 17). Compound **82** was obtained through oxidative hydrolysis and then joined with *tert-*butyl bromoacetate by using Pd(PPh_3_)_4_ as a coupling catalyst for Suzuki cross-coupling. Ester hydrolysis afforded compound **83**, which is suitable for amine linker attachment to form compounds **84** (Xiao et al., 2020).

It should be mentioned here that synthetic approaches towards hydroxyl analogs of lenalidomide were disclosed recently (Hansen et al., 2020). Although these compounds were not utilized in PROTACs, the syntheses might prove very useful in further research on lenalidomide-derived degraders.

#### Tricyclic Imide Moiety

**SCHEME 18:**
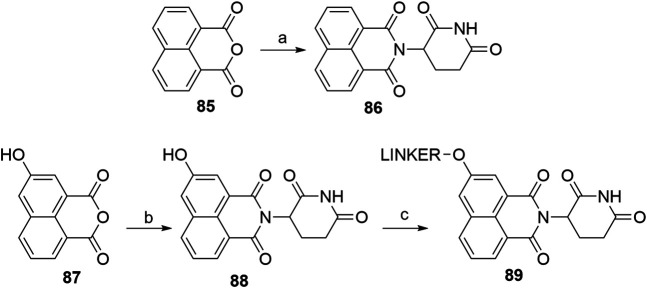
Syntheses of tricyclic imide moiety **86** and its 5-hydroxyl derivative **88**. Reagents and conditions: a) *tert*-butyl (2,6-dioxopiperidin-3-yl)carbamate **2**, CF_3_CH_2_OH, 150°C, 6 h, MW, 80% yield (Burslem et al., 2018); b) 3-aminopiperidine-2,6-dione hydrochloride, THF, 75°C, 1 h (Note: yield not given); c) linker-Br, K_2_CO_3_, DMSO, 50°C, overnight, 22% yield for linker used (Gray et al.,. 2020).

The tricyclic imide moiety **86** (Scheme 18) was used in a single PROTAC, which targeted the NS3 protein in virus hepatitis C. Compound **86** had a higher binding affinity for CRBN and did not result in the degradation of neo-substrates, such as IKZF1 and IKZF3 (de Wispelaere et al., 2019). The condensation of 1,8-naphthalic anhydride (**85**) with the glutarimide ring was performed microwave-assisted (Burslem et al., 2018). 5-Hydroxy derivative **87** was condensed to **88** in a similar way**,** enabling halogen linker attachment to yield an ether bond-connected linker (Gray et al., 2020).

#### Cereblon PHOtochemically TArgeting Chimeras

**SCHEME 19:**
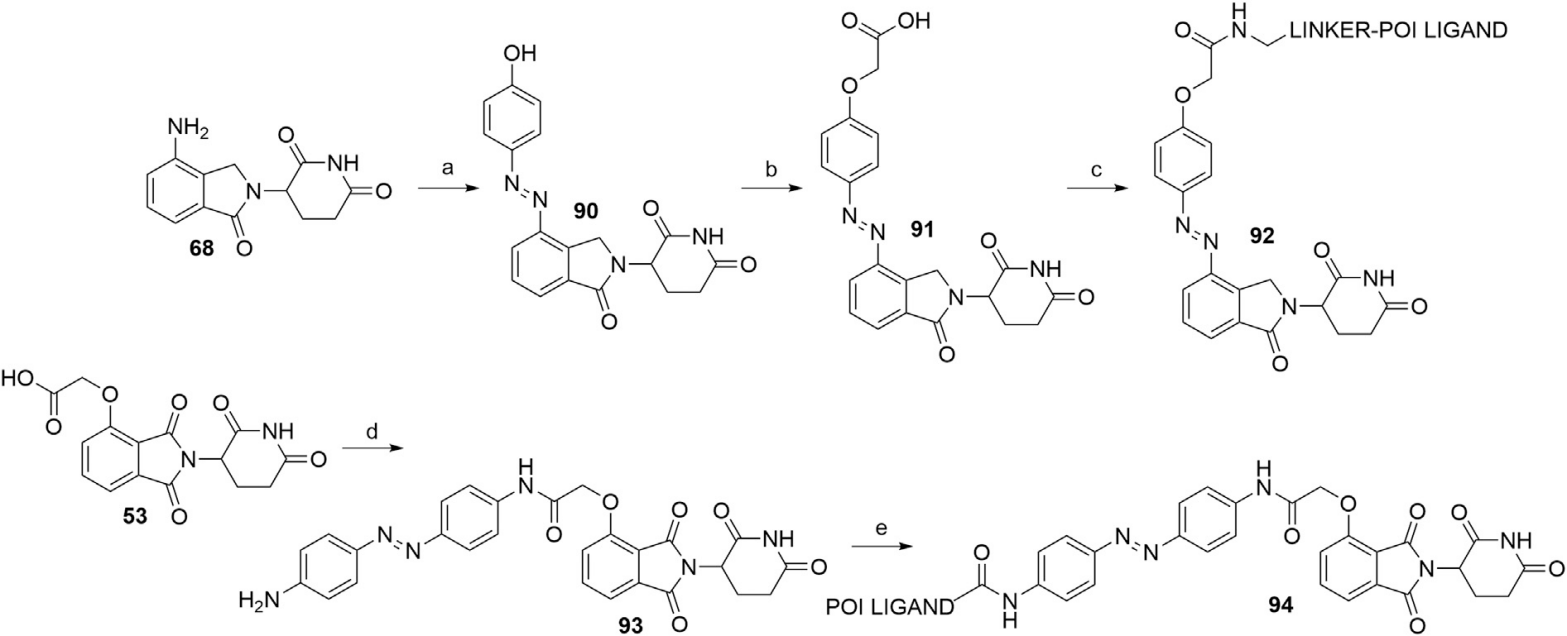
Syntheses of CRBN-targeting PHOTACs **92** and **94**. Reagents and conditions: a) i. 1 M HCl, HBF_4_, 2 M NaNO_2_, 0°C, 1 h; ii. phenol, NaHCO_3_, Na_2_CO_3_, MeOH, H_2_O, 0°C, 1 h, 86% yield; b) i. *tert*-butyl bromoacetate, K_2_CO_3_, DMF, rt, 2.5 h; ii. TFA, CH_2_Cl_2_, rt, 2 h, 56% yield; c) linker-NH_2_, HATU, DIPEA, DMF, rt, 12 h, 92–99% yield for linkers used; d) 4,4′-azodianiline, HOBt, PyBOP, Et_3_N, THF, rt, overnight, 79% yield; e) POI ligand-CO_2_H, HATU, DIPEA, DMF, rt, overnight, 65% yield for the POI ligand used (Reynders et al., 2020).

A recent advance in the field of targeted protein degradation are photoswitchable PROTACs or PHOTACs (PHOtochemically TArgeting Chimeras). In addition to POI and E3 ligase ligands, these compounds possess a photoswitch, which allows them to be reversibly activated with different wavelengths of light, but not display relevant activity in the deactivated conformation. This enables the utilization of PHOTACs as precision therapeutics, capable of avoiding undesired systemic toxicity (Reynders et al., 2020). To date, the strategy has been described twice (Jin et al., 2020; Reynders et al., 2020). Two examples of active PHOTACs (Reynders et al., 2020) are presented in Scheme 19, each incorporating azobenzene photoswitches, with the *trans* configuration presenting the resting, inactive state. The active *cis* isomer can be obtained by irradiation with light of specific wavelengths. In one of the cases, the authors incorporated the azobenzene switch directly to lenalidomide (**68**) to give compound **90**. They then derivatized the hydroxyl group to yield **91**, which enabled amine linker attachment *via* an amide bond. Alternatively, compound **53**, which contains a flexible two carbon spacer, was coupled with 4,4’-azodianiline to give **93**, onto which a POI ligand-linker conjugate with a carboxylic acid was attached to yield **94** (Reynders et al., 2020).

#### Caged Cereblon Ligands

**SCHEME 20:**
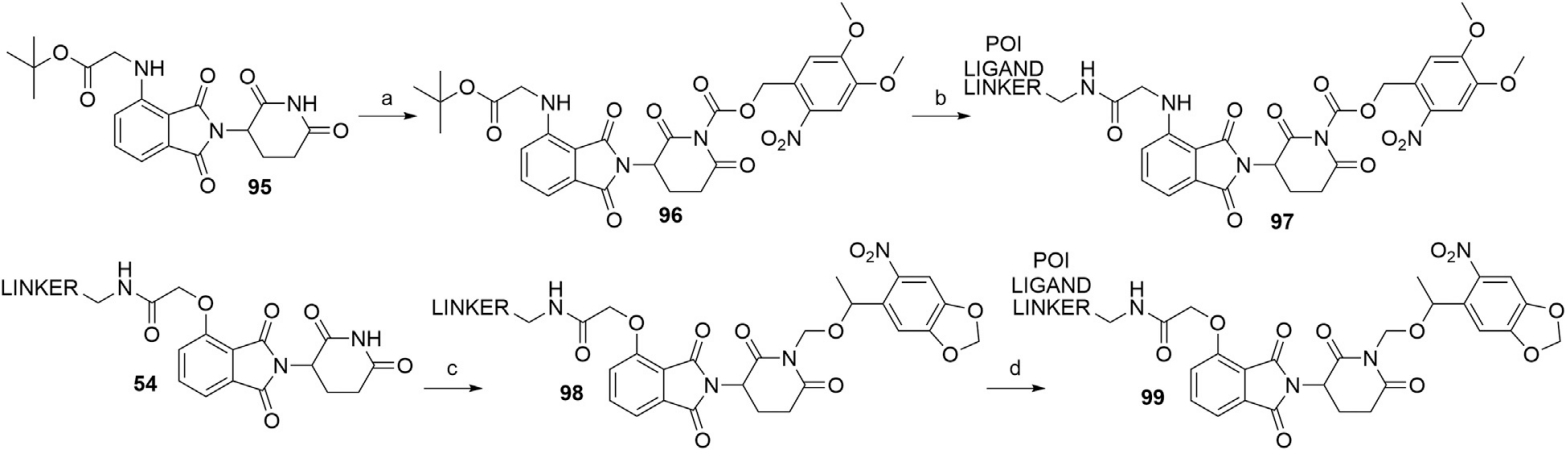
Syntheses of caged CRBN degraders **97** and **99**. Reagents and conditions: a) i. NaHMDS, CH_2_Cl_2_, THF, –80°C to –30°C; ii. 4,5-dimethoxy-2-nitrobenzyl chloroformate, -30°C to rt, 27% yield; b) i. TFA, CH_2_Cl_2_, rt, 2 h; ii. POI ligand-linker-NH_2_, HATU, DIPEA, DMF, rt, overnight, 36% yield for conjugate used (Xue et al., 2019); c) 5-(1-(chloromethoxy)ethyl)-6-nitrobenzo[*d*][1,3]dioxole, DBU, DMF, 0°C to rt, overnight, 80% yield for conjugate used; d) POI ligand-CO_2_H, HATU, DIPEA, DMF, 0°C to rt, 16 h, 73% yield for conjugate used (Naro et al., 2020).

Apart from PHOTACs, an alternative option that enables the control of the location and timing of targeted proteolysis is incorporating a photocleavable group into a motif that is essential for binding to the E3 ligase (Xue et al., 2019; Naro et al., 2020). The imide moiety of thalidomide’s glutarimide ring thus presents an ideal position for attaching a photolabile moiety, such as the nitroveratryloxy-carbonyl (Xue et al., 2019) or 6-nitropiperonyloxymethyl (NPOM) group (Naro et al., 2020). In the former study, the imide moiety of starting material **95** was derivatized to form **96** prior to the linker and POI ligand attachment (Xue et al., 2019), while in the latter study, the NPOM group was attached to a conjugate of 4-hydroxythalidomide and a linker (**54**) to form **98**. The POI ligand was then coupled *via* an amide bond to obtain final PROTAC **99** (Naro et al., 2020) (Scheme 20).

#### Statistical Overview of Utilized Cereblon Ligands

**FIGURE 3:**
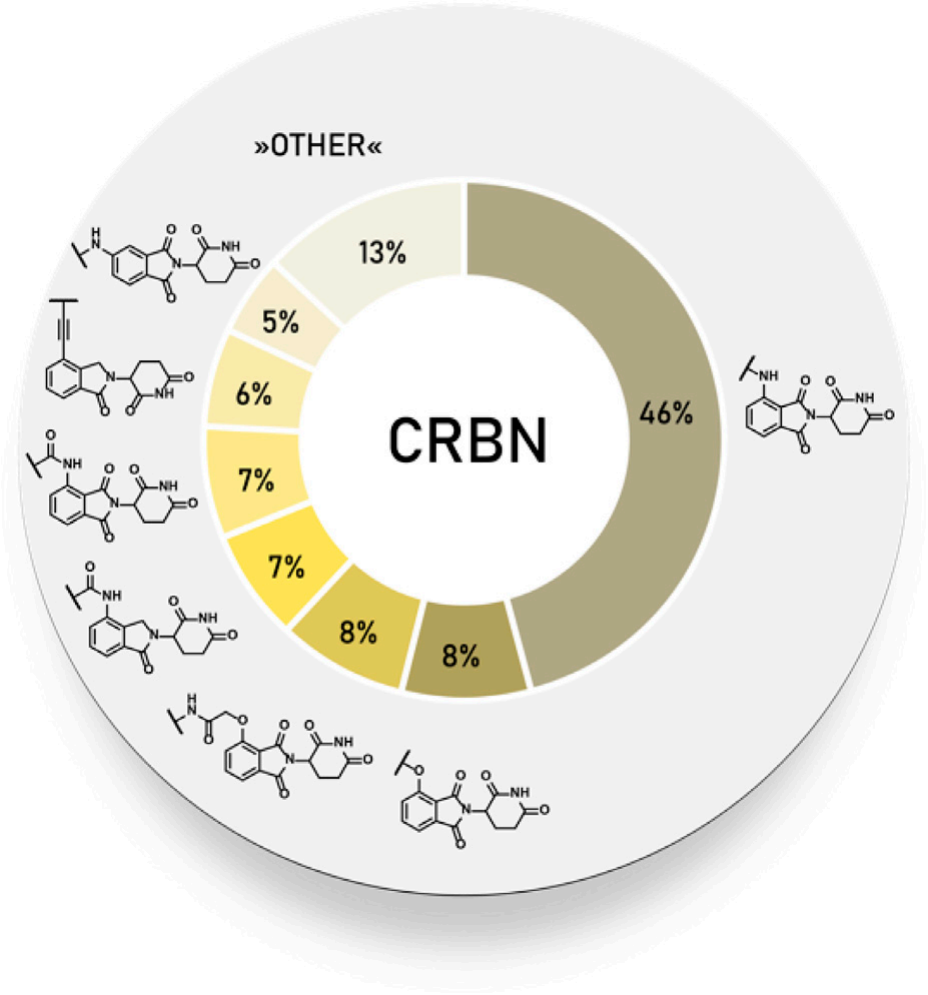
Frequency of CRBN ligands used in PROTAC compounds.

Using data extracted from PROTAC-DB (Weng et al., 2021) (http://cadd.zju.edu.cn/protacdb/, as of the February 26, 2021) a statistical overview was done to determine the frequency of various CRBN ligands and linker attachment options used in PROTAC compounds (Figure 3). An overwhelming majority of PROTACs incorporated an *N*-alkylated pomalidomide as the E3 ligase ligand, while an acylated pomalidomide was about as commonly represented as 4-hydroxythalidomide derivatives. Interestingly, the 5-amino derivative was utilized in around 5% of PROTACs. Lenalidomide analogs, namely 4-acylated derivatives and alkyl-connected lenalidomide derivatives were similarly frequent at 8 and 7%, respectively.

### Von Hippel–Lindau

The VHL protein is a part of the multiprotein complex, along with elongin B and C, cullin 2 and Rbx-1, which possesses an E3 ubiquitin ligase activity. Within the complex, VHL folds into two domains, one of which is responsible for the binding of specific substrates (Czyzyk-Krzeska and Meller, 2004), most notably the hypoxia-inducible factor (HIF)-1α, leading to its ubiquitination and proteasomal degradation. Early VHL-targeting PROTACs utilized 5-7 amino acid long sequences derived from HIF-1α protein (Schneekloth, et al., 2004; Lee et al., 2007), due to the lack of small-molecule VHL ligands. Peptidomimetic binding moieties with high VHL-binding affinity have been developed in 2012 and widely been used in PROTACs ever since (Buckley et al., 2012a; Buckley et al., 2012b; Van Molle et al., 2012). However, a few more recently reported PROTACs still employ a hydroxylated pentapeptide for hijacking VHL (Wang et al., 2016).

**FIGURE 4:**
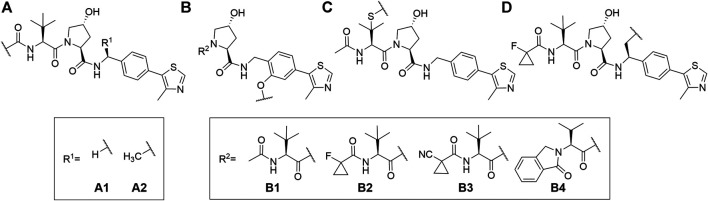
VHL ligands found in PROTACs. Linker attachment options are represented with curly bonds and are: **(A)** *via* an amide bond after *tert*-leucine; **(B)** phenolic linkage point at the benzene ring; **(C)** *via* a thioether at the left-hand side amino acid; **(D)** *via* the benzylic methylene group.

VHL is similarly to CRBN extensively targeted with PROTAC compounds and has been successfully utilized for degrading more than 20 different proteins (Sun X. et al., 2019). The development of VHL ligands with a solid binding affinity included the analysis of co-crystal structures, which helped to locate the solvent-exposed regions. Accordingly, positions that could be derivatized without negatively affecting the critical affinity were identified (Bondeson et al., 2015; Buckley et al., 2015; Zengerle et al., 2015; Maniaci et al., 2017). These include connection *via* an amide bond after the amino acid *tert*-leucine (**A**), phenolic linkage point at the benzene ring (**B**), link *via* a thioether at the left-hand side amino acid (**C**), and *via* the benzylic methylene group (**D**) (Figure 4).

#### A: Connection via an Amide Bond after *tert*-Leucine

##### von Hippel–Lindau Ligand 1

**SCHEME 21:**
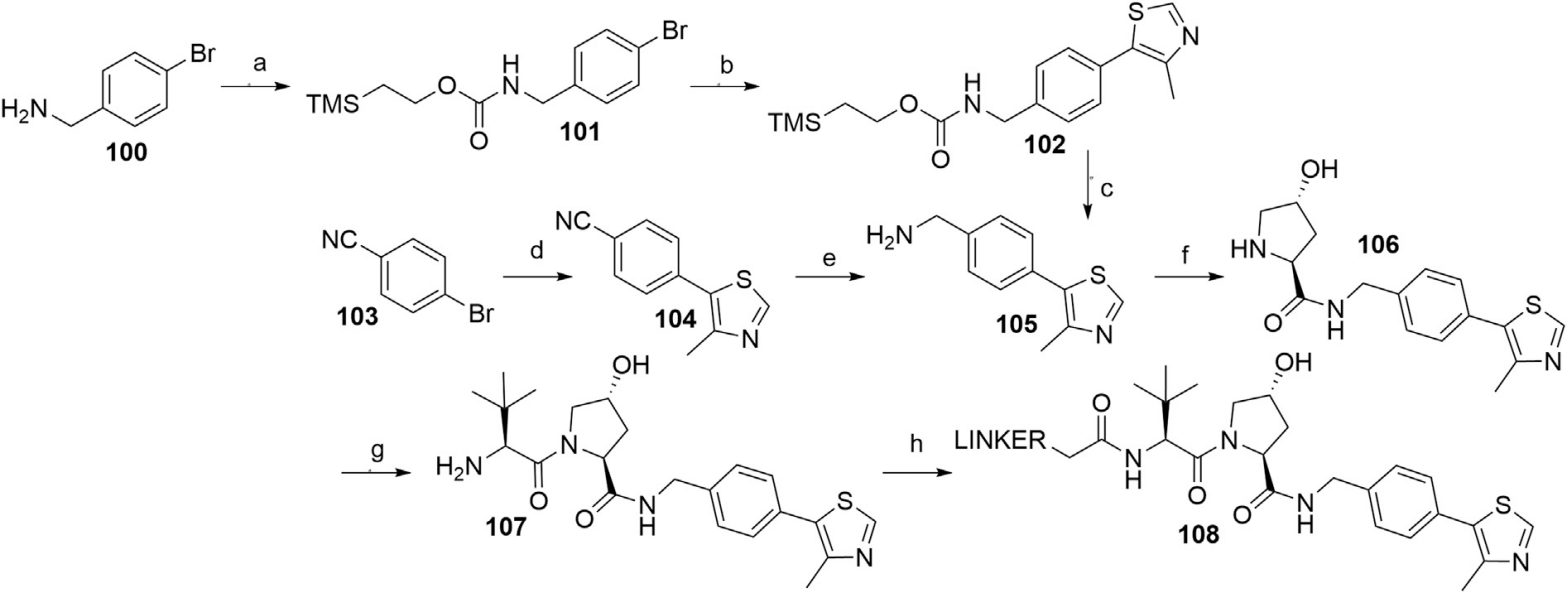
Syntheses of VHL ligand **107**. Reagents and conditions: a) Teoc-OSu, Et_3_N, DMF, H_2_O, rt, 16 h, 84% yield; b) 4-methylthiazole-5-carboxylic acid, Pd(P(*t*Bu)_3_)_2_, Bu_4_NCl × H_2_O, Cs_2_CO_3,_ DMF, MW, 170°C, 8 min, 22% yield; c) TBAF, MeCN, rt, 18 h, 96% yield (Buckley et al., 2012a); d) 4-methylthiazole, KOAc, Pd(OAc)_2_, DMA, 150°C, 5 h, 91% yield (Crew et al., 2018); d) 4-methylthiazole, KOAc, Pd(OAc)_2_, DMA, 150°C, 5 h, 97% yield (Galdeano et al., 2014); d) 4-methylthiazole, KOAc, Pd(OAc)_2_, DMA, 150°C, 19 h, 99% yield (Buckley et al., 2012a); e) LiAlH_4_, THF, reflux, 5 h, 63% yield (Crew et al., 2018); e) NaBH_4_, CoCl_2_, MeOH, 0°C, 90 min, 29% yield (Galdeano et al., 2014); e) NaBH_4_, CoCl_2_, MeOH, 4°C, 90 min, 73% yield (Buckley et al., 2012a); f) i. Boc-Hyp-OH, HATU, DIPEA, DMF, rt, 2 h; ii. HCl, MeOH, rt, 2 h, 41% yield (Crew et al., 2018); f) i. Boc-Hyp-OH, HATU, DIPEA, DMF, rt, 30 min; ii. TFA, CH_2_Cl_2_, rt, 30 min, 93% yield (Galdeano et al., 2014); g) i. Boc-Tle-OH, HATU, DIPEA, DMF, rt, 30 min; ii. TFA, CH_2_Cl_2_, rt, 30 min, 96% yield (Galdeano et al., 2014); h) linker-CO_2_H, HATU, DIPEA, DMF, rt, 2 h, 20% yield for linker used (Crew et al., 2018); h) linker-CO_2_H, HATU, DIPEA, DMF, rt, 16 h, 59–90% yield for linkers used (Steinebach et al., 2020a).

The key intermediate for the synthesis of VHL ligand **107** (i.e., VHL A1) is compound **105**, which can be formed by using a Pd-catalyzed arylation of 4-bromobenzonitrile **103** and subsequent reduction of the nitrile group of **104**, for which an array of methods with varying yields has been published (Buckley et al., 2012a; Galdeano et al., 2014; Crew et al., 2018). LiAlH_4_ was used which resulted in the desired product **105** in 63% yield (Crew et al., 2018), while a NaBH_4_-CoCl_2_ combination led to a 29% conversion at 0°C (Galdeano et al., 2014) and 73% at 4°C (Buckley et al., 2012a). Alternatively, a synthetic strategy was reported comprising the conversion of 4-bromobenzylamine (**100**) into compound **105** in three steps with an overall yield of 18% (Scheme 21, steps a–c) (Buckley et al., 2012a). With compound **105** in hand, the subsequent reaction steps were very straightforward. For example, standard coupling conditions enabled the formation of an amide bond with Boc-l-hydroxyproline, followed by acid-mediated cleavage of the Boc protecting group, which afforded **106**. Finally, an amide bond with Boc-l-*tert*-leucine was formed, and Boc deprotection of the terminal amine yielded compound **107**, which allowed derivatization with carboxylic acid linkers to give conjugates **108** (Scheme 21, steps f–h) (Galdeano et al., 2014; Crew et al., 2018; Steinebach et al., 2020a).

**SCHEME 22:**
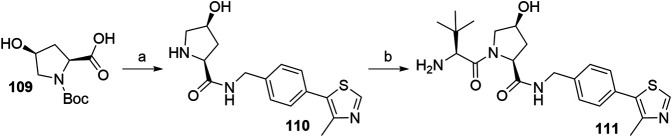
Synthesis of VHL non-binding ligand 111. Reagents and conditions: a) i. 105, HATU, DIPEA, DMF, rt, overnight; ii. HCl, MeOH, rt, 2 h, 53% yield; b) i. Boc-Tle-OH, HATU, DIPEA, DMF, rt, 3 h; ii. HCl, dioxane, rt, 3 h, 50% yield (Crew et al., 2018).

Inverting the configuration at the hydroxyproline moiety results in a loss of binding affinity for VHL, and such modified compounds are mostly incorporated into negative control VHL-based PROTACs (Raina et al., 2016). Using *N*-Boc-*cis*-4-hydroxy-l-proline (**109**) in place of Boc-l-hydroxyproline and coupling with **105** yielded the VHL non-binding ligand **111** (Scheme 22) (Crew et al., 2018).

**SCHEME 23:**
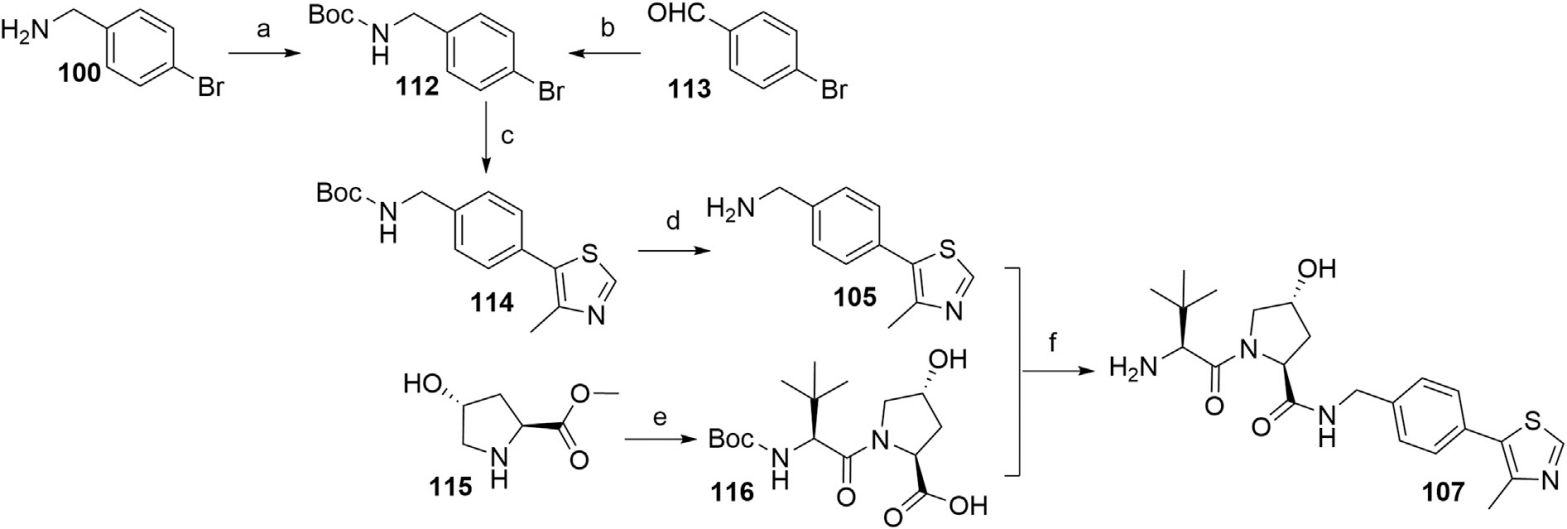
Alternative synthesis of VHL ligand **107**. Reagents and conditions: a) (Boc)_2_O, NaHCO_3_, EtOAc/H_2_O, rt, 1 h, 95% yield (Han et al., 2019); b) *tert*-butyl carbamate, Et_3_SiH, TFA, CH_2_Cl_2_, MeCN, rt, overnight, 83% yield (Steinebach et al., 2020b); c) 4-methylthiazole, Pd(OAc)_2_, KOAc, DMF, 90°C, 2 h, 85% yield; d) TFA, CH_2_Cl_2_, rt, 30 min, 95% yield; e) i. Boc-Tle-OH, HATU, DIPEA, DMF, rt, overnight; ii. LiOH, THF/H_2_O, 85% yield; f) i. HATU, DIPEA, DMF, rt, overnight; ii. TFA, CH_2_Cl_2_, rt, 30 min, 88% yield (Han et al., 2019).

The most efficient procedure for the synthesis of VHL ligand **107** started from 4-bromobenzylamine (**100**) (Scheme 23) which was first Boc-protected to **112** and then underwent the Heck reaction and Boc deprotection to give crucial intermediate **105** (Han et al., 2019). Alternatively, **112** could be prepared through reductive amination of 4-bromobenzaldehyde (Steinebach et al., 2020b). This new synthetic sequence does also allow the introduction of substituents to the central phenylene unit. The final ligand **107** was prepared in a convergent manner by coupling **105** with the dipeptide **116**, which was prepared from hydroxyproline methyl ester (**115**) and *N*-Boc-l-*tert*-leucine. Incorporating an element of convergent synthesis helped to increase the overall yield (Han et al., 2019).

##### von Hippel–Lindau Ligand 2

**SCHEME 24:**
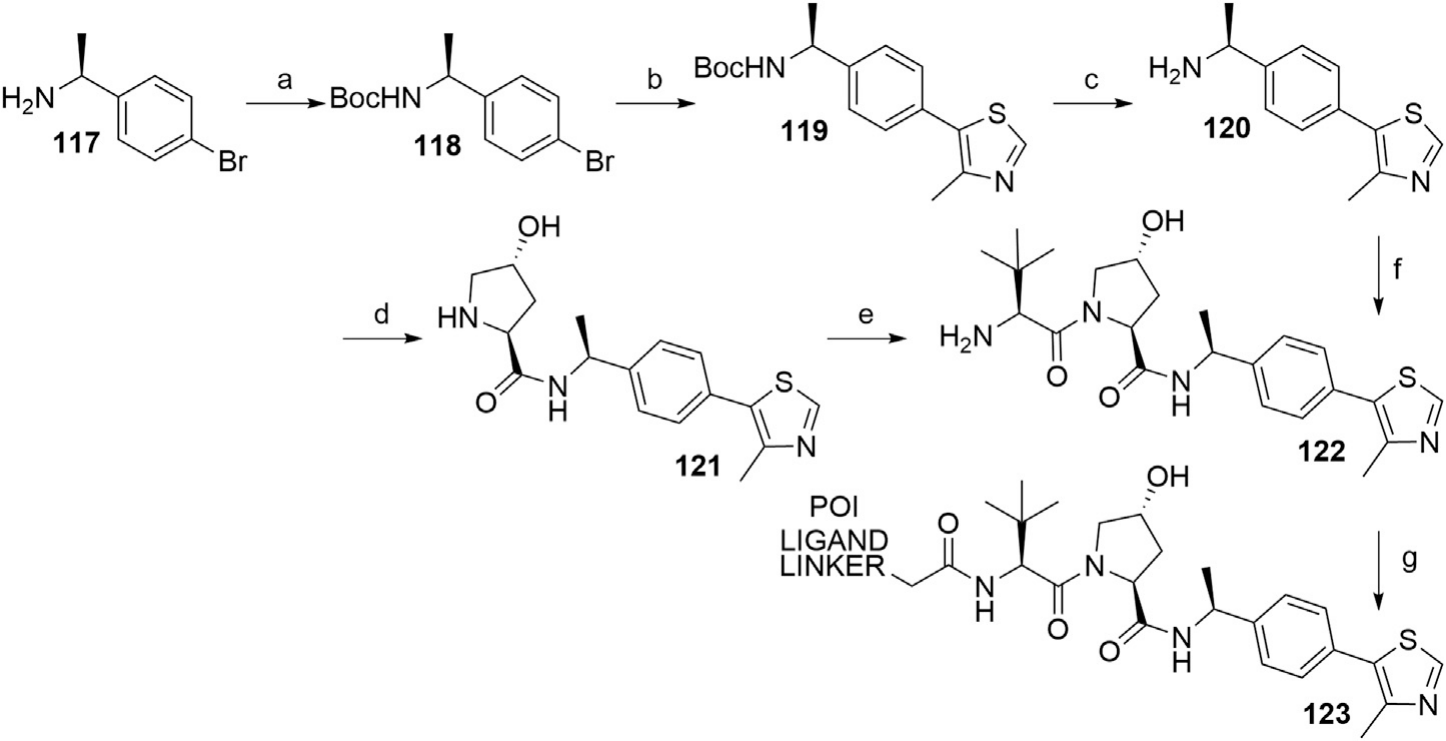
Synthesis of VHL ligand **122** with an (*S*)-methyl group on the benzylic carbon atom of the molecule. Reagents and conditions: a) (Boc)_2_O, NaHCO_3_, H_2_O/EtOAc, rt, 2 h, 99% yield; b) 4-methylthiazole, Pd(OAc)_2_, KOAc, DMA, 90°C, 18 h, 82% yield; c) 4 M HCl in MeOH, rt, 3 h, 85% yield (Raina et al., 2016); c) 4 M HCl in dioxane, MeOH, rt, 12 h, 100% yield; d) i. Boc-Hyp-OH, HATU, DIPEA, DMF, 0°C to rt, 12 h; ii. 4 M HCl in dioxane, MeOH, rt, 12 h, 80% yield; e) i. Boc-Tle-OH, HATU, DIPEA, DMF, 0°C to rt, 12 h; ii. 4 M HCl in dioxane, MeOH, rt, 12 h, 80% yield (Hu et al., 2019); f) i. **116**, HATU, DIPEA, THF, rt, 2 h; ii. 4 M HCl in MeOH, rt, 3 h, 72% yield; g) POI ligand-linker-CO_2_H, HATU, DIPEA, DMF, 0°C to rt, 20 min, 32% yield for conjugate used (Raina et al., 2016); g) POI ligand-linker-CO_2_H, HATU, DIPEA, DMF, rt, 1 h, 40% yield for conjugate used (Hu et al., 2019).

In the course of design and optimization of VHL ligands, an introduction of an (*S*)-methyl group on the benzylic carbon atom has improved the binding affinity to VHL. Namely, the potency of the methyl-substituted ligand is three times better than of the non-substituted ligand **107** (Han et al., 2019). Synthesis of the key intermediate **120** is analogous to the synthetic route described in Scheme 23, using (*S*)-(-)-4-bromo-*α*-methylbenzylamine (**117**) as starting material. The left-hand side dipeptide moiety could be assembled either by convergent synthesis (Raina et al., 2016) or linear synthesis (Hu et al., 2019) to yield **122**, which was then ready for attaching carboxylic acid linker-POI ligand conjugates by a coupling reaction to give derivatives **123** (Scheme 24) (Raina et al., 2016; Hu et al., 2019).

#### B: Linkage via a Phenolic Group at the Phenylene Unit

**SCHEME 25:**
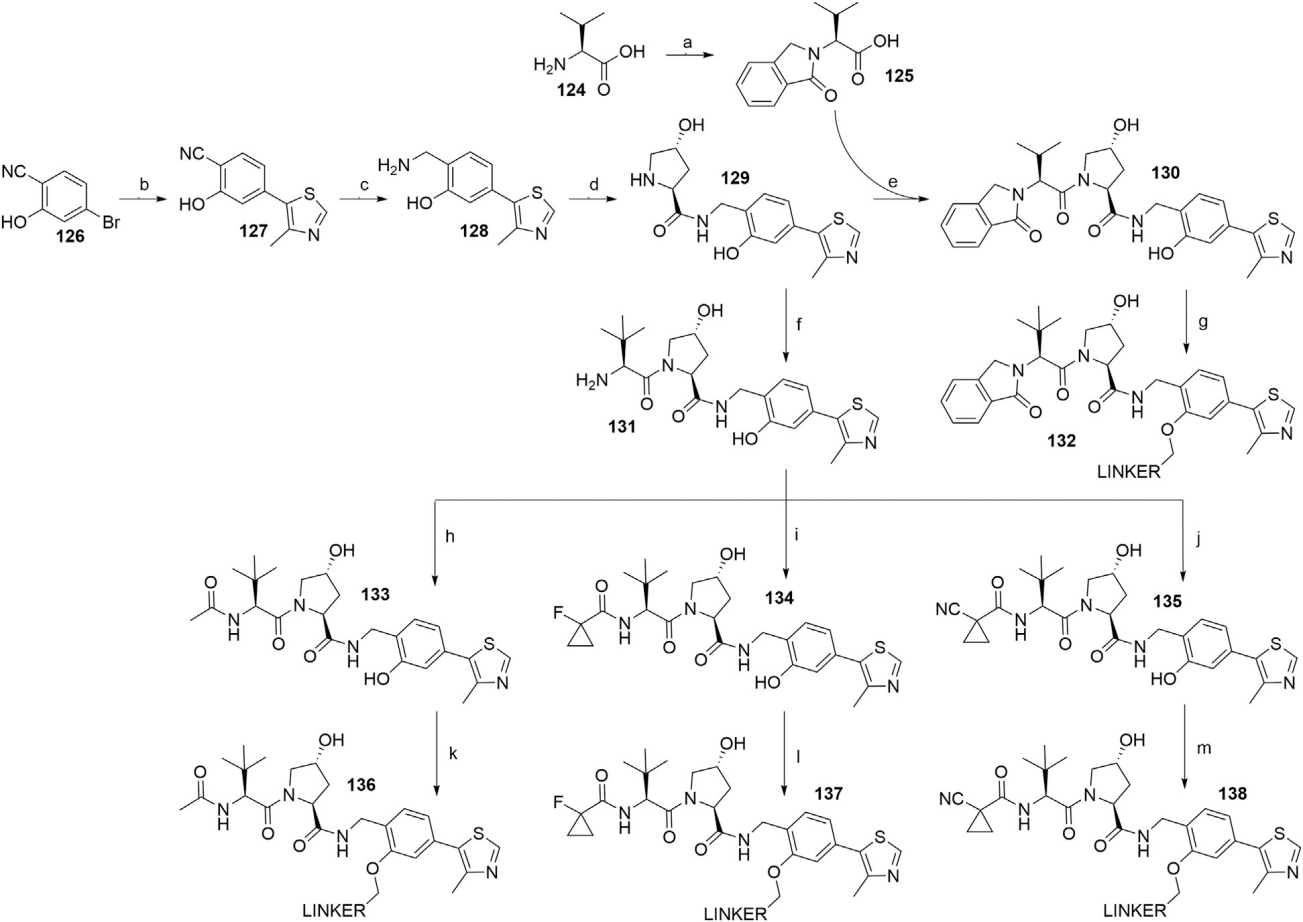
Syntheses of VHL ligands **130**, **133**, **134**, and **135**. Reagents and conditions: a) phthalaldehyde, MeCN, 90°C, 3.5 h, 83% yield; b) 4-methylthiazole, KOAc, Pd(OAc)_2_, DMA, 150°C, 15 h, 76% yield (Buckley et al., 2015); b) 4-methylthiazole, KOAc, Pd(OAc)_2_, DMA, 150°C, 20 h, 77% yield (Farnaby et al., 2019); c) LiAlH_4_, THF, 50°C, 22 h, 27% yield (Buckley et al., 2015); c) LiAlH_4_, THF, 0°C, 1 h to rt, overnight, 38% yield (Farnaby et al., 2019); d) i. Boc-Hyp-OH, HATU, HOAt, DIPEA, DMF, rt, 1 h; ii. 4 M HCl in dioxane, CH_2_Cl_2_, rt, 2 h, 55% yield (Maniaci et al., 2017); d) i. Boc-Hyp-OH, HATU, DIPEA, DMF, 4°C to rt, 2.5 h; ii. 4 M HCl in dioxane, CH_2_Cl_2_, MeOH, rt, 16 h, 52% yield (Buckley et al., 2015); d) i. Boc-Hyp-OH, HATU, DIPEA, DMF, rt, overnight; ii. 4 M HCl in dioxane, CH_2_Cl_2_, rt, overnight, 54% yield (Farnaby et al., 2019); e) HATU, DIPEA, DMF, rt, 22 h, 24% yield (Buckley et al., 2015); f) i. Boc-Tle-OH, HATU, HOAt, DIPEA, DMF, rt, 1 h; ii. 4 M HCl in dioxane, CH_2_Cl_2_, rt, 2 h, 55% yield (Maniaci et al., 2017); f) i. Boc-Tle-OH, HATU, DIPEA, DMF, rt, overnight; ii. 4 M HCl in dioxane, CH_2_Cl_2_, rt, overnight, 55% yield (Farnaby et al., 2019); g) linker-OMs, K_2_CO_3_, DMF, 70°C, overnight, 37–60% yield for linkers used (Buckley et al., 2015); g) linker-OMs, Cs_2_O_3_, rt, 18 h, then 60°C, 3 h, 49–68% yield for linkers used (Steinebach et al., 2020a); h) acetylimidazole, DIPEA, DMF, rt, 48 h, 78% yield (Maniaci et al., 2017); i) 1-fluorocyclopropanecarboxylic acid, Et_3_N, DMF, rt, overnight, 76% yield (Farnaby et al., 2019); i) 1-fluorocyclopropanecarboxylic acid, HATU, HOAt, DIPEA, DMF, rt, 2 h, 57% yield (Zoppi et al., 2019); j) 1-cyanocyclopropanecarboxylic acid, HATU, HOAt, DIPEA, DMF, rt, 1 h, 55% yield; k) linker-OMs, K_2_CO_3_, DMF, 70°C, overnight, 33% yield for linker used (Maniaci et al., 2017); l) linker-OMs, K_2_CO_3_, DMF, 75°C, overnight, 85% yield for linker used (Farnaby et al., 2019); l) linker-OMs, K_2_CO_3_, DMF, 70°C, overnight, 35–79% yield for linkers used (Zoppi et al., 2019); l) linker-OMs, Cs_2_O_3_, rt, 18 h, then 60°C, 3 h, 52% yield for linker used (Steinebach et al., 2020a); m) linker-OMs, K_2_CO_3_, DMF, 70°C, overnight, 33% yield for linker used (Maniaci et al., 2017); m) linker-OMs, Cs_2_CO_3_, rt, 18 h, then 60°C, 3 h, 52% yield for linker used (Steinebach et al., 2020a).

The recent literature on VHL-recruiting PROTACs confirmed that a phenolic linkage point is well-tolerated (Farnaby et al., 2019), with the left-hand dipeptide part of the molecule permitting the attachment of various substituents. The structure-activity relationship studies resulted in ligands **130** (Buckley et al., 2015), **133** (Maniaci et al., 2017), **134** (Farnaby et al., 2019; Zoppi et al., 2019), and **135** (Maniaci et al., 2017) with high VHL binding affinity (Scheme 25). To synthesize these compounds, the starting 4-bromo-2-hydroxybenzonitrile (**126**) was transformed into **127** through a Heck reaction, where prolonging the reaction time from 15 h (Buckley et al., 2015) to 20 h (Farnaby et al., 2019) only had a minor effect on the yield. The key intermediate **129** was formed through a reduction to amine **128** using LiAlH_4_ with low reported yields of 27% (Buckley et al., 2015) and 38% (Farnaby et al., 2019), followed by amide bond formation with Boc-l-hydroxyproline and subsequent Boc deprotection (Scheme 25, steps b–d) (Buckley et al., 2015; Farnaby et al., 2019). Coupling of **129** with **125** formed VHL ligand **130**, which allowed for the attachment of linkers with a terminal mesylate group to obtain conjugates **132** in a 37–68% yield for linkers used (Buckley et al., 2015; Steinebach et al., 2020a). Alternatively, **129** was first reacted with Boc-l-*tert*-leucine and then Boc-deprotected to yield **131**, which was then derivatized into VHL ligands **133** using acetylimidazole (Maniaci et al., 2017), **134** using 1-fluorocyclopropanecarboxylic acid (Farnaby et al., 2019; Zoppi et al., 2019), and **135** using 1-cyanocyclopropanecarboxylic acid (Maniaci et al., 2017). Each of those VHL ligands then had mesylate linkers attached to the phenol under standard conditions, i.e. K_2_CO_3_, DMF, 70°C (Maniaci et al., 2017; Farnaby et al., 2019; Zoppi et al., 2019). Alternatively, Cs_2_O_3_ can be used as a base in place of K_2_CO_3_ (Steinebach et al., 2020a).

**SCHEME 26:**
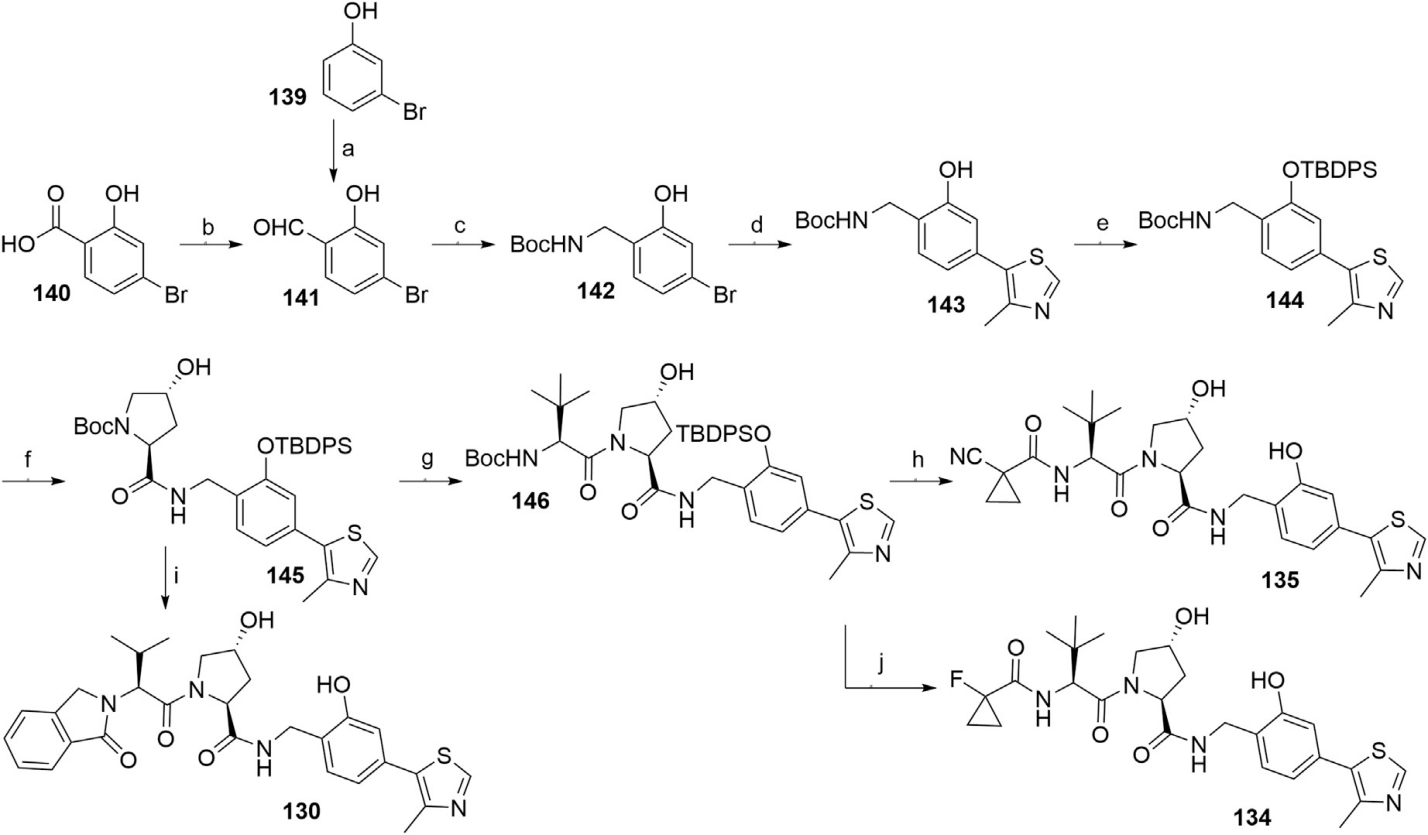
Synthesis of VHL ligands **130**, **134**, and **135**. Reagents and conditions: a) paraformaldehyde, Et_3_N, MgCl_2_, THF, reflux, 6 h, 32% yield; b) i. *N*,*O*-dimethylhydroxylamine, EDC, Et_3_N, CH_2_Cl_2_, rt, 16 h, 78% yield; ii. LiAlH_4_, THF, 0°C, 30 min, 53% yield; c) *tert*-butyl carbamate, Et_3_SiH, TFA, CH_2_Cl_2_, MeCN, rt, 18 h, 94% yield; d) 4-methylthiazole, KOAc, Pd(OAc)_2_, DMA, 130°C, 4 h, 60% yield; e) TBDPSCl, imidazole, DMF, rt, 18 h, 92% yield; f) i. TFA, CH_2_Cl_2_, rt, 2 h; ii. Boc-Hyp-OH, HATU, DIPEA, DMF, rt, 18 h, 75% yield over two steps; g) i. TFA, CH_2_Cl_2_, rt, 2 h; ii. Boc-Tle-OH, HATU, DIPEA, DMF, rt, 18 h, 60% yield over two steps; h) i. TFA, CH_2_Cl_2_, rt, 2 h; ii. 1-cyanocyclopropanecarboxylic acid, HATU, DIPEA, DMF, rt, 18 h, 77% yield over two steps; iii. TBAF, THF, 0°C to rt, 18 h, 98% yield; i) i. TFA, CH_2_Cl_2_, rt, 2 h; ii. **125**, HATU, DIPEA, DMF, rt, 18 h, 56% yield over two steps; iii. TBAF, THF, 0°C to rt, 18 h; j) i. TFA, CH_2_Cl_2_, rt, 2 h; ii. 1-fluorocyclopropanecarboxylic acid, HATU, DIPEA; DMF, rt, 18 h, 69% yield over two steps; iii. TBAF, THF, 0°C to rt, 18 h (Steinebach et al., 2020b).

An alternative synthetic route used 4-bromo-2-hydroxybenzaldehyde (**141**) in place of 4-bromo-2-hydroxybenzonitrile (**126**, Scheme 25) (Steinebach et al., 2020a). Starting material **141** is easily accessible by *ortho*-formylating 3-bromophenol (**139**) or by transforming 4-bromosalicyclic acid (**140**) into a Weinreb amide and its subsequent reduction (Scheme 26). Compound **143** was obtained through reductive amination of **141** with *tert*-butyl carbamate under mild conditions and Heck coupling in a higher yield compared to the analogous synthesis of compound **128** (Scheme 25, step c). The phenol group of **143** was then protected to prevent the formation of acylated by-products in the following coupling reactions (Scheme 26, steps f–j). The key intermediate **145** was generated from **144** and Boc-l-hydroxyproline and then coupled with **125** and deprotected to give VHL ligand **130**. Alternatively, forming an amide bond between **145** and Boc-l-*tert*-leucine yielded compound **146**, which was then Boc-deprotected and derivatized into VHL ligands **134** and **135** (Steinebach et al., 2020b).

#### C: Attachment via a Thioether at the Left-Hand Side Amino Acid

**SCHEME 27:**
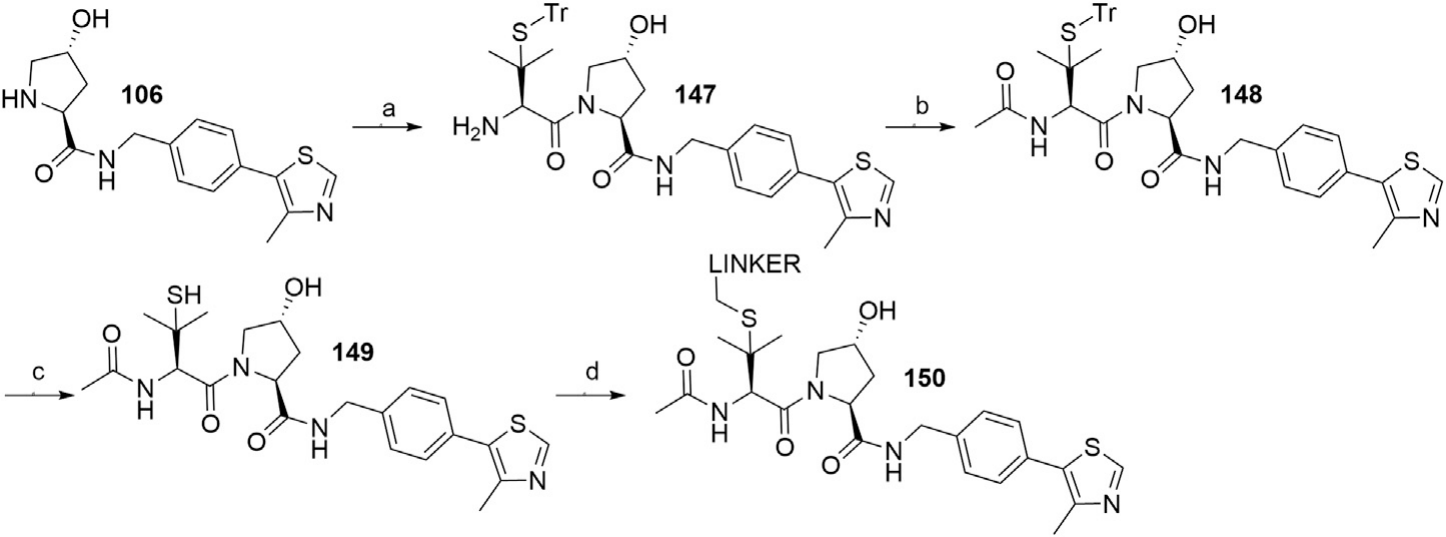
Synthesis of derivatives **150** with a thioether bond. Reagents and conditions: a) i. Fmoc-(*S*)-trityl-l-penicillamine, HATU, HOAt, DIPEA, DMF, rt, 2 h; ii. piperidine, CH_2_Cl_2_, rt, 1 h, 75% yield; b) Ac_2_O, Et_3_N, CH_2_Cl_2_, rt, 2 h, 98% yield; c) TIPS, TFA, CH_2_Cl_2_, rt, 2 h, 79% yield; d) linker-OMs/-OTs/Br, DBU, DMF, 0°C to rt, 1–3 h, 70–82% yield for linkers used (Gadd et al., 2017).

The side chain of *tert*-leucine group on VHL ligand’s left-hand side represents a possible linker attachment point, so it was replaced with trityl-protected penicillamine to synthesize compound **147**. After treating **147** with acetic anhydride to afford **148**, and subsequently removing the trityl group, the thiol-containing fragment **149** was obtained, to which mesylate, tosylate or bromo linkers were attached to form thioether conjugates **150** (Scheme 27) (Gadd et al., 2017).

#### D: Connection via the Benzylic Position

**SCHEME 28:**
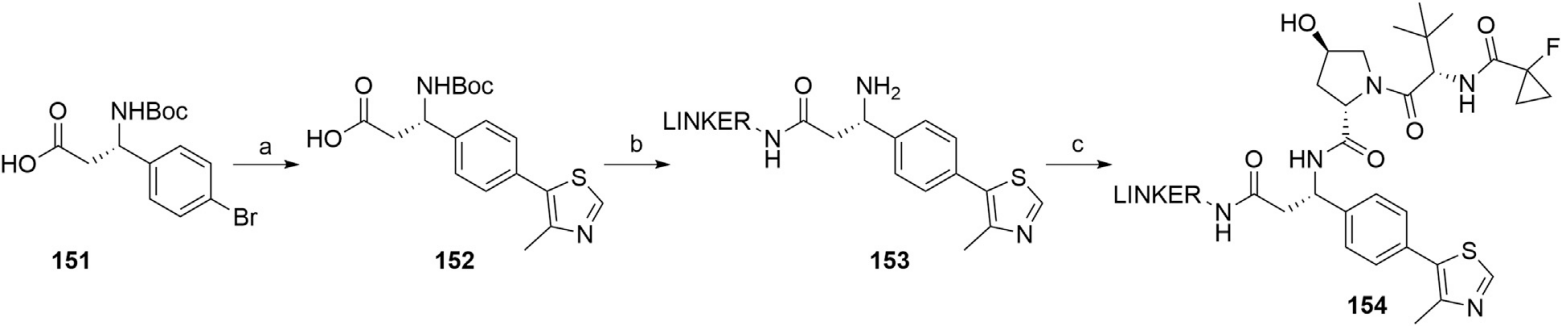
Synthesis of conjugates **154** connected with the linker *via* the benzylic position. Reagents and conditions: a) 4-methylthiazole, Pd(OAc)_2_, KOAc, Et_3_N, DMF, 80°C, 4 h, 80% yield; b) i. POI ligand-linker-NH_2_, HATU, DIPEA, DMF, rt, 30 min; ii. TFA, CH_2_Cl_2_, 80% yield for 2 steps; c) (2*S*,4*R*)-1-((*S*)-2-(1-fluorocyclopropane-1-carboxamido)-3,3-dimethylbutanoyl)-4-hydroxypyrrolidine-2-carboxylic acid, HATU, DIPEA, DMF, rt, 30 min (Han et al., 2019, 69).

Based on analyses of co-crystal structures of VHL ligand **122** (Scheme 24) in the active site of the enzyme, the (*S*)-methyl group of the VHL ligand was found to be exposed to the solvent and therefore represents a possible liker attachment point for the design of PROTACs. 4-Methylthiazole was coupled with commercially available **151** to yield **152**, to which a desired linker-POI ligand conjugate was attached *via* an amide bond. Boc deprotection afforded compound **153**, and the left-hand side dipeptide part was attached to form conjugates **154** (Scheme 28) (Han et al., 2019).

#### von Hippel–Lindau Photacs

**SCHEME 29:**
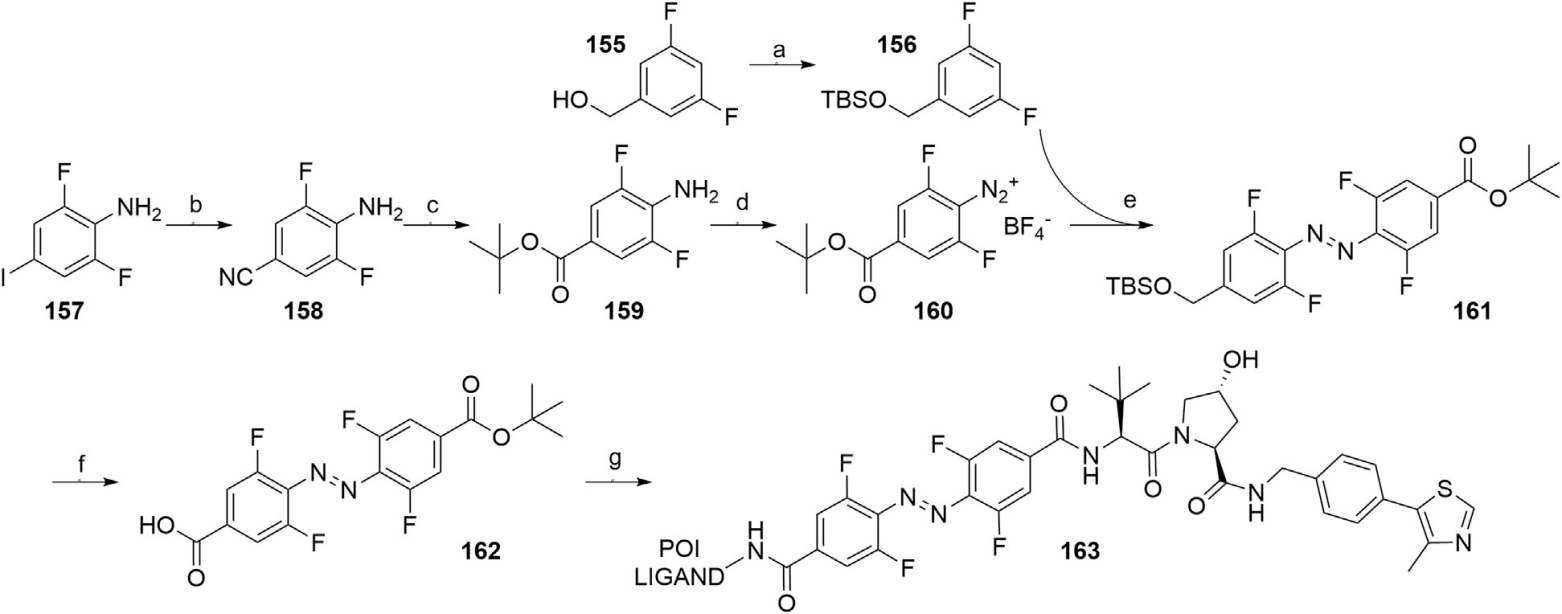
Synthesis of VHL-targeting PHOTAC **163**. Reagents and conditions: a) imidazole, TBSCl, CH_2_Cl_2_, rt, 16 h, 98% yield; b) CuCN, NMP, 180°C, 7 h, 91% yield; c) i. 1 M NaOH (aq), reflux, 1 h, 89% yield; ii. Oxalyl chloride, DMF, rt, 30 min, then *t*-BuOK, THF, 0°C; iii. *N*, *N′*-dimethylethane-1,2-diamine, EtOH, 110°C, 12 h, 50% yield over two steps; d) NOBF_4_, EtOAc, 0°C, 1 h, 72% yield; e) *t*-BuLi, THF, –78°C to –50°C, 1 h, then **160**, –78°C to rt, 1 h, 76% yield; f) i. TBAF, THF, 0°C, 15 min, 76% yield; ii. TEMPO, NaClO, NaClO_2_, MeCN/pH 6.8 phosphate buffer, rt, 3 h, quant.; g) i. **107**, HATU, DIPEA, DMF, rt, 2 h, 88% yield; ii. TFA, CH_2_Cl_2_, rt, 1 h, quant.; iii. POI ligand-NH_2_, HATU, DUPEA, DMF, rt, 2 h, 51% yield for POI ligand used (Pfaff et al., 2019).

An azobenzene handle was employed in place of a standard linker, which allowed for photoinduced switching between the inactive *cis* and active *trans* configuration of the VHL-targeting PHOTAC (Pfaff et al., 2019). Intermediate **159** was generated from 2,6-difluoro-4-iodoaniline (**157**) (Scheme 29, steps b-c) and then treated with nitrosonium tetrafluoroborate to afford diazonium tetrafluoroborate **160**. TBS-protection of (3,5-difluorophenyl)methanol (**155**) led to compound **156**, which was treated with *tert*-butyllithium and combined with **160**, giving **161**. Following the TBS-deprotection and oxidation, **162** was coupled with VHL ligand **107**, Boc-deprotected and additionally coupled with POI ligand-amine, to generate the finished PHOTAC compound **163** (Pfaff et al., 2019).

#### Caged von Hippel–Lindau Ligands

**SCHEME 30:**
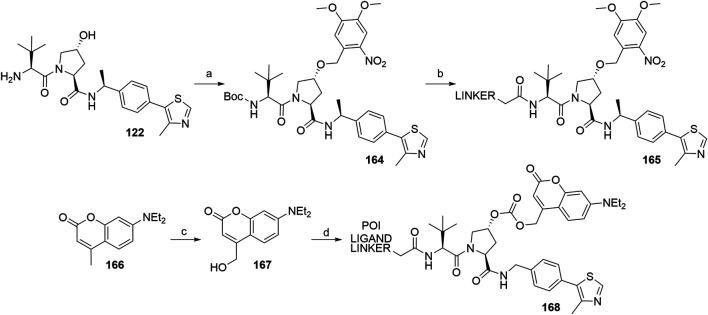
Syntheses of caged VHL degraders **165** and **168**. Reagents and conditions: a) i. (Boc)_2_O, Et_3_N, CH_2_Cl_2_, rt, 16 h, 77% yield; ii. 4,5-dimethoxy-2-nitrobenzyl bromide, TBAI, 50% NaOH (aq), CH_2_Cl_2_, rt, 2 h, 53% yield; b) i. HCl in dioxane, rt, 4 h, quant.; ii. linker-CO_2_H, HATU, DIPEA, CH_2_Cl_2_, rt, 16 h, 41% yield for linker used (Kounde et al., 2020); c) i. SeO_2_, dioxane, reflux, 16 h; ii. NaBH_4_, EtOH, 0°C to rt, 4 h; d) i. diphosgene, CH_2_Cl_2_, 0°C to rt, 24 h; ii. POI ligand-linker-VHL ligand **A1**, DMAP, DIPEA, CH_2_Cl_2_, 0°C to rt, 16 h, 59% yield for conjugate used (Naro et al., 2020).

The concept of caged E3 ligase ligands was used for the controlled degradation of BRD4 by incorporating a photocleavable 4,5-dimethoxy-2-nitro-benzyl group (DMNB), bound to the hydroxyproline core of the VHL ligand, connected *via* a linker to pan-bromodomain inhibitor JQ1. Following irradiation with a wavelength of 365 nm, the PROTAC could be uncaged, which triggered the degradation of BRD4. To prepare a caged PROTAC, the VHL ligand **122** was first *N*-Boc protected, followed by the functionalization of hydroxyl group by forming an ether bond with the DMNB group using phase transfer catalysis to yield **164**. After Boc deprotection, a carboxylic acid linker was introduced *via* an amide bond to form **165** (Scheme 30) (Kounde et al., 2020). Additionally, the concept was also utilized for the degradation of estrogen related receptor α, where a diethylamino coumarin (DEACM) group was installed at the hydroxyl group of the VHL ligand *via* a carbonate linkage. Irradiation with a wavelength of 360 nm causes the photolysis and subsequent decaging of the VHL ligand, thus activating the degrader. Compound **167** was obtained from starting material **166** over two steps and then converted to a chloroformate before being attached to a POI ligand-linker-VHL ligand conjugate, forming the final caged PROTAC **168** (Naro et al., 2020).

#### Statistical Overview of Utilized von Hippel–Lindau Ligands

**FIGURE 5:**
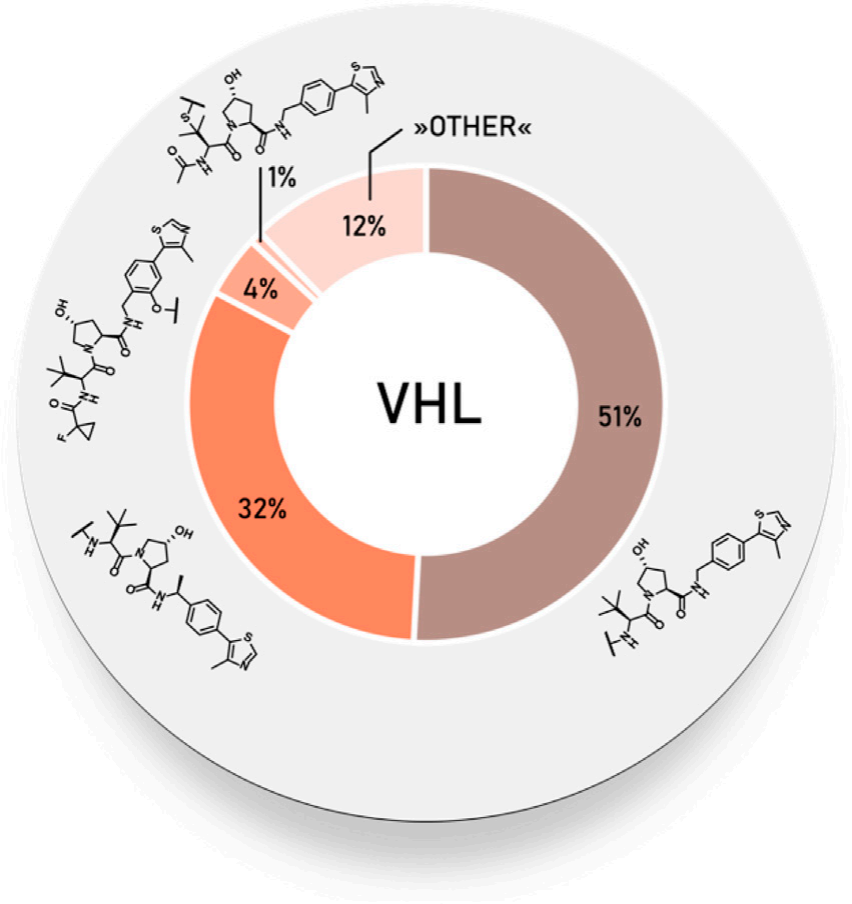
Frequency of VHL ligands used in PROTAC compounds.

Using data extracted from PROTAC-DB (Weng et al., 2021) (http://cadd.zju.edu.cn/protacdb/, as of the February 26, 2021), a statistical overview was done to determine the frequency of various VHL ligands and linker attachment options used in PROTAC compounds. The vast majority of PROTACs incorporated VHL ligand 1, while the (*S*)-methyl group-containing ligand occurred in about a third of degraders. Linkage *via* a phenolic group at benzene ring was less commonly utilized, at about 4%. In comparison, attachment *via* a thioether at the left-hand side amino acid could be found in only around 1% of PROTACs (Figure 5).

### Inhibitor of Apoptosis Proteins

The family of inhibitor of apoptosis proteins (IAP) includes antiapoptotic proteins, which are commonly overexpressed in some cancer cells and promote their survival, as well as the survival of neuronal cells (Fulda and Vucic, 2012; Ohoka et al., 2017b). All IAP proteins contain one to three baculoviral IAP repeat (BIR) domains that interact with their binding proteins, while some of them [cellular IAP1(c-IAP1), c-IAP2, X chromosome-linked IAP (XIAP), and melanoma IAP (ML-IAP)] also contain a RING finger domain, which provides an E3 ubiquitin ligase activity (Cohen and Tcherpakov, 2010; Itoh et al., 2010; Fulda and Vucic, 2012; Ohoka et al., 2017b). Because of their involvement in multiple malignancies, inhibitors of these proteins represent an attractive strategy for tumor therapy, and many potent peptidomimetic antagonists have been developed based on the endogenous inhibitory IAP protein second mitochondria-derived activator of caspase/direct inhibitor of apoptosis-binding protein with low pI (Smac/DIABLO) (Ohoka et al., 2017b). Interaction between IAP antagonists and their targets results in the autoubiquitylation and proteasomal degradation of cIAP1 (Varfolomeev et al., 2007; Vince et al., 2007).

**FIGURE 6:**
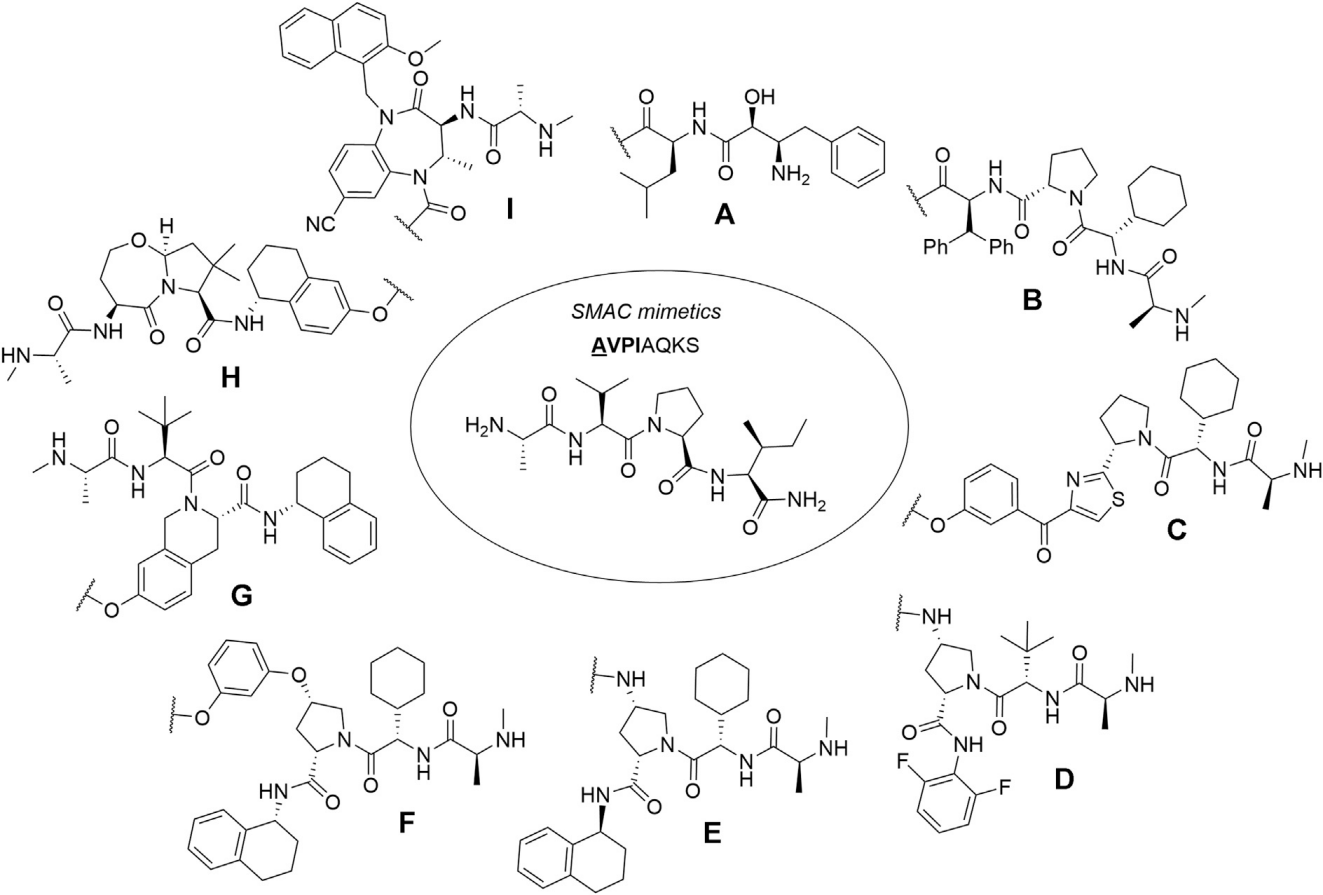
IAP ligands. IAP ligand **(A)** (bestatin), IAP ligand **(B)** (MV1 derivative), IAP ligand **(C)** (LCL**-**161 derivative), IAP ligand **(D)** (Cmpd37 derivative), IAP ligand **(E)** (A410099 derivative), IAP ligand **(F)**, IAP ligand **(G)**, IAP ligand **(H)** (SBP-0636457 derivative), IAP ligand **(I)**.

First hybrid molecules that utilized c-IAP1 for its E3 ubiquitin ligase activity have been described in 2010 and those compounds induced the degradation of cellular retinoic acid-binding proteins (Itoh et al., 2010). Alternatively to PROTACs, for IAP-recruiting degraders, a different terminology is also used, i.e., specific and nongenetic IAP-dependent protein erasers (SNIPERs) (Sun X. et al., 2019). Methyl bestatin derivative (Figure 6, **A**) was used as the c-IAP1-binding ligand for the early reported degraders recruiting this E3 ligase (Itoh et al., 2010; Okuhira et al., 2011; Okuhira et al., 2013; Okuhira et al., 2016; Demizu et al., 2012; Ohoka et al., 2014). Future development of high-affinity IAP ligands and their incorporation into bifunctional molecules improved the efficiency of SNIPERs in comparison with early bestatin-based compounds (Ohoka et al., 2017b; Okuhira et al., 2018; Naito et al., 2019). Structures of IAP ligands utilized in chimeric molecules are presented in Figure 6.

#### Inhibitor of Apoptosis Proteins Ligand A: Bestatin

##### Aromatic α-Aminoaldehydes as a Starting Material for Bestatin Synthesis

**SCHEME 31:**
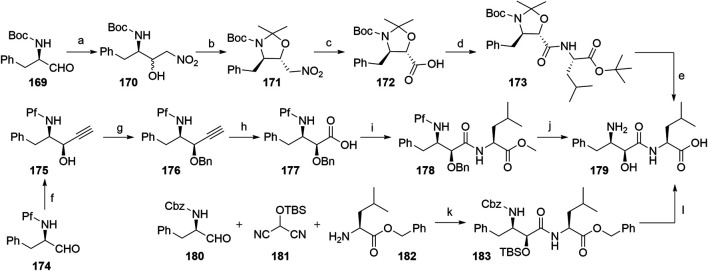
Syntheses of bestatin (**179**). Reagents and conditions: a) nitromethane, NaH, 15-crown-5, EtO_2_, hexane, 0°C to rt, 22 h, 64% yield; b) 2,2-dimethoxypropane, BF_3_×OEt_2_, 0°C, 1 h, then rt, overnight, 54% yield; c) KOH, KMnO_4_, Na_2_HPO_4_, MeOH, H_2_O, 0°C, 2 h, quant.; d) isobutyl chloroformate, NMP, THF, 0°C to –12°C, 30 min, then l-leucine tert-butyl ester, NMP, DMF, –12°C, 90 min, 69% yield; e) TFA, H_2_O, 0°C, 2.5 h, quant. (Shang et al., 2018); f) ethynylmagnesium bromide, THF, –40°C, 10 min, 96% yield; g) BnBr, NaH, Bu_4_NI, THF, 0°C, 97% yield; h) KMnO_4_, AcOH, H_2_O, pentane, 87% yield; i) l-leucine methyl ester, DCC, HOBt, TsOH, Et_3_N, THF, 0°C, 91% yield; j) i. LiOH, THF, H_2_O, 0°C, 95% yield; ii. Pd/C, H_2_, MeOH, 50°C, 93% yield (Lee et al., 2003); k) 4-pyrrolidinopyridine, Et_2_O, 0°C, 5 h, diastereomeric mixture in 80% yield, 79:21 ratio; l) i. Bu_4_NF, THF, 0°C, 20 min, 96% yield; ii. Pd/C, H_2_, MeOH, rt, 2 h, quant (Nemoto et al., 2000).

Several authors proposed different synthetic routes for the synthesis of bestatin, utilizing aromatic α-aminoaldehydes as starting compounds (Scheme 31). For example, compound **169** was treated with nitromethane to afford a diastereomeric mixture of nitroaldols **170**, which were then converted into a mixture of dimethyl oxazolidines, out of which the desired compound **171** was separated by silica gel column chromatography in a 54% yield. Compound **172** was obtained by a Nef reaction and then coupled with l-leucine *tert*-butyl ester to yield **173**. Finally, Boc cleavage using TFA afforded bestatin (**179**) in an overall yield of 24% (Scheme 30, steps a–e) (Shang et al., 2018). An alternative route started from aldehyde **174**, which was converted to *syn*-aminoalcohol **175** in a 96% yield and a 9.5:1 *syn*/*anti* stereochemic ratio. The hydroxyl group was then protected with a Bn group to obtain **176**, followed by terminal alkyne oxidation to carboxylic acid **177**. Coupling reaction with l-leucine methyl ester afforded compound **178**, and removal of the protecting groups led to the desired product **179** with an overall yield of 59% (Scheme 31, steps f–j) (Lee et al., 2003). Furthermore, a one-pot method was described, in which starting materials **180**, **181**, and **182** were joined into **183** with 63% yield. Following the deprotection, bestatin (**179**) was obtained in an overall yield of 60% (Scheme 31, steps k–l) (Nemoto et al., 2000).

##### Alternative Routes for the Synthesis of Bestatin

**SCHEME 32:**
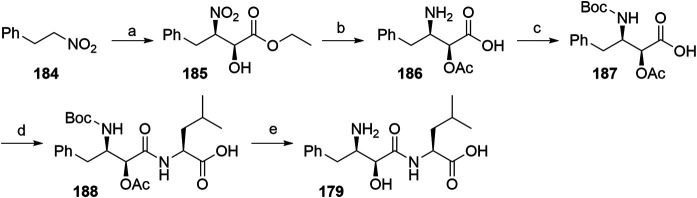
The synthesis of bestatin (**179**) (Gogoi et al., 2005). Reagents and conditions: a) ethyl glyoxalate, La-(*R*)-BINOL, THF, –50°C, 81% yield, 93% ee; b) i. acetylation, 94% yield (no detailed reagents given); ii. Pd/C, NaBH_4_, H_2_, MeOH, 60%, 93% ee; c) (Boc)_2_O, NaHCO_3_, H_2_O, EtOAc, 92% yield; d) i. l-leucine benzyl ester, *N*-ethylmorpholine, isobutyl chloroformate, THF,–10°C; ii. Pd/C, H _2_, MeOH, 77% yield over two steps; e) i. K_2_CO_3_, MeOH; ii. TFA, 73% yield over two steps (Gogoi et al., 2005).

One route included the treatment of (2-nitroethyl)benzene (**184**) with ethyl glyoxalate in Shibasaki’s asymmetric Henry reaction, which was catalyzed by an optically active lanthanum-(*R*)-binaphthol complex. Compound **185** was then *O*-acetylated before reducing the nitro group to yield **186**. Following *N*-Boc protection, **187** was coupled with l-leucine benzyl ester, followed by immediate deprotection of the terminal carboxylic moiety. Both protecting groups of **188** were removed to give bestatin (**179**) in an overall yield of 26% (Scheme 32) (Gogoi et al., 2005).

**SCHEME 33:**
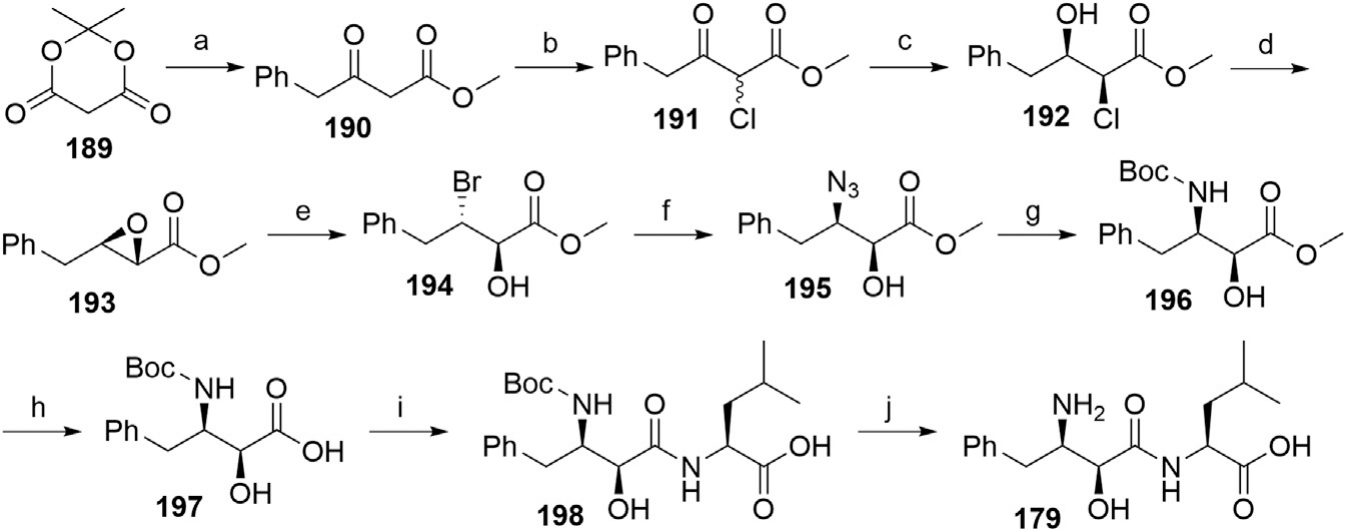
Alternative route for the syntheses of bestatin (**179**). Reagents and conditions: a) phenylacetyl chloride, pyridine, CH_2_Cl_2_, 0°C, 92% yield; b) SO_2_Cl_2_, 0°C, overnight, 88% yield; c) Ru_2_Cl_4_[R-BINAP]_2_(NEt_3_)H_2_, MeOH, rt, 20 h, 95% yield, 63:37 *syn*/*anti*; d) MeONa, MeOH, 0°C to rt, 2 h, 75% yield (Sayo et al., 1996). e) MgBr_2_, Et_2_O, rt, 2 h, 92% yield; f) NaN_3_, DMSO, 40°C, 6 h, 73% yield; g) i. Pd/C, H_2_, EtOAc; ii. (Boc)_2_O, EtOAc, rt, 5 h, 95% yield; h) Na_2_CO_3_, MeOH, H_2_O, rt, 12 h, 79% yield; i) i. l-leucine benzyl ester tosylate, EDC, HOBt, DIPEA, DMF, CH_2_Cl_2_, rt, 12 h, 87% yield; ii. Pd/C, H_2_, MeOH, rt, 95% yield; j) TFA, CH_2_Cl_2_, rt, 12 h, 85% yield (Righi et al., 2003).

A procedure partly derived from the patent literature started with the treatment of the Meldrum’s acid 189 with phenylacetyl chloride to yield 190, which was then chlorinated using sulfuryl chloride to form 191. The following asymmetric hydrogenation using a ruthenium-phosphine complex afforded compound 192, which was then subjected to epoxidation to obtain 193 (Sayo et al., 1996). Compound 194 was synthesized through an MgBr2-mediated ring opening of 193. Treatment with NaN_3_ afforded the azide derivative 195, which was then hydrogenated and Boc-protected to give compound 196. Hydrolysis of the methyl ester allowed coupling of 197 with l-leucine, and deprotection of 198 yielded bestatin (179) (Scheme 33) (Righi et al., 2003).

##### Linker Attachment to Bestatin

**SCHEME 34:**
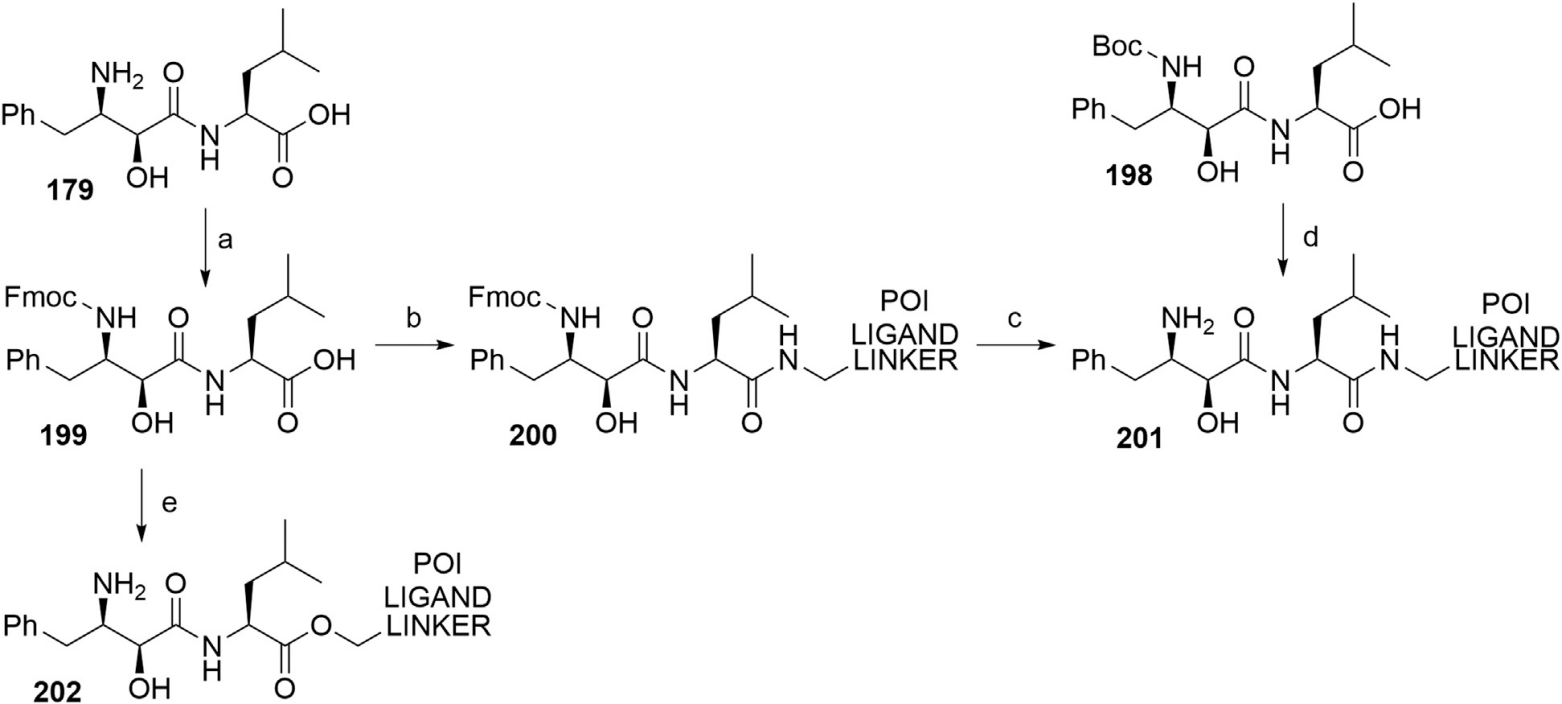
Linker attachment *via* an amide or an ester bond. Reagents and conditions: a) Fmoc-Cl, K_2_CO_3_, THF, H_2_O, 0°C to rt, 24 h, 97% yield (Itoh et al., 2010); a) Fmoc-Cl, K_2_CO_3_, THF, H_2_O, rt, 81% yield (Ohoka et al., 2014); b) reagents, conditions, and yields are collected in Table 6; c) 2 M Me_2_NH in MeOH, rt, 84–89% yield for conjugates used (Ohoka et al., 2014); 2 M Me_2_NH in MeOH, rt, 6 h, 37% yield for conjugate used (Ohoka et al., 2017a); d) i. POI ligand-linker-NH_2_, EDC, HOBt, DIPEA, CH_2_Cl_2_, rt, 12 h, 77–86% yield for conjugates used; ii. 6M HCl (aq), THF, rt, 6 h, quant. (Okuhira et al., 2016); e) i. POI ligand-linker-OH, EDC, HOBt, DIPEA, CH_2_Cl_2_, 0°C to rt, 17 h, 36% yield for conjugate used; ii. DBU, dodecyl mercaptan, CH_2_Cl_2_, rt, 1 h (Itoh et al., 2010).

Bestatin (**179**) was incorporated into chimeric molecules either *via* an amide or ester bond, the latter being less frequently utilized. Prior to coupling with the selected POI ligand-amine linker conjugates, the amino group of bestatin was protected (Boc = **198** or Fmoc = **199**) (Itoh et al., 2010; Ohoka et al., 2014). A combination of EDC and HOBt for amide coupling yielded bifunctional compounds **200** in a yield spanning from 81 to 89% for various conjugates used (Itoh et al., 2011; Ohoka et al., 2014; Okuhira et al., 2017a). Alternatively, the ester bond was formed under similar conditions, using a POI ligand-linker hydroxyl conjugate. However, the reported yield was much lower (36%) (Itoh et al., 2010). Using the appropriate deprotection procedure, final SNIPER compounds **201** and **202** were synthesized (Scheme 34) (Itoh et al., 2010; Okuhira et al., 2016).

**TABLE 6:** Reagents, conditions, and yields for the conversion of **199** to **200** (Scheme 34, step b).

Paper	Reagents and conditions	Yield
Ohoka et al. (2014)	POI ligand-linker-NH_2_, EDC, HOBt, THF, rt	86–87% yield for conjugates used
Itoh et al. (2011)	POI ligand-linker-NH_2_, EDC, HOBt, CH_2_Cl_2_, 0°C to rt, 17 h	89% yield for conjugates used
Shibata et al. (2017b)	POI ligand-linker-NH_2_, EDC, HOBt, THF, rt, overnight	81% yield for conjugates used

#### Inhibitor of Apoptosis Proteins Ligand B: MV1 Derivative

**SCHEME 35:**
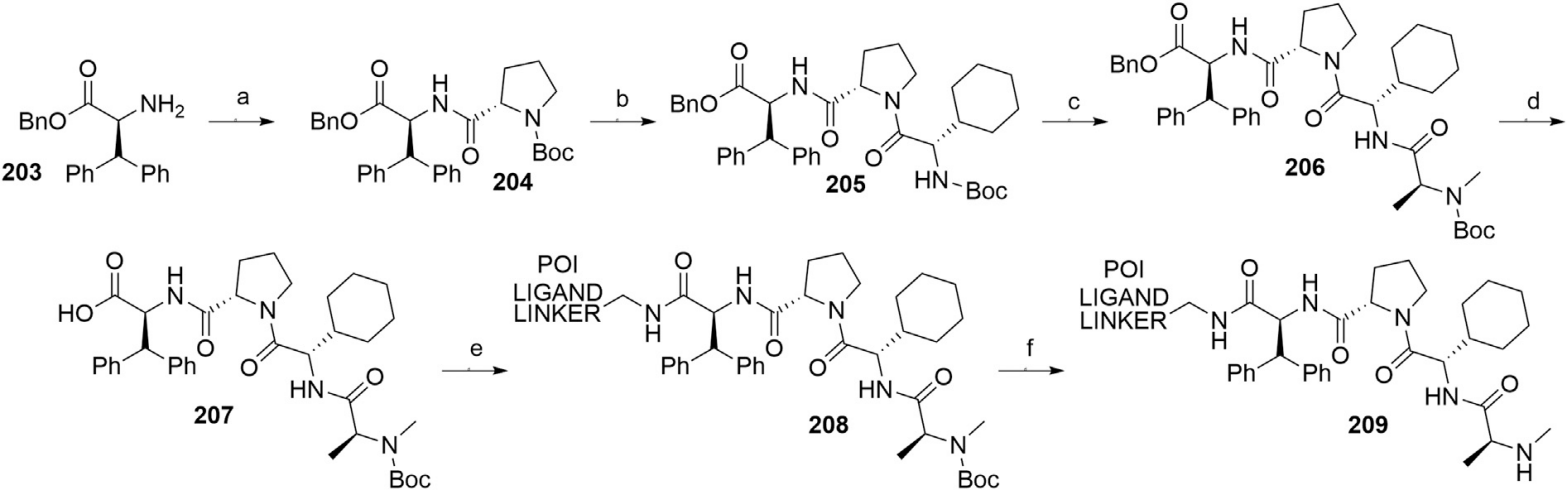
Synthesis of **IAP ligand B.** Reagents and conditions: a) *N*-Boc-l-proline, EDC, HOBt, DIPEA, DMF, rt, 22 h, 84% yield (Itoh et al., 2012); a) *N*-Boc-l-proline, EDC, HOBt, DIPEA, DMF, rt, 3 h, 95% yield (Shibata et al., 2017b); b) i. HCl, dioxane, rt, 4.5 h; ii. Boc-l-cyclohexylglycine, EDC, HOBt, DIPEA, DMF, rt, 13 h, 86% yield (Itoh et al., 2012); b) i. 4 M HCl, CPME, rt, 3 h; ii. Boc-l-cyclohexylglycine, EDC, HOBt, DIPEA, DMF, overnight, 98% yield (Shibata et al., 2017b); c) i. HCl, dioxane, rt, 4.5 h; ii. Boc-*N*-methyl-l-alanine, EDC, HOBt, DIPEA, DMF, rt, 15 h, 80% yield (Itoh et al., 2012); c) i. 4 M HCl, CPME, rt, 3 h; ii. Boc-*N*-methyl-l-alanine, EDC, HOBt, DIPEA, DMF, rt, overnight, 94% yield (Shibata et al., 2017b); d) Pd/C, H_2_, dioxane, rt, 6.5 h, quant. (Itoh et al., 2012); d) Pd/C, H_2_, EtOH, rt, overnight, quant. (Shibata et al., 2017b); e) and f) reagents, conditions, and yields are collected in Table 7.

A similar stepwise peptide synthesis of **IAP ligand B** using starting biphenyl **203** was described (Itoh et al., 2012; Shibata et al., 2017b). Coupling with *N*-Boc-l-proline yielded compound **204**, the following coupling with Boc-l-cyclohexylglycine gave **205**, and finally adding Boc-*N*-methyl-l-alanine afforded **206**. Catalytic reduction cleaved the *O*-benzyl group and allowed for coupling of the resulting **207** (Boc-protected **MV1** derivative) with POI ligand-linker amine conjugates, giving bifunctional derivatives **208**, which were Boc-deprotected to obtain final SNIPER compounds **209** (Scheme 35) (Itoh et al., 2012; Ohoka et al., 2017b; Shibata et al., 2017b).

**TABLE 7:** Reagents, conditions, and yields for the conversion of **207** to **209** (Scheme 35, steps e–f).

Paper	Reagents and conditions	Yield
Ohoka et al. (2017b)	e) POI ligand-linker-NH_2_, EDC, HOBt, DIPEA, DMF, 0°C to rt, 16 h	80% yield for conjugate used
	f) 4 M HCl in MeOH, THF, rt, 1 h	17% yield for conjugate used
Ohoka et al. (2017b)	e) POI ligand-linker-NH_2_, EDC, HOBt, DIPEA, DMF, 0°C, 2 h	64–98% yield for conjugates used
	f) 4 M HCl in CPME, THF, rt, 3 h	70–90% yield for conjugates used
Shibata et al. (2017b)	e) POI ligand-linker-NH_2_, HATU, DIPEA, DMF, 0°C, 1 h	95% yield for conjugate used
	f) 4 M HCl, CPME, THF, rt, 3 h	84% yield for conjugate used
Itoh et al. (2012)	e) POI ligand-linker-NH_2_, EDC, HOBt, CH_2_Cl_2_, rt, 20 h	(no yield given)
	f) TFA, CH_2_Cl_2_, rt, 3.5 h	87% yield for conjugate used

**SCHEME 36:**
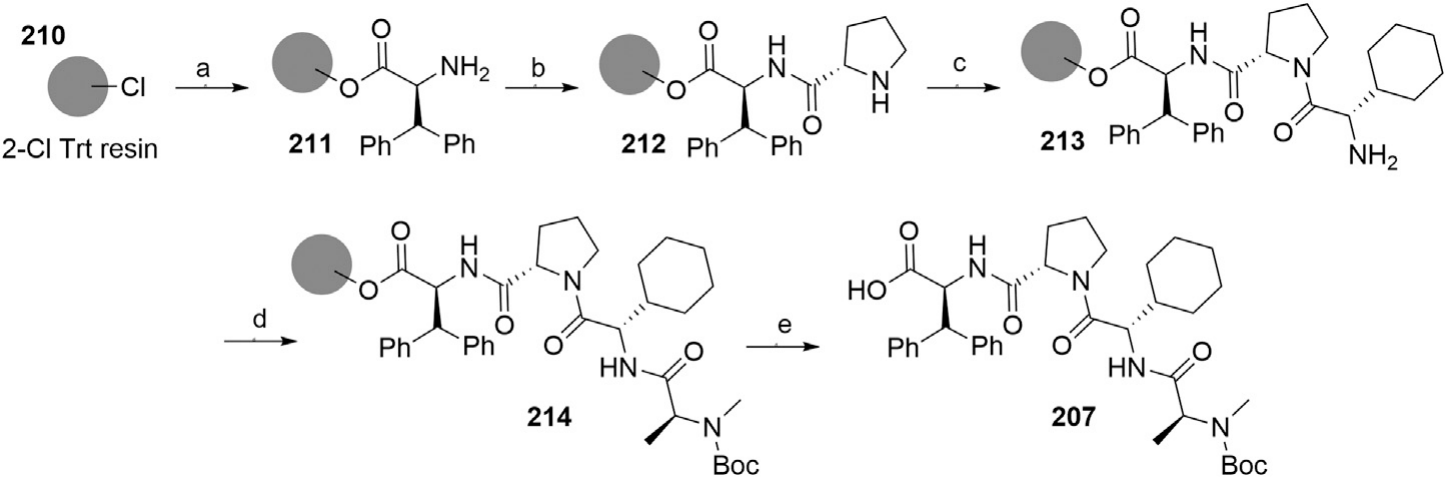
The synthesis of Boc-protected **IAP ligand B** (**207**) using 2-chlorotrityl chloride resin. Reagents and conditions: a) i. Fmoc-l-3,3-diphenylalanine, DIPEA, CH_2_Cl_2_, rt, 2 days; ii. 20% piperidine in DMF, rt, 10 min; b) i. Fmoc-l-Pro-OH, HCTU, HOBt, DIPEA, DMF, rt, 1.5 h; ii. 20% piperidine in DMF, rt, 10 min; c) Fmoc-l-cyclohexylglycin, HCTU, HOBt, DIPEA, DMF, rt, 1.5 h; ii. 20% piperidine in DMF, rt, 10 min; d) i. Fmoc-l-Ala-OH, HCTU, HOBt, DIPEA, DMF, rt, 1.5 h; ii. 20% piperidine in DMF, rt, 10 min; e) 1% TFA in CH_2_Cl_2_, rt, 5 min, 70% yield (Ohoka et al., 2017b).

A solid-phase peptide synthesis for an **IAP ligand B** derivative on a 2-chlorotrityl chloride resin was reported. The stepwise procedure was performed using HCTU, HOBt and DIPEA for coupling, followed by the addition of 20% piperidine in DMF to remove the Fmoc group after each step (Scheme 36, steps a–d). Finally, **214** was treated with 1% TFA in CH_2_Cl_2_ to remove the resin and to obtain **207** (Boc-protected **IAP ligand B**) (Ohoka et al., 2017b).

#### Inhibitor of Apoptosis Proteins Ligand C: LCL-161 Derivative

**SCHEME 37:**
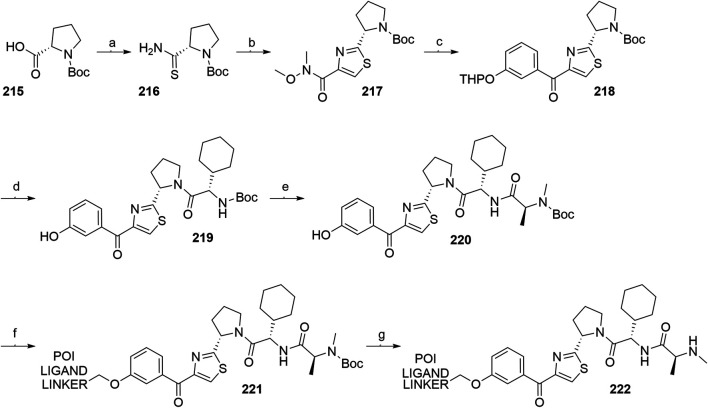
Synthesis of Boc-protected **IAP ligand C** (compound **220**) and derived degraders **222**. Reagents and conditions: a) i. HOBt·NH_3_, EDC, DMF, THF, 0°C to rt, 3 h, 96% yield; ii. Lawesson’s reagent, THF, 60°C, 2 h, 82% yield; b) i. ethyl bromopyruvate, EtOH, 60°C, 3 h; ii. (Boc)_2_O, NaHCO_3_, THF, H_2_O, rt, 3 h, 89% yield over two steps; iii. 1 M NaOH in MeOH, rt, overnight, 93%; iv. *N*,*O*-dimethylhydroxylamine hydrochloride, EDC, HOBt, DIPEA, DMF, rt, overnight, 97% yield (Ohoka et al., 2017b); c) i. 0.5 M 3-(2-tetrahydro-2*H*-pyranoxy)phenylmagnesium bromide in THF, –55 °C to –10 °C over 50 min, 99% yield; d) i. 2 M HCl in MeOH, rt, overnight; ii. Boc-l-cyclohexylglycine, EDC, HOBt, THF, 0°C, 1 h; iii. K_2_CO_3_, MeOH, 0°C, 2 h, 99% yield over three steps; e) i. 4 M HCl in CPME, MeOH, THF, rt, 4 h; ii. Boc-*N*-methyl-l-alanine, EDC, HOBt, DIPEA, DMF, rt, 2 h; iii. K_2_CO_3_, MeOH, H_2_O, 0 °C, 2 h, 42% yield over three steps; (Ohoka et al., 2017b; Shibata et al., 2017b) f) reagents, conditions, and yields are collected in Table 8; g) 4 M HCl in CPME, THF, rt, 4 h, 32–61% yield for conjugates used (Ohoka et al., 2017b); g) TFA, rt, 10 min, 70–90% yield for conjugates used (Shibata et al., 2017b); g) TFA, rt, 30 min, 50–68% yield for conjugates used (Shimokawa et al., 2017).

The procedure for the synthesis of **IAP ligand C (** Ohoka et al., 2017b; Shibata et al., 2017b) is shown in Scheme 37. The starting (*tert*-butoxycarbonyl)-l-proline (**215**) was converted into **216** over two steps before building the thiazole fragment to give **217**. Following the attachment of the 3-hydroxyphenyl building block to obtain **218**, the hydroxyl group was deprotected, and the right-hand side of the molecule was built by coupling with Boc-l-cyclohexylglycine to yield **219**, and Boc-*N*-methyl-l-alanine to produce the Boc-protected **IAP ligand C** (compound **220**) (Ohoka et al., 2017b; Shibata et al., 2017b). Tosylate-containing POI ligand-linker conjugates were attached to the phenol of **220** by heating in DMF or DMSO using K_2_CO_3_ as a base, with yields spanning between 62 and 81%, depending on the conjugate used (Ohoka et al., 2017b; Shibata et al., 2017b; Shibata et al., 2017a; Shimokawa et al., 2017). Alternatively, POI ligand-linker conjugates with a terminal hydroxyl group were attached under Mitsunobu reaction conditions (Shibata et al., 2017b). Final SNIPER molecules of type **222** were obtained by Boc-deprotection of bifunctional conjugates **221** (Scheme 37) (Ohoka et al., 2017b; Shibata et al., 2017b; Shimokawa et al., 2017).

**TABLE 8:** Reagents, conditions, and yields for the conversion of **220** to **221** (Scheme 37, step f).

Paper	Reagents and conditions	Yield
Ohoka et al. (2017b)	POI ligand-linker-OTs, K_2_CO_3_, DMF, 50°C, overnight –2 days	65–67% yield for conjugates used
Shibata et al. (2017b)	POI ligand-linker-OTs, K_2_CO_3_, DMF, 60°C, 4 h, then rt, overnight	67–81% yield for conjugates used
Shibata et al. (2017b)	POI ligand-linker-OH, PPh_3_, 40% DEAD in toluene, THF, 0°C to rt, 5 h	Quant. for conjugate used
Shibata et al. (2017a)	POI ligand-linker-OTs, K_2_CO_3_, DMSO, 50°C, overnight to 2 days	62–74% yield for conjugate used
Shimokawa et al. (2017)	POI ligand-linker-OTs, K_2_CO_3_, DMF, 50 C, overnight	75% yield for conjugate used

**SCHEME 38:**
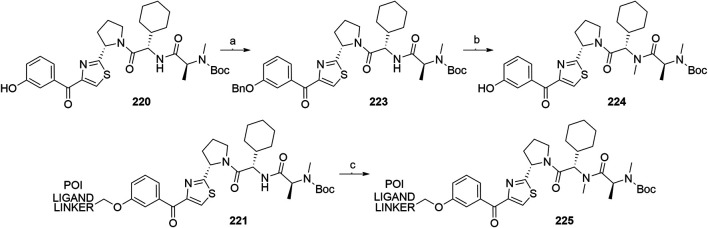
Syntheses of negative control degraders. Reagents and conditions: a) benzyl bromide, K_2_CO_3_, DMF, rt, 4 h, 87% yield; b) i. NaH, DMF, 0°C, 10 min, then MeI, 0°C, 1 h, 81% yield; ii. TFA, rt, 12 h; iii. (Boc)_2_O, THF, rt, 12 h, 34% yield over two steps (Ohoka et al., 2017b); c) MeI, 60% NaH in mineral oil, DMF, rt, 1 h, 68% yield for conjugate used (Shimokawa et al., 2017).

An *N*-methylated analog of **LCL-161** loses the ability to bind to IAP, which allows for the use of such derivatives as negative controls (Ohoka et al., 2017b). The methylation can be achieved before linker attachment by first protecting the phenol group of **220** to obtain benzyl protected derivative **223**, followed by *N*-methylation using NaH and MeI to form **224** (Scheme 38, steps a–b) (Ohoka et al., 2017b). Alternatively, the *N*-methylation of the POI ligand-linker-**LCL-161** conjugates **221** was reported (Shimokawa et al., 2017). In the latter case, however, other possible liable atoms for methylation should be identified before performing the reaction.

#### Inhibitor of Apoptosis Proteins Ligand D

**SCHEME 39:**
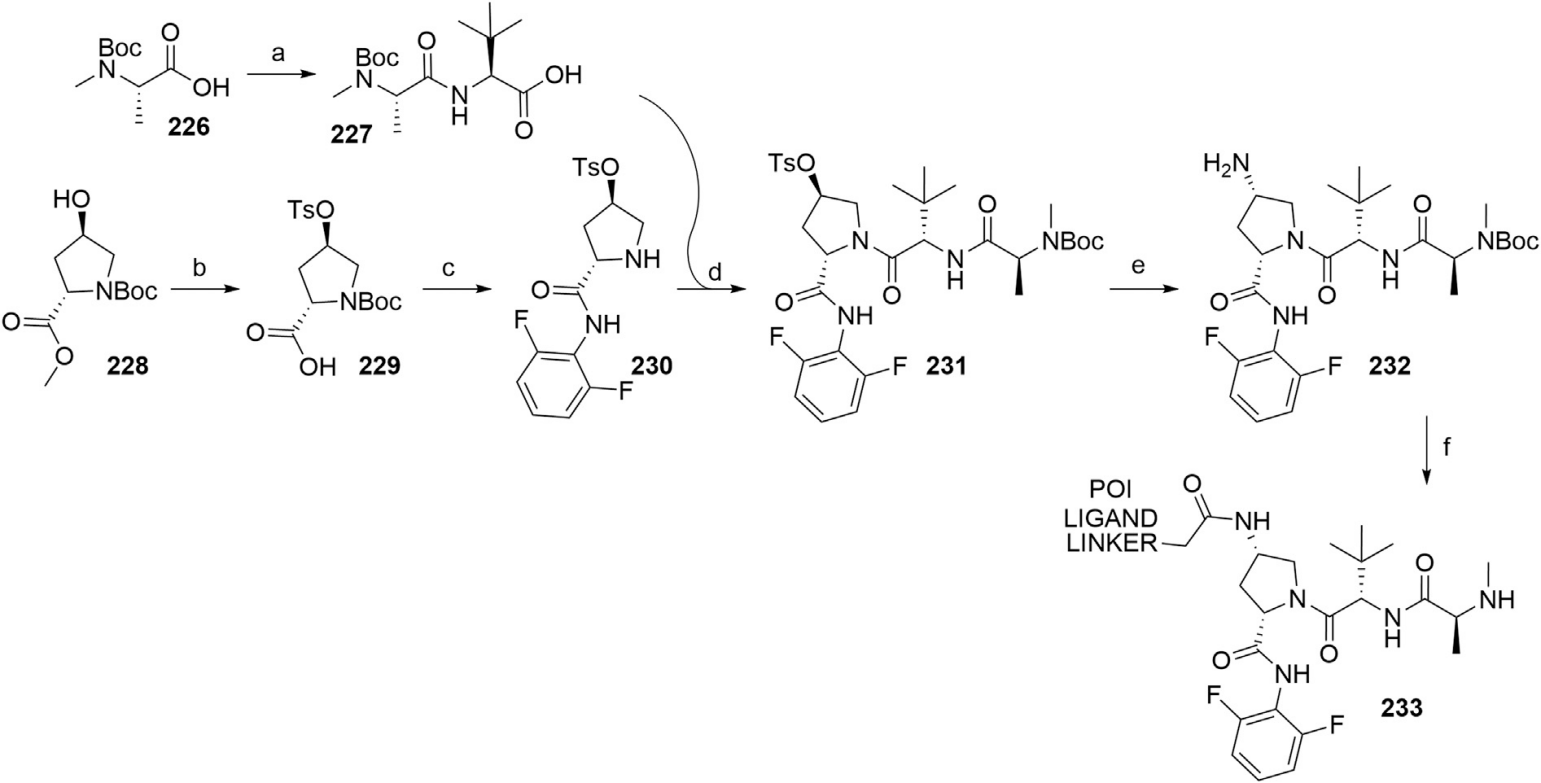
Synthesis of Boc-protected **IAP ligand D** (**232**) and derived SNIPERs **233**. Reagents and conditions: a) i. l-*tert*-Leucine methyl ester, EDC, HOBt, THF, rt, 18 h, 90% yield; ii. LiOH, H_2_O, MeOH, MeOH, THF, rt, 18 h, 84% yield (Casillas et al., 2016); a) i. l-*tert*-Leucine methyl ester, EDC, HOBt, 4-methylmorpholine, CH_2_Cl_2_, 0°C to rt, overnight; ii. LiOH, H_2_O, MeOH, MeOH, THF, rt, 18 h, 99% yield over two steps (Mares et al., 2020); b) i. TsCl, DIPEA, DMAP, CH_2_Cl_2_, rt, overnight; ii. LiOH, MeOH, H_2_O, rt, overnight, 82% yield over two steps; c) i. 2,6-difluoroaniline, DCC, CH_2_Cl_2_, rt, overnight; ii. TFA, CH_2_Cl_2_, rt, 3 h, 66% yield over two steps; d) EDC, HOBt, CH_2_Cl_2_, –20°C, 1 h, then rt, overnight, 54% yield; e) i. NaN_3_, DMF, 80°C, overnight; ii. Pd/C, H_2_, MeOH, rt, 3 h, 10% yield; f) i. POI ligand-linker-CO_2_H, HATU, DIPEA, DMF, rt, 3 h; ii. 4 M HCl in dioxane, rt, 3 h, 8% yield for conjugate used (Anderson et al., 2020; Mares et al., 2020).

Building block **227** was synthesized from *N*-(*tert*-butoxycarbonyl)-*N*-methyl-l-alanine (**226**) as starting compound using EDC/HOBt-mediated coupling and subsequent hydrolysis of the methyl ester (Casillas et al., 2016; Mares et al., 2020). Compound **227** was then coupled with **230**, which was synthesized by subsequent protection of the hydroxyl group and deprotection of the carboxylic acid of **228** to form (2*S*,4*R*)-1-(*tert*-butoxycarbonyl)-4-(tosyloxy)pyrrolidine-2-carboxylic acid (**229**), followed by the coupling of 2,6-difluoroaniline. The tosylate group of **231** was transformed into an azide and then reduced to an amine yielding **232**, which was then coupled with a carboxylic acid-containing POI ligand-linker conjugate and Boc-deprotected to yield final SNIPER compounds **233** (Scheme 39) (Anderson et al., 2020, 6; Mares et al., 2020).

#### Inhibitor of Apoptosis Proteins Ligand E: A410099 Derivative

**SCHEME 40:**
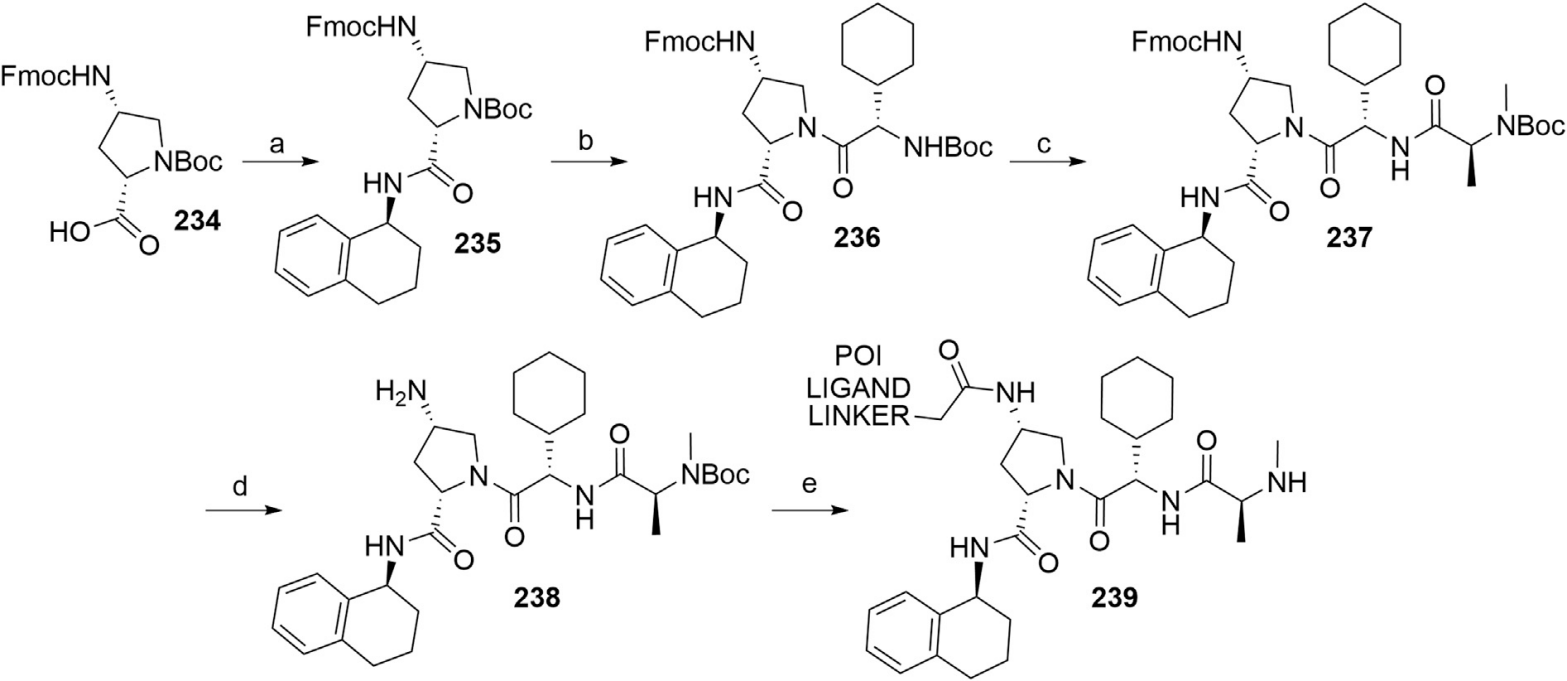
Synthesis of Boc protected **IAP ligand E** (compound **238**) and derived degraders **239**. Reagents and conditions: a) (*S*)-1,2,3,4-tertahydronaphthalen-1-amine, EDC, HOAt, NMP; b) i. TFA; ii. Boc-l-cyclohexylglycine, EDC, HOAt, NMP; c) i. TFA; ii. Boc-*N*-methyl-l-alanine, EDC, HOAt, NMP; d) piperidine (Note: no yields, solvents, temperatures, or reaction times given) (Borzilleri et al., 2014); e) i. POI ligand-linker-CO_2_H, HATU, Et_3_N, DMF, rt, 15 h; ii. TFA, rt, 16 h, 30–54% yield for conjugates used over two steps (Shah et al., 2020); e) i. linker-CO_2_H, HATU, DIPEA, DMF, rt, 16 h, 79–91% yield for conjugates used; ii. 4 M HCl in dioxane, rt, 2 h, 91–96% yield for conjugates used; iii. POI ligand attachment (Nunes et al., 2019).

The synhesis of **IAP ligand E** is described in patents and consists of a stepwise coupling procedure (Scheme 40). The orthogonally protected **234** was first coupled with (*S*)-1,2,3,4-tertahydronaphthalen-1-amine to form **235**, which was then Boc-deprotected and coupled with Boc-l-cyclohexylglycine. The resulting **236** was Boc-deprotected and coupled with Boc-*N*-methyl-l-alanine to yield **237**. The Fmoc protection of 4-amino group on the proline fragment allowed for the selective deprotection to obtain **238** (Borzilleri et al., 2014; Mischke, 2014). Carboxylic acid-containing POI ligand-linker conjugates were attached and the *N*-terminal amino group was Boc-deprotected to give final SNIPER compounds **239** (Shah et al., 2020). As an alternative, **238** was coupled with a carboxylic acid-containing linker, the product was Boc-deprotected and the POI ligand was attached to obtain the desired SNIPER compounds (Nunes et al., 2019).

#### Inhibitor of Apoptosis Proteins Ligand F

**SCHEME 41:**
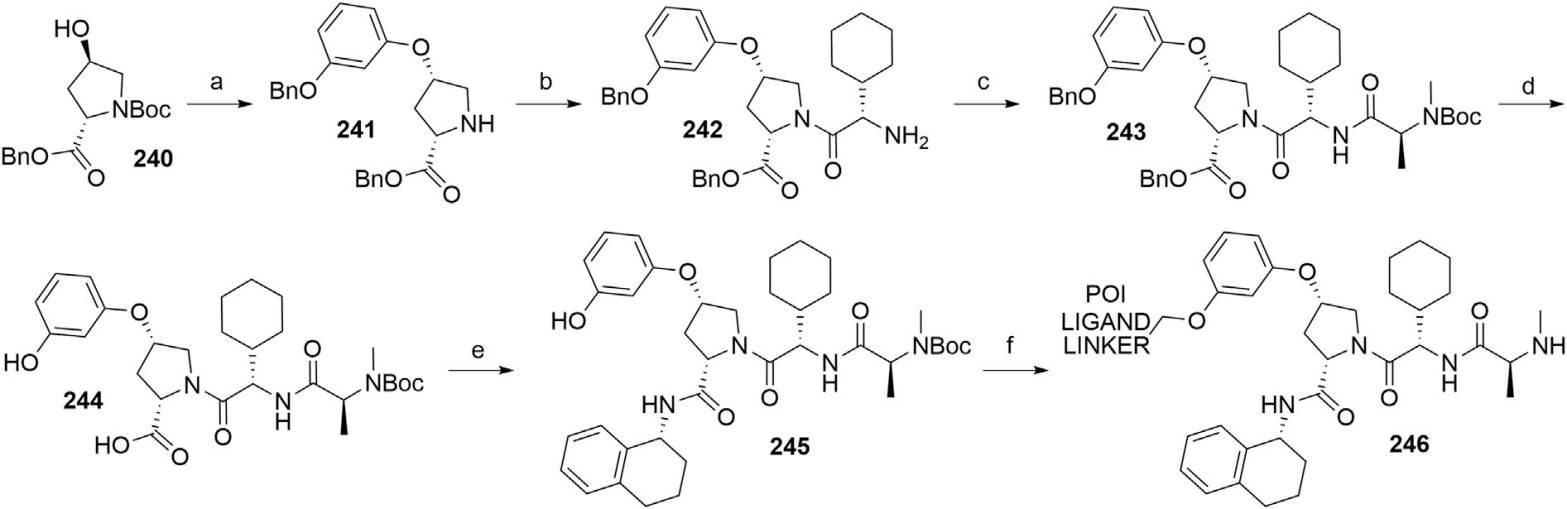
Syntheses of Boc-protected **IAP ligand F** (compound **245**) and derived SNIPERs **246**. Reagents and conditions: a) i. 3-(benzyloxy)phenol, PPh_3_, DEAD, THF, –10°C to rt, 16 h, 32% yield; ii. 4 M HCl in EtOAc, 0°C to rt, 1 h, 76% yield; b) i. Boc-l-cyclohexylglycine, EDC, HOBt, DIPEA, CH_2_Cl_2_, 0°C to rt, 16 h, 99% yield; ii. 4 M HCl in EtOAc, rt, 16 h, 99% yield; c) Boc-*N*-methyl-l-alanine, EDC, HOBt, DIPEA, CH_2_Cl_2_, rt, 16 h, 77% yield; d) Pd/C, H_2_, MeOH, rt, 16 h, 95% yield; e) (*R*)-1,2,3,4-tetrahydronaphthalen-1-amine, EDC, HOBt, DIPEA, CH_2_Cl_2_, rt, 16 h, 36% yield; f) i. POI ligand-linker-OTs, K_2_CO_3_, DMF, 60°C, 6 days, 91% yield for conjugate used; ii. 6 M HCl, THF, rt, 4 h, 46% yield (Ohoka et al., 2018); f). linker-OMs, K_2_CO_3_, DMF, 60°C, 24 h; ii. POI ligand attachment; iii. 1 M HCl in EtOAc, rt, 4 h, 79% yield for conjugates used (Steinebach et al., 2020a).

A procedure towards **IAP ligand F** used appropriately di-protected 4-hydroxyproline **240** as a starting compound, which was subjected to Mitsunobu conditions to attach 3-(benzyloxy)phenol, giving compound **241** (Ohoka et al., 2018). 4-(Benzyloxy)phenol was also used in place of 3-(benzyloxy)phenol to yield a *para-*hydroxy substituted IAP ligand. The right-hand side of the molecule was then assembled through coupling reactions with Boc-l-cyclohexylglycine to yield **242** and Boc-*N*-methyl-l-alanine to produce **243**. Pd/C-catalyzed hydrogenation cleaved both benzyl protecting groups, resulting in compound **244**, which was then coupled with (*R*)-1,2,3,4-tetrahydronaphthalen-1-amine into **245**. A tosylate-containing POI ligand-linker conjugate was then attached, and the compounds were Boc-deprotected to give final products **246** (Scheme 41) (Ohoka et al., 2018). Alternatively, a mesylate-containing linker was incorporated, followed by the POI linker attachment and Boc-deprotection (Steinebach et al., 2020a).

#### Inhibitor of Apoptosis Proteins Ligand G

**SCHEME 42:**
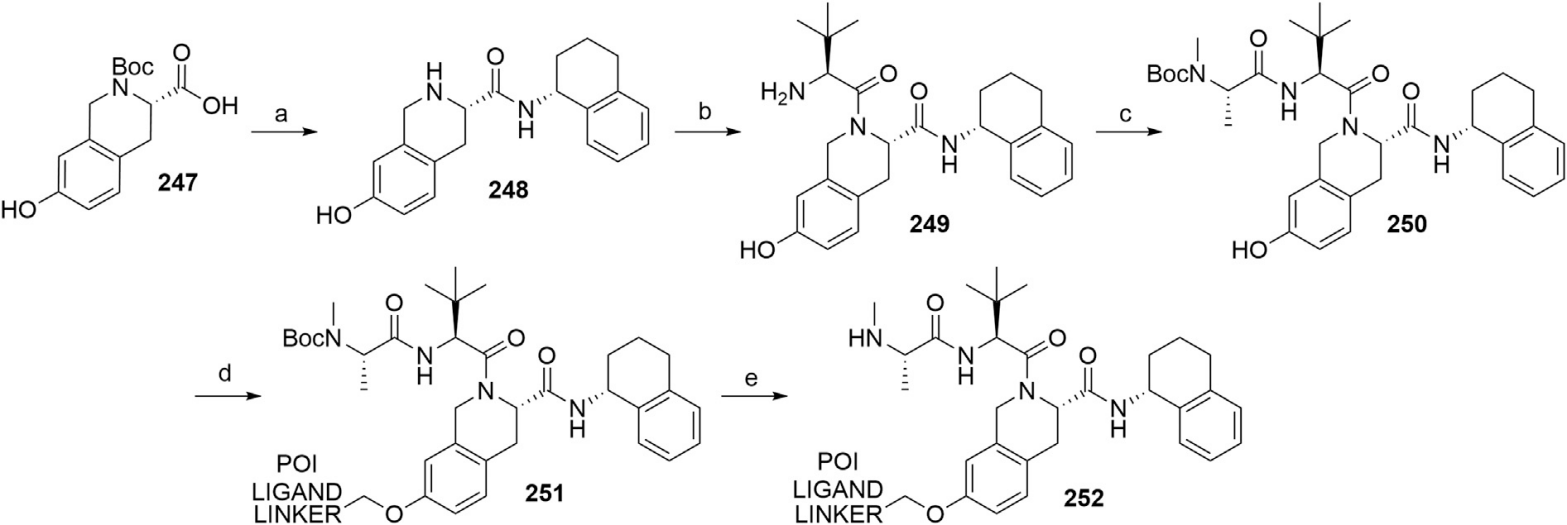
Synthesis of Boc-protected **IAP ligand G** (**250)** and derived degraders **246**. Reagents and conditions: a) i. (*R*)-1,2,3,4-tetrahydronaphthalen-1-amine, HATU, DIPEA, DMF, rt, 30 min, 82% yield; ii. 4 M HCl in dioxane, THF, rt, overnight, 95% yield; b) i. Boc-Tle-OH, HATU, DIPEA; DMF, rt, 1 h, 66% yield; ii. 4 M HCl in dioxane, THF, rt, overnight, 97% yield; c) Boc-*N*-methyl-l-alanine, HATU, DIPEA, DMF, rt, overnight, 77% yield; d) POI ligand-linker-Cl, K_2_CO_3_, DMF, 80°C, overnight, 63% yield for conjugate used; e) TFA, CH_2_Cl_2_, rt, 3 h, 54% yield for conjugate used (Casillas et al., 2016; Mares et al., 2020).

Compound **250** was synthesized by first combining 1,2,3,4-tetrahydroisoquinoline **247** with (*R*)-1,2,3,4-tetrahydronaphthalen-1-amine and Boc-deprotecting the product to give **248**. Coupling with Boc-l-*tert*-leucine and subsequent Boc-deprotection yielded **249**, which was further converted with Boc-*N*-methyl-l-alanine to give **250**. A POI ligand-linker chloro conjugate was then connected *via* the phenol group, giving compound **251**, which was Boc-deprotected to afford final SNIPER molecules **252** (Scheme 42) (Casillas et al., 2016; Mares et al., 2020).

#### Inhibitor of Apoptosis Proteins Ligand H

**SCHEME 43:**
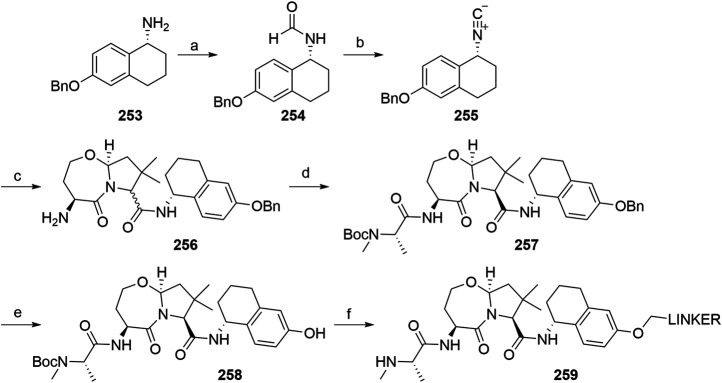
Synthesis of **IAP ligand H**. Reagents and conditions: a) ethyl formate, 80°C, 16 h, 84% yield; b) POCl_3_, Et_3_N, CH_2_Cl_2_, 0 °C to rt, 1 h, 89% yield; c) i. *N*-Boc-l-homoserine, 4,4-dimethoxy-2,2-dimethylbutanal, 7 M NH_3_ in MeOH, rt, 16 h; ii. TFA, CH_2_Cl_2_, rt, 16 h, 74% yield over two steps; d) Boc-*N*-methyl-l-alanine, HATU, DIPEA, CH_2_Cl_2_, rt, 1 h, 38% yield; e) Pd(OH)_2_, H_2_, MeOH, 40°C, 2 h, quant.; f) i. linker-OTs or -Br, K_2_CO_3_, NaI, DMF, 70°C, overnight; ii. TFA, CH_2_Cl_2_, rt, 16 h (Note: yields not given) (Zhang et al., 2020b).

The synthesis of **IAP ligand H (** Zhang et al., 2020b) started with the treatment of compound **253** with ethyl formate to form formamide **254**. Isocyanide **255** was obtained by dehydration in the presence of POCl_3_ and triethylamine. The 7,5-heterobicycle **256** was obtained as a mixture of diastereomers through an Ugi four-component reaction and subsequent treatment with trifluoroacetic acid. Coupling with Boc-*N*-methyl-l-alanine yielded a mixture of diastereomers, and flash column separation gave the desired compound **257** in a 38% yield. Hydrogenolysis cleaved the benzyl protective group and gave product **258**, to which tosylate- or bromo-containing linkers were attached to form conjugates **259** (Scheme 43) (Zhang et al., 2020b).

#### Inhibitor of Apoptosis Proteins Ligand I

**SCHEME 44:**
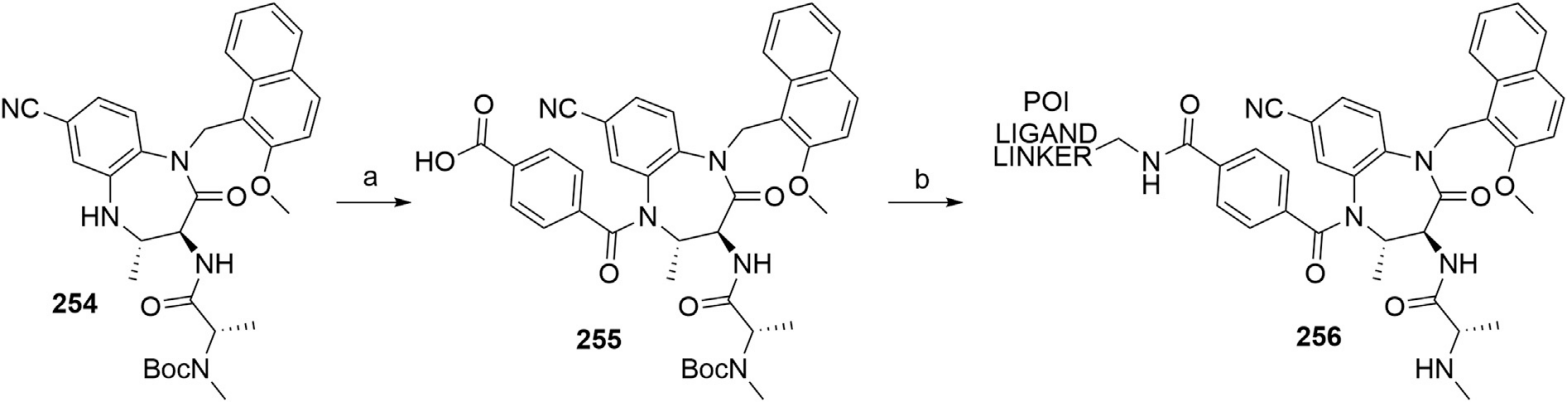
Synthesis of **IAP ligand I**-based PROTAC **256**. Reagents and conditions: a) i. methyl 4-(chlorocarbonyl)benzoate, Et_3_N, 1,2-dichloroethane, 80°C, 24 h, 59% yield; ii. LiOH × H_2_O, THF/H_2_O, rt, 1 h; b) i. POI ligand-linker-NH_2_, HATU, DIPEA, DMF, rt, 1 h, 68% yield for conjugate used; ii. 4 M HCl in dioxane, CH_2_Cl_2_, rt, 1 h, 41% yield for conjugate used (Dragovich et al., 2020).

**IAP ligand I** was utilized in PROTACs (Dragovich et al., 2020) and could be synthesized based on the following procedure (Kester et al., 2013). By coupling compound **254** with methyl 4-(chlorocarbonyl)benzoate and the subsequent methyl ester hydrolysis, intermediate **255** was formed. POI ligand-linker-amine conjugates were then coupled, and the obtained products were Boc-deprotected to give the final PROTAC compound **256** (Scheme 44) (Dragovich et al., 2020).

#### Statistical Overview of Utilized Inhibitor of Apoptosis Proteins Ligands

**FIGURE 7:**
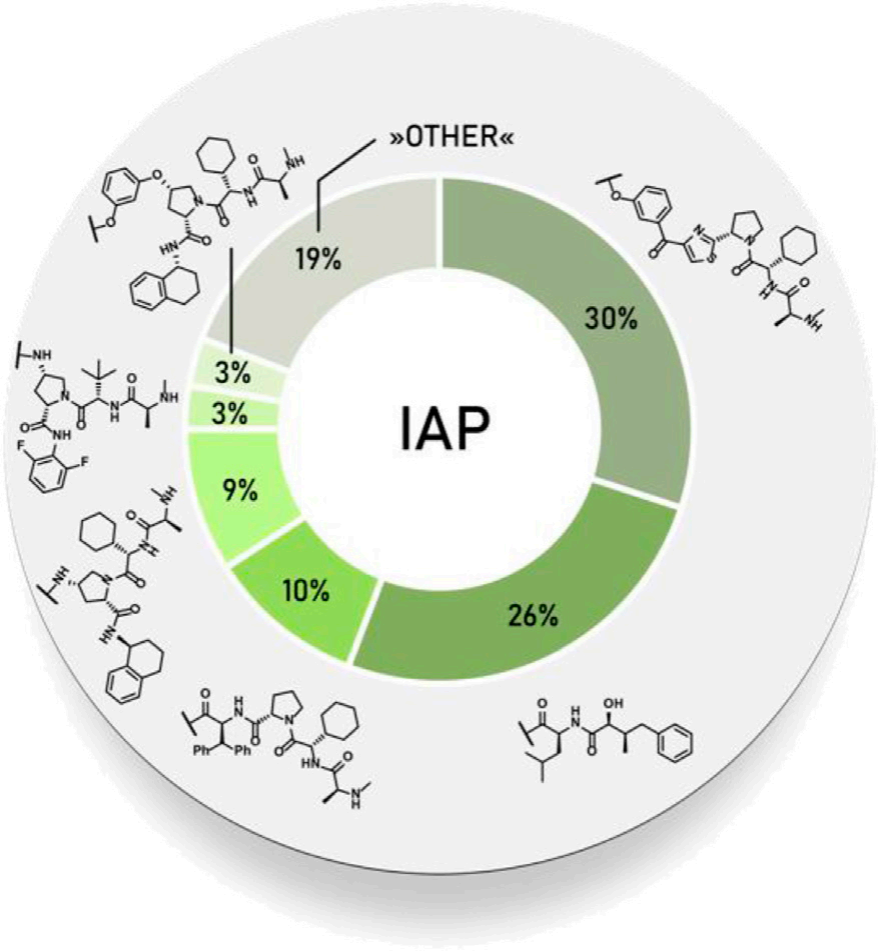
Frequency of IAP ligands used in PROTAC compounds.

Using data extracted from PROTAC-DB (Weng et al., 2021) (http://cadd.zju.edu.cn/protacdb/, as of the February 26, 2021) a statistical overview was done to determine the frequency of various IAP ligands and linker attachment options used in PROTAC compounds (Figure 7). **LCL-161** derivatives were most commonly utilized, with around 30% of PROTACs incorporating its structure. Following closely was bestatine, with **MV1** derivatives and IAP ligand **E** having a lower presence at 10 and 9%, respectively. Other IAP ligands are less common, with fewer than 3% representation.

### MDM2

The p53 protein is a product of the tumor-suppressor gene and acts as a transcription factor that gets activated when cell stress occurs, especially upon the occurrence of DNA damage. Activation of the p53 network results in the inhibition of the cell cycle and can lead to apoptosis of damaged cells to prevent their unhindered growth, thus acting as an important tumor suppressor (Levine, 1997; Vogelstein et al., 2000). The effects of p53 are controlled in an autoregulatory negative feedback loop involving MDM2, which belongs to the family of RING finger ubiquitin ligases (Michael and Oren, 2003). p53 induces the expression of MDM2, which in turn leads to the repression of p53 activity through binding of MDM2 to p53 and blocking its function, as well as through MDM2-mediated ubiquitination and subsequent degradation of p53 by the proteasome (Michael and Oren, 2003; Vassilev et al., 2004). Excessive activity of MDM2 has been observed in numerous malignancies, which makes it a promising target for the treatment of tumors due to its dual-mode mechanism of interaction with p53 (Momand et al., 1998).

**FIGURE 8:**
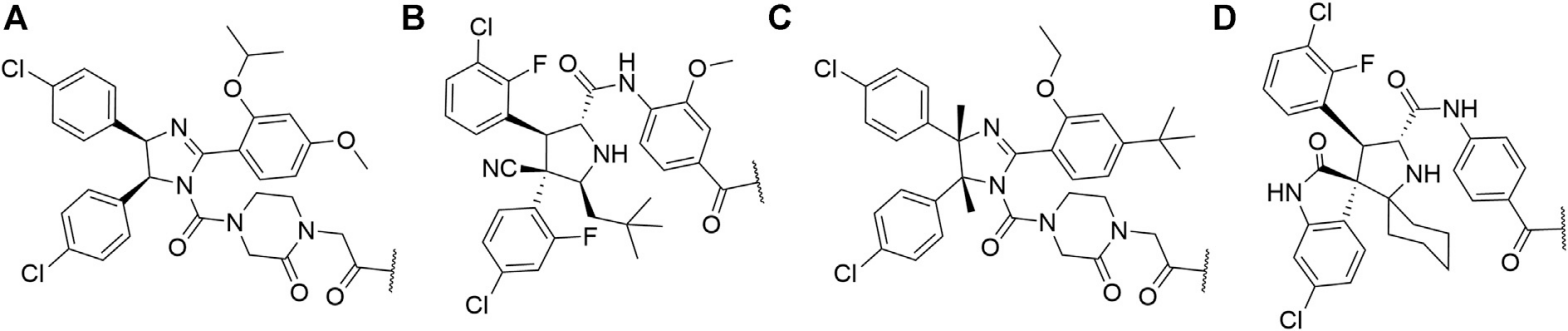
MDM2 ligands. **(A)** Nutlin-3a derivative, **(B)** Idasanutlin derivative, **(C)** RG7112-derivative, **(D)** MI-1061 derivative.

The E3 ligase activity of MDM2 was utilized in the first small-molecule PROTAC by incorporating a molecule belonging to a class of imidazoline derivatives called nutlins, which bind to MDM2 in a nano-to micromolar range (Vassilev et al., 2004; Schneekloth et al., 2008). Additional proteins to be successfully degraded through MDM2-mediated ubiquitination include BTK (Sun et al., 2018), PARP1 (Zhao et al., 2019), and TrkC (Zhao and Burgess, 2019), among others. Utilized ligands are collected in Figure 8.

#### Nutlin-3-Based Ligands and Derivatives

**SCHEME 45:**
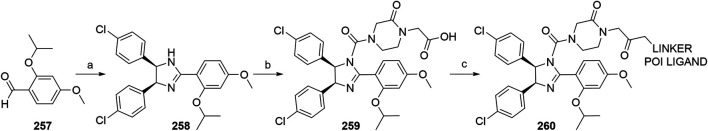
Synthesis of Nutlin-3a derivative **259** and degraders **260**. Reagents and conditions: a) 1,2-bis(4-chlorophenyl)-1,2-ethanediamine, CH_2_Cl_2_, 0°C, 2 h, then NBS, 0°C to rt, 16 h, 88% yield; b) i. triphosgene, Et_3_N, THF, 0°C, 2.5 h, 96% yield; ii. *tert*-butyl 2-(2-oxopiperazin-1-yl)acetate, CH_2_Cl_2_, 0°C, 1.5 h, 96% yield; iii. TFA, CH_2_Cl_2_, 96% yield; c) POI ligand-linker-NH_2_, HATU, DIPEA, DMF, rt, 16.5 h, 61% yield for conjugate used (Schneekloth et al., 2008); c) linker-NH_2_, HATU, Et_3_N, DMF, CH_2_Cl_2_, 5 h, 63% yield for linker used (Zhao et al., 2019).

The first nutlin-derived PROTAC (Schneekloth et al., 2008) has been prepared as follows. Starting material **257** was first reacted with meso-1,2-bis(4-chlorophenyl)-1,2-ethylenediamine to form imidazoline **258** *via* a radical pathway induced by NBS. Triphosgene was used to create an activated formic acid ester which was then reacted with *tert*-butyl 2-(2-oxopiperazin-1-yl)acetate and deprotected by TFA to give **259** in an 88% yield over three steps(Schneekloth et al., 2008). HATU-facilitated coupling was then employed to attach POI ligand-linker-NH_2_ conjugates to form **260** (Scheme 45) (Schneekloth et al., 2008; Zhao et al., 2019).

**SCHEME 46:**
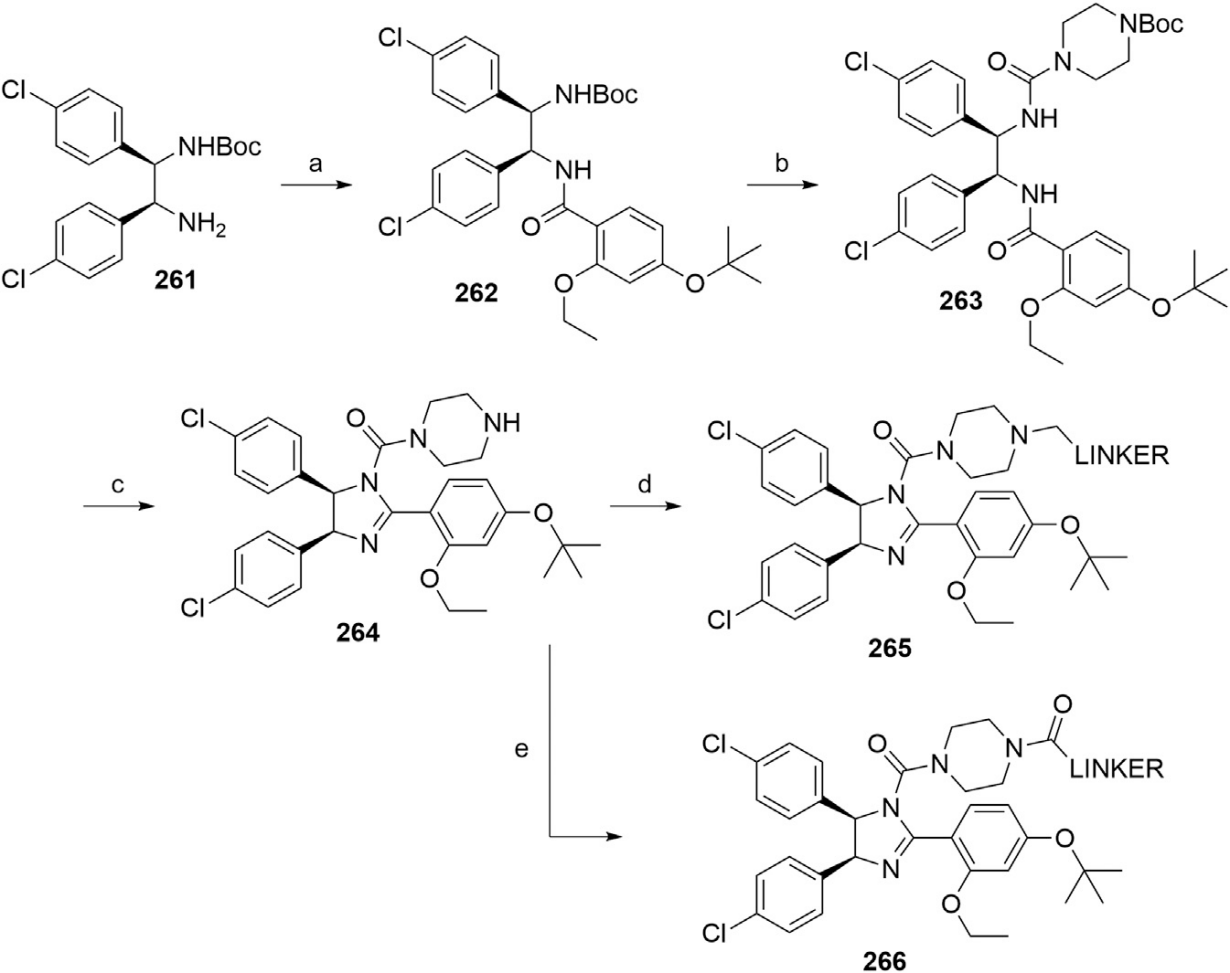
Synthesis of Nutlin-3 derivative **264** and linker conjugates **265** and **266**. Reagents and conditions: a) 4-(*tert*-butoxy)-2-ethoxybenzoic acid, EDC, DMAP CH_2_Cl_2_, rt, 16 h, 96% yield; b) i. TFA, CH_2_Cl_2,_ rt, 30 min, 92%; ii. CDI, CH_2_Cl_2_, *N*-Boc-piperazine, rt, overnight, 84% yield; c) TPPO, triflic anhydride, CH_2_Cl_2_, 0°C, 1.5 h, 97% yield; d) Br-linker, K_2_CO_3_, THF, reflux, overnight, 61–72% for linkers used; e) linker-CO_2_H, HATU, DIPEA, DMF, rt, 3 h, 83–98% yield for linkers used (Wang et al., 2019).

Close Nutlin-3a derivatives were also incorporated into PROTACs (Wang et al., 2019). Starting material **261** was coupled with 4-(*tert*-butoxy)-2-ethoxybenzoic acid to yield **262**, which was subsequently Boc-deprotected and underwent a CDI-mediated urea formation to obtain **263**. Cyclization to **264** was done using TPPO and triflic anhydride. Linker attachment was obtained by alkylation using bromo linkers alongside K_2_CO_3_ under reflux or by coupling with carboxylic acid linkers (Scheme 46) (Wang et al., 2019).

**SCHEME 47:**
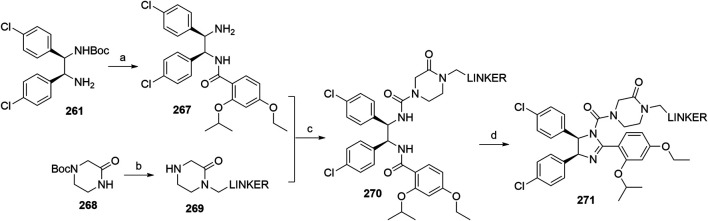
Alternative synthesis of Nutlin-3 derivative—linker conjugates **271**. Reagents and conditions: a) i. 2-isopropoxy-4-methoxybenzoic acid, EDC, DMAP, CH_2_Cl_2_, rt, 17 h, 79% yield; ii. TFA, CH_2_Cl_2_, rt, 3 h, 97% yield; b) i. Br-linker, NaH, DMF, 0°C to rt, 15 h, 75% yield for linker used; ii. TFA, CH_2_Cl_2_, rt, 5 h, 82% yield for conjugate used; c) CDI, CH_2_Cl_2,_ rt, 2 h, then **269**, rt, 14 h, 86% yield for conjugate used; d) triflic anhydride, TPPO, CH_2_Cl_2_, 45°C, 15 h, quant. for conjugate used (Nietzold et al., 2019).

An alternative synthetic strategy was devised (Scheme 47), by attaching the linker to *tert*-butyl 3-oxopiperazine-1-carboxylate **268** prior to urea bond formation with **267** to give compound **270**. Cyclization to **271** was obtained similarly as depicted in Scheme 45 (Nietzold et al., 2019).

#### Idasanutlin-Based Ligands

**SCHEME 48:**
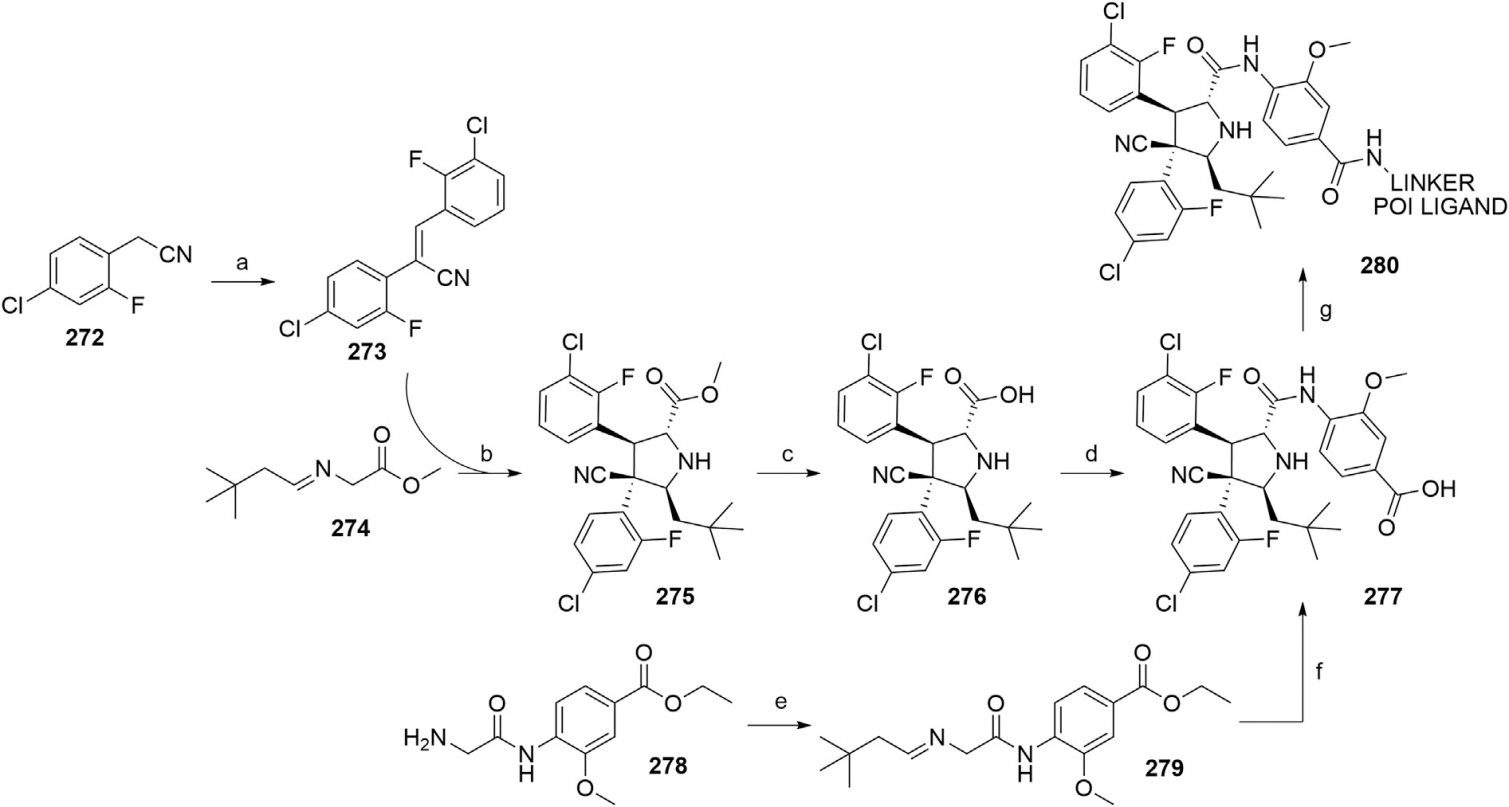
Synthesis of idasanutlin **277**. Reagents and conditions: a) 3-chloro-2-fluorobenzaldehyde, NaOMe, MeOH, 50°C, 3 h (yield not given) (Ding et al., 2013); a) 3-chloro-2-fluorobenzaldehyde, NaOMe, MeOH, EtOH, H_2_O, 50°C, 3 h, 91% yield (Rimmler et al., 2016, 2); b) AgF, Et_3_N, 1,2-dichloroethane, 31% yield; c) i. 2 M NaOH, MeOH, 97% yield; ii. chiral SFC separation, 49% yield; d) i. methyl 4-amino-3-methoxybenzoate, Ph_2_POCl_2_, DIPEA, CH_2_Cl_2_, 81% yield; ii. NaOH (aq), MeOH, THF, 80% yield (Shu et al., 2016); e) 3,3-dimethylbutanal, Et_3_N, MTBE, rt, 6 h, 94% yield (Rimmler et al., 2016); f) i. **273**, AgOAc, (*R*)-MeOBIPHEP, 2-Me-THF, 2–4°C, 16 h; ii. LiOH, 2-Me-THF, 65°C, 19 h, 97% yield over 2 steps; iii. LiOH, isopropanol, 63–67°C, 6 h then 15–20°C, 8 h, 45% yield; f) i. **273**, (*R*)-BINAP, CuOAc, Et_3_N, 2-Me-THF, 5 h; ii. NaOH (aq), THF, EtOH, rt, 18 h, 78% yield over 2 steps; iii. THF, EtOAc, 65°C, 2 h, 79% yield (Rimmler et al., 2016, 2); f) i. **273**, Cu(MeCN)_4_PF_6_/*ent*-Phosferrox, Cs_2_CO_3_, THF, –40°C, 93% yield; ii. NaOH (aq), THF, EtOH, rt, 18 h; iii. THF, EtOAc, 65°C, 2 h (Zou et al., 2020); g) POI ligand-linker-NH_2_, HATU, DIPEA, MeCN, rt, 30 min, 42–60% yield for conjugates used (Zhang et al., 2020c); POI ligand-linker-NH_2_, HATU, DIPEA, DMF, rt, 24 h, 28% yield for conjugate used (Hines et al., 2019).

The established synthesis of idasanutlin begins with a Knoevenagel condensation between starting material **272** and 3-chloro-2-fluorobenzaldehyde (Ding et al., 2013; Rimmler et al., 2016). Compound **273** can then be joined with **274** to give methyl ester **275**, which is then hydrolyzed and coupled with methyl 4-amino-3-methoxybenzoate and again hydrolyzed to yield idasanutlin **277**. The procedure requires a chiral chromatographic phase for separation of the desired product (Rimmler et al., 2016). Alternatively, imine **279** can be formed using 3,3-dimethylbutanal and **278**, followed by a Ag(I) or Cu(I) catalyzed asymmetric [3 + 2] cycloaddition. Isomerization with NaOH in THF/EtOH yields the desired stereoisomer (Rimmler et al., 2016; Zou et al., 2020). POI ligand-linker-NH_2_ conjugates were coupled with idasanutlin to yield MDM2-targeting PROTACs **280** (Hines et al., 2019; Zhang et al., 2020c). By starting the PROTAC preparation with racemic idasanutlin, the chiral separation was performed after the coupling reaction (Hines et al., 2019) (Scheme 48).

#### RG7112-Based Derivatives

**SCHEME 49:**
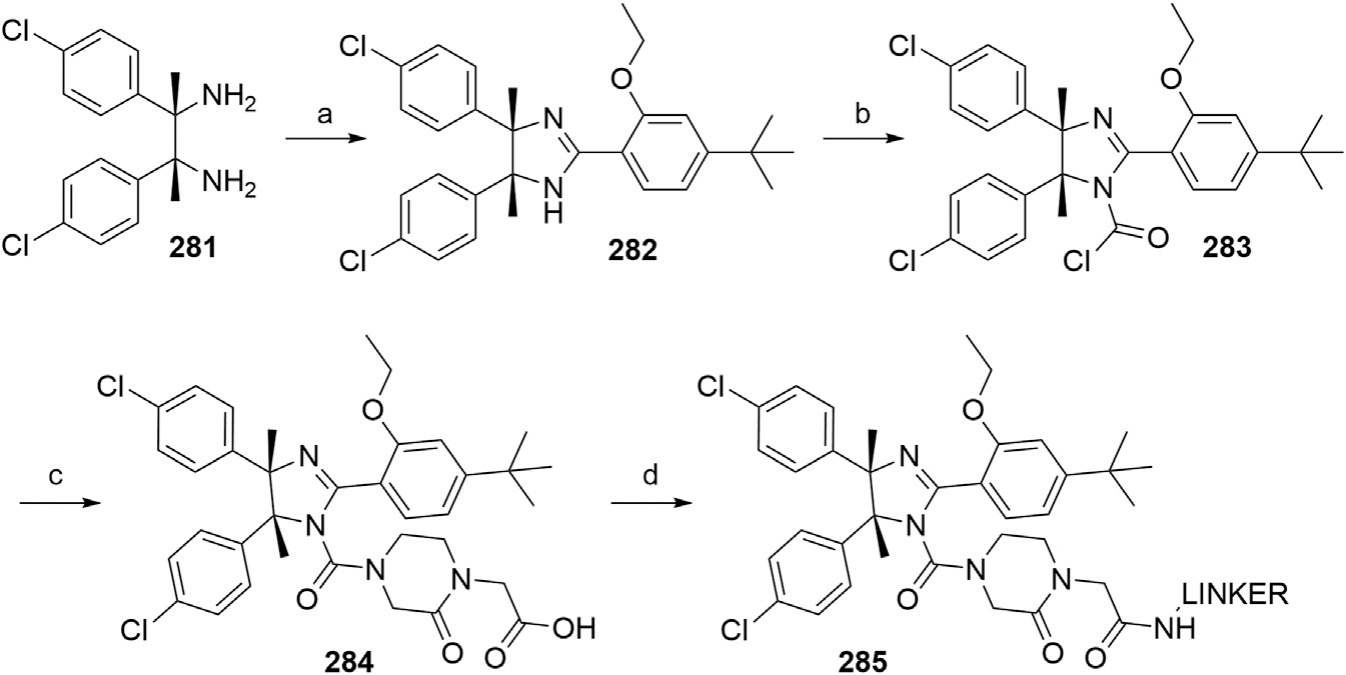
Synthesis of RG7112 derivative **284** and linker attachment to form conjugate **285**. Reagents and conditions: a) AlMe_3_, toluene, 0°C to rt, 20 min, then methyl 4-(*tert*-butyl)-2-ethoxybenzoate, reflux, 2.5 h, 18% yield; b) Et_3_N, phosgene, toluene, 0°C, 30 min, 87% yield; c) exact procedure is not disclosed; piperazine derivative, Et_3_N, CH_2_Cl_2_, 0°C, 7 h (Vu et al., 2013); d) linker-NH_2_, HATU, DIPEA, DMF, rt, 2 h, 51% yield for linker used (Sun et al., 2018).

Diamine **281** was reacted with methyl 4-(*tert*-butyl)-2-ethoxybenzoate using trimethylaluminum to give imidazoline **282**. Carbamoyl chloride **283** was obtained through treatment with phosgene. Coupling with an appropriate piperazine derivative (exact procedure is not described) afforded compound **284**, which was then coupled with amine linkers to yield derivatives **285** (Scheme 49) (Vu et al., 2013; Sun et al., 2018).

#### MI-1061-Based Derivatives

**SCHEME 50:**
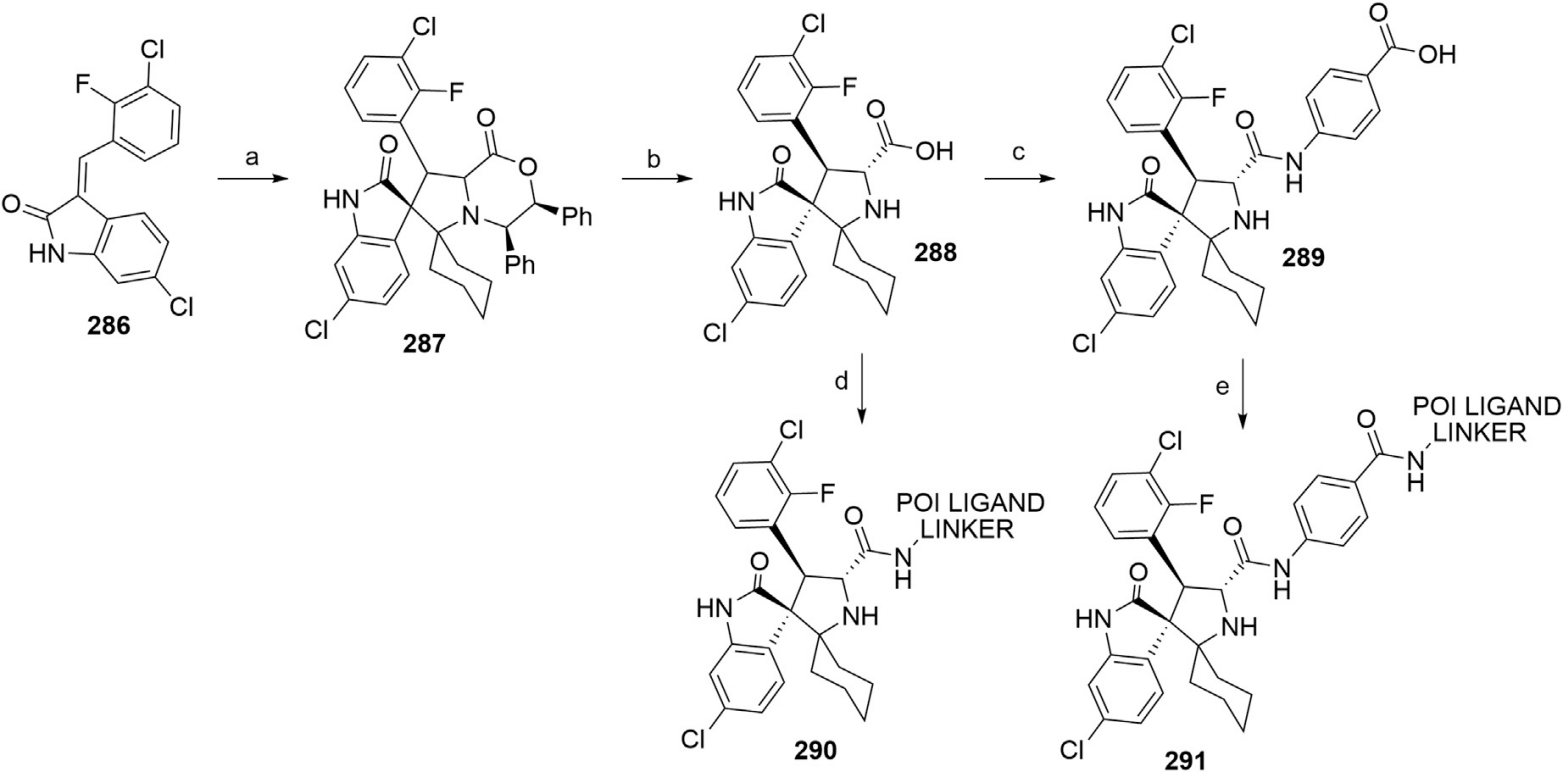
Synthesis of MI-1061 based derivative **289**. Reagents and conditions: a) (*5R*,*6S*)-5,6-diphenyl-2-morpholinone, cyclohexanone, toluene, 140°C, 2 h, 64% yield; b) i. conc. H_2_SO_4_, MeOH, 50°C, 57% yield; ii. MeCN, H_2_O, CAN, rt, 30 min, 92% yield; c) i. CDI, DIPEA, DMAP, 1,2-dichloroethane, 40°C, 30 min; ii. methyl 4-aminobenzoate, reflux, overnight, 41% yield; ii. LiOH × H_2_O, NaOH, THF, MeOH, rt, 2 h; ii. TFA, rt, briefly, 31% (Aguilar et al., 2014); d) POI ligand-linker-NH_2_, HATU, DIPEA, DMF, rt, 10 min (Yang et al., 2019); e) POI ligand-linker-NH_2_, HATU, DIPEA, DMF, DMSO rt, 30 min, 17–75% yield for conjugates used (Li et al., 2019).

Further development of MDM2 inhibitors yielded the potent and efficacious compound MI-1061 (**289**, Scheme 50). Using starting material **286**, an asymmetric 1,3-dipolar cycloaddition reaction was employed to create the spiro-oxindole scaffold **287**. Ring-opening and oxidative removal of the chiral auxiliary (1,2-diphenylethanol) on the pyrrolidine nitrogen yielded the free carboxylic acid **288**. Following CDI coupling with methyl 4-aminobenzoate and ester hydrolysis yielded inhibitor **289** (Aguilar et al., 2014). Attachment of POI ligand-linker conjugates to **288** (Yang et al., 2019) or incorporation of the full MI-1061 structure (**289**) (Li et al., 2019) provided corresponding PROTACs.

### RNF114

Gene transcription is regulated in a crucial way by the zinc-finger gene family. One of the members is ZNF313, also known as RNF114 or ZNF228, which contains both C_2_H_2_ and RING-finger structure. Along with the *N-*terminal RING domain, it also has a *C*-terminal ubiquitin-interacting motif (UIM), both of which are responsible for RNF114s E3 ligase activity (Bijlmakers et al., 2011; Han et al., 2013). Nimbolide, a limonoid natural product derived from the Neem tree (*Azadirachta indica*), was recently found to primarily target RNF114 by covalently modifying its cysteine-8, thus leading to impaired E3 ligase activity of RNF114. As a result, substrate recognition is blocked, which leads to the stabilization of tumor suppressors p21 and p57, explaining nimbolide’s antiproliferative effects. By incorporating nimbolide into a BRD4-targeting PROTAC, RNF114 was successfully established as a viable E3 ligase option for targeted protein degradation (Spradlin et al., 2019).

#### Nimbolide

**SCHEME 51:**
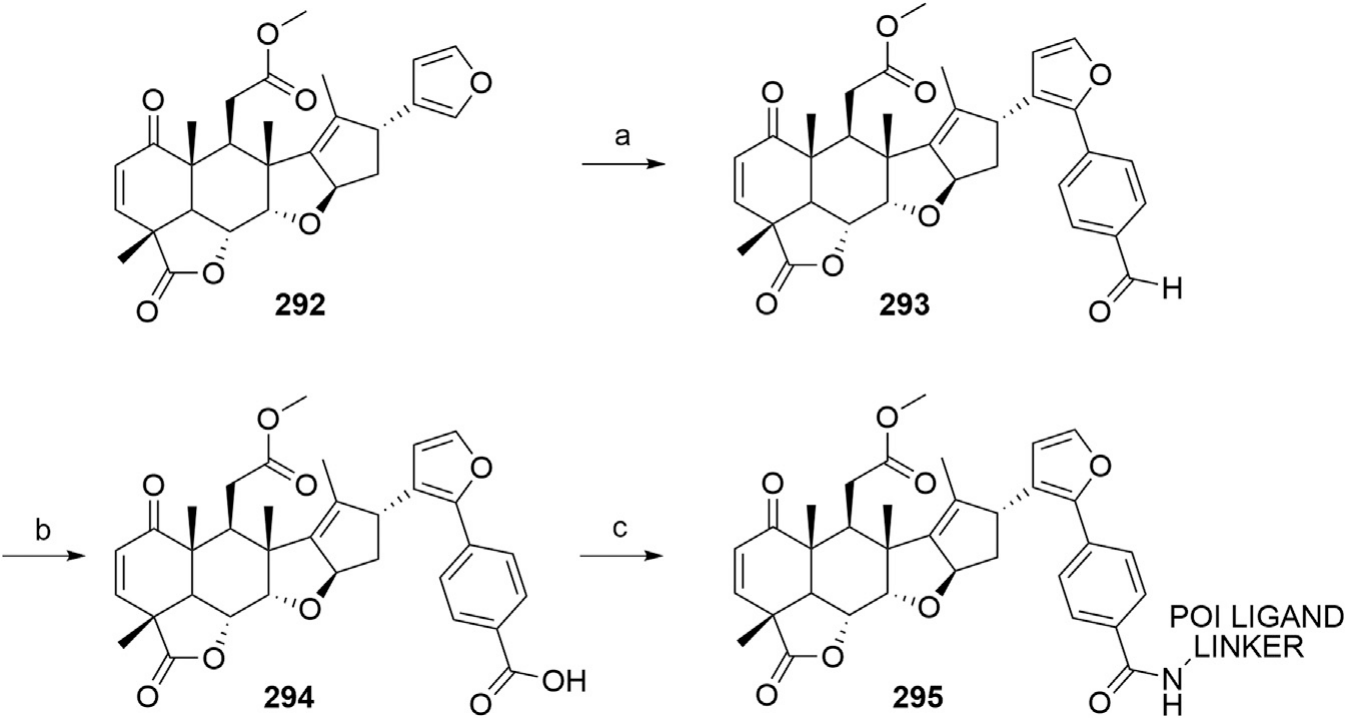
Derivatization of nimbolide (**292**) for use in PROTACs. Reagents and conditions: a) i. NBS, DMF, 0°C, 1 h, 95% yield; ii. 4-formylphenylboronic acid, Pd_2_(dba)_3_, SPhos, K_3_PO_4_, toluene, 60°C, 48 h, 92% yield; b) 2-methyl-2-butene, NaClO_2_, NaH_2_PO_4_, *t*-BuOH, H_2_O, rt, 6 h, yield not given; c) POI ligand-linker-NH_2_, HATU, DIPEA, DMF, 0–4°C, 16 h, 42–45% yield for conjugates used (Spradlin et al., 2019); c) POI ligand-linker-NH_2_, HATU, DIPEA; CH_2_Cl_2_, rt, 10 h, 26–54% yield for conjugates used (Tong et al., 2020b).

A procedure for extracting nimbolide from commercial neem leaf powder has been reported (Tong et al., 2020b). After a maceration of 450 g neem leave powder in anhydrous MeOH, the crude extract was filtered through celite and concentrated under vacuum. The residue was partitioned between EtOAc and H_2_O. The organic phase was then washed with an aqueous solution of NaHCO_3_ and Na_2_S_2_O_3_, dried over Na_2_SO_4_ and concentrated *in vacuo*. Two subsequent column chromatographies on silica gel with hexanes/EtOAc and CH_2_Cl_2_/acetone were performed. Finally, trituration yielded around 1.2 g of nimbolide (Tong et al., 2020b). Derivatization is possible as described (Spradlin et al., 2019). Hence, treatment of nimbolide with NBS led to a selectively brominated product, which was then reacted with 4-formylphenylboronic acid following a Suzuki coupling procedure to yield **293** (Spradlin et al., 2019). Performing a Pinnick oxidation gave carboxylic acid **294**, which was then available for attaching POI ligand-amine linker conjugates through HATU-mediated coupling (Scheme 51) (Spradlin et al., 2019; Tong et al., 2020b).

#### Alternative RNF114 Ligand

**SCHEME 52:**
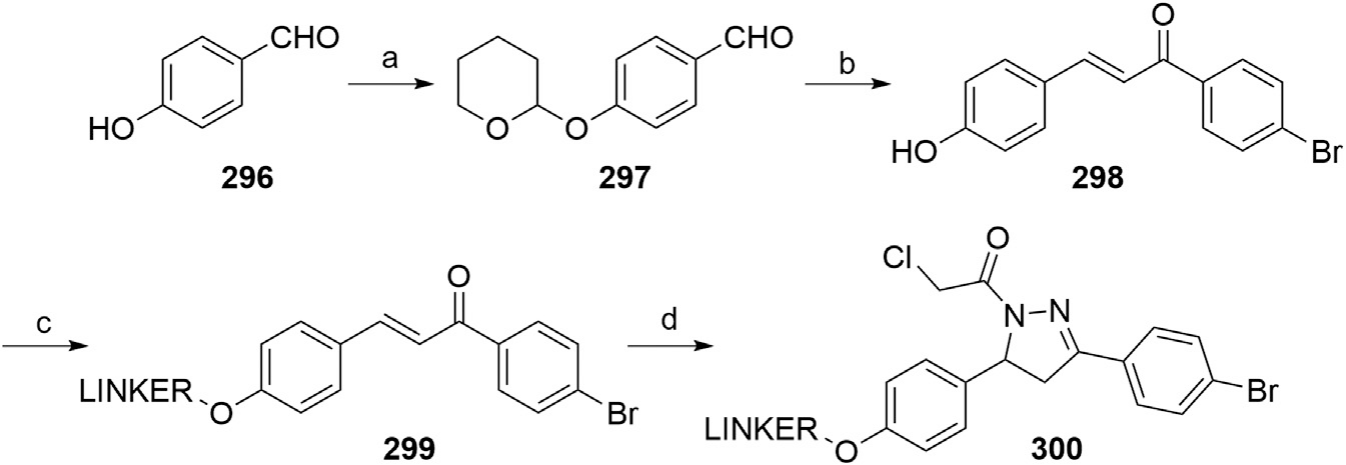
Synthesis of a fully synthetic small molecule as a covalent recruiter of RNF114 and its derivatization into PROTACs. Reagents and conditions: a) 3,4-dihydro-2*H*-pyran, pyridinium *p*-toluenesulfonate, CH_2_Cl_2_, rt, overnight, 78% yield; b) i. 4-bromoacetophenone, EtOH, 0°C, 15 min, then 10% NaOH, 0°C, 20 min, then rt, 3 h, 83% yield; ii. pyridinium *p*-toluenesulfonate, MeOH, 55°C, 3 h, 72% yield; c) linker-Br, K _2_CO_3_, DMF, 60°C, 5 h, 87% yield for linker used; d) i. Hydrazine monohydrate, EtOH, 80°C, 5 h; ii. chloroacetyl chloride, Et_3_N, CH_2_Cl_2_, 0°C, 30 min, then rt, overnight, 58% yield (Luo et al., 2021).

A small molecule, that accessed the same cysteine targeted by nimbolide was incorporated into a BRD4-targeting PROTAC (Luo et al., 2021). Starting material **296** was first protected with a tetrahydropyranyl ether to form **297** and then reacted with 4-bromoacetophenone. Following a deprotection, **298** was obtained and the OH alkylated with a bromo linker, yielding **299**. Finally, compound **300** can be achieved over 2 steps (Scheme 52) (Luo et al., 2021).

### DCAF16

DCAF16 is a member of the damage-specific DNA binding protein 1 (DDB1)-CUL4 associated factor (DCAF) protein group, which act as substrate-recognition receptors within the UPS (Liang et al., 2017). It consists of 216 amino acids and contains eight cysteine residues, four of which are clustered together between amino acids 173 and 179. By using broadly reactive, cysteine-directed electrophilic fragments, a successful covalent modification of DCAF16 was achieved to induce the degradation of BRD4 and FKBP12 (Zhang et al., 2019). The authors suggested that utilizing such electrophilic PROTACs may provide certain advantages in the field of targeted protein degradation, as DCAF16 seems to exclusively promote the degradation of nuclear proteins, sparing cytosolic ones. The covalent interaction between DCAF16 and the chimeric molecule allows for protein degradation at low fractional engagement and could prolong the degradation effect, as it should correlate with DCAF16 turnover, as opposed to the clearance of the PROTAC. This strategy may additionally have a minimal effect on DCAF16’s endogenous substrates. However, the broadly reactive fragments, utilized in DCAF16-hijacking PROTACs, still modified numerous other cellular proteins and required further optimization to enable a future for electrophilic PROTACs (Zhang et al., 2019).

#### Electrophilic DCAF16 Binder 1

**SCHEME 53:**
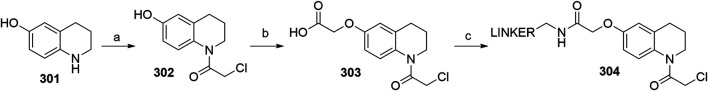
Synthesis of DCAF-hijacking degraders **304**. Reagents and conditions: a) chloroacetyl chloride, NaOH, dioxane, H_2_O, rt, 4 h; b) i. *tert*-butyl bromoacetate, Cs_2_CO_3_, DMF, rt, 3 h; ii. TFA, CH_2_Cl_2_, H_2_O, rt, 2 h, 76% yield over three steps; c) linker-NH_2_, COMU, NMP, DMF, 1 h, rt, 18–22% yield for linkers used (Zhang et al., 2019).

First of the reactive fragments used was compound **302**, which was synthesized by treating 6-hydroxy-1,2,3,4-tetrahydroquinoline (**301**) with chloroacetyl chloride. *tert*-Butyl bromoacetate was then attached to the hydroxyl group and subsequently Boc-deprotected to yield **303**, which allowed for the coupling with primary amine linkers to form conjugates **304** (Scheme 53) (Zhang et al., 2019).

#### Electrophilic DCAF16 Binder 2

**SCHEME 54:**
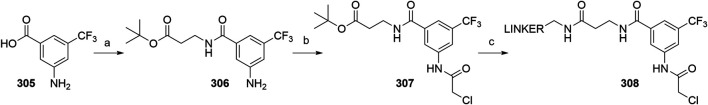
Synthesis of DCAF-hijacking degraders **308**. Reagents and conditions: a) β-alanine *tert*-butyl ester, COMU, NMP, DMF, rt, 2 h, 72% yield; b) chloroacetyl chloride, DIPEA, CH_2_Cl_2_, 0°C to rt, 2 h, 78% yield; c) i. TFA, CH_2_Cl_2_, H_2_O, rt, 2 h; ii. linker-NH_2_, COMU, NMP, DMF, rt, 1 h, 13% yield for linker used (Zhang et al., 2019).

To synthesize conjugates **308**, β-alanine *tert*-butyl ester was coupled with starting aniline derivative **305** to form **306**. The aromatic amine group underwent a reaction with chloroacetyl chloride, giving compound **307**, which was then treated with TFA to remove the Boc-protecting group and enable coupling with primary amine linkers (Scheme 54) (Zhang et al., 2019).

#### Electrophilic DCAF16 Binder 3

**SCHEME 55:**
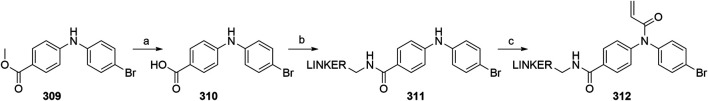
Syntheses of DCAF-hijacking degraders **312**. Reagents and conditions: a) KOH, EtOH, H_2_O, 100°C, 4 h; b) LINKER-NH_2_, COMU, NMP, DMF; rt, 2 h, 57% yield for linker used over 2 steps; c) acryloyl chloride, DIPEA, DMAP, CH_2_Cl_2_, 0°C to rt, 6 h (Note: the compound was set in the next step (POI ligand attachment) without purification) (Zhang et al., 2019).

For the third structural type of DCAF binders (compounds **312**), the synthesis started with the methyl ester **309**, which was hydrolyzed to form **310** and then coupled with primary amine linkers. Compound **311** was then treated with acryloyl chloride to incorporate a cysteine-targeting moiety into conjugates **312** (Scheme 55) (Zhang et al., 2019).

### DCAF15

DDB1-and-Cul4-associated factor 15 (DCAF15) is a substrate adaptor of the E3 ligase Rbx-Cul4-DDA1-DDB1-DCAF15. Through studying aryl sulfonamides with known anticancer activity, it has been found that indisulam stabilizes the interaction between DCAF15 and an essential splicing factor RBM39, which leads to RBM39 polyubiquitination and proteasomal degradation, thus inhibiting cell growth (Han et al., 2017; Bussiere et al., 2020).

**SCHEME 56:**
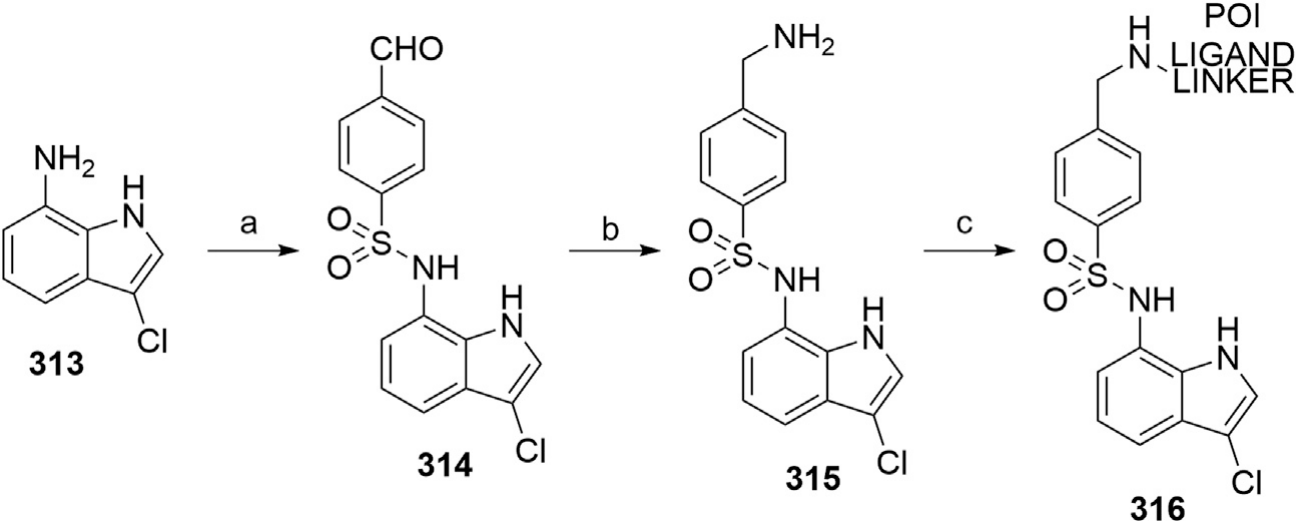
Synthesis of indisulam derivative **315** and its incorporation into degraders **316**. Reagents and conditions: a) 4-formylbenzenesulfonyl chloride, pyridine, EtOAc, rt, 3 h, 62% yield; b) sat. NH_4_OAc in EtOAc, NaBH_3_CN, 100°C, 15 min, 35% yield; c) POI ligand-linker-CO_2_H, HATU, HOAt, DIPEA, DMF, rt, 2 h, 34–37% yield for conjugates used (Zoppi et al., 2019).

Building on the known pharmacological activity of indisulam, its structure was modified to enable linker attachment and incorporation of the E3 ligase ligand into PROTACs (Zoppi et al., 2019). Starting material **313** was reacted with 4-formylbenzenesulfonyl chloride to obtain sulfonamide **314**. Reductive amination lead to amine **315**, which was then coupled with POI ligand-linker-NH_2_ conjugates to give PROTACs **316**, thus successfully expanding the E3 ligase options for targeted protein degradation (Scheme 56). However, activity of such PROTACs was only moderate (Zoppi et al., 2019).

### KEAP1

Kelch-like ECH-associated protein 1 (KEAP1) plays a key role in regulating the nuclear factor erythroid 2–related factor 2 (NRF2), which is involved in the cellular cytoprotective response to electrophiles and reactive oxygen species. Being a part of a homodimeric KEAP1/Cul3 complex that possesses E3 ubiquitin ligase activity, KEAP1 works as a substrate receptor and is responsible for selectively recognizing NRF2 and linking it to Cul3 for its ubiquitination (Davies et al., 2016; Jiang et al., 2016). Through forming reversible covalent interactions with cysteines of KEAP1, BRD4 degradation was achieved by recruiting KEAP1/Cul3 E3 ligase activity using the highly electron-deficient cyanoenone moiety-containing triterpene derivative bardoxolone (Tong et al., 2020a).

#### Bardoxolone Derivatives

**SCHEME 57:**
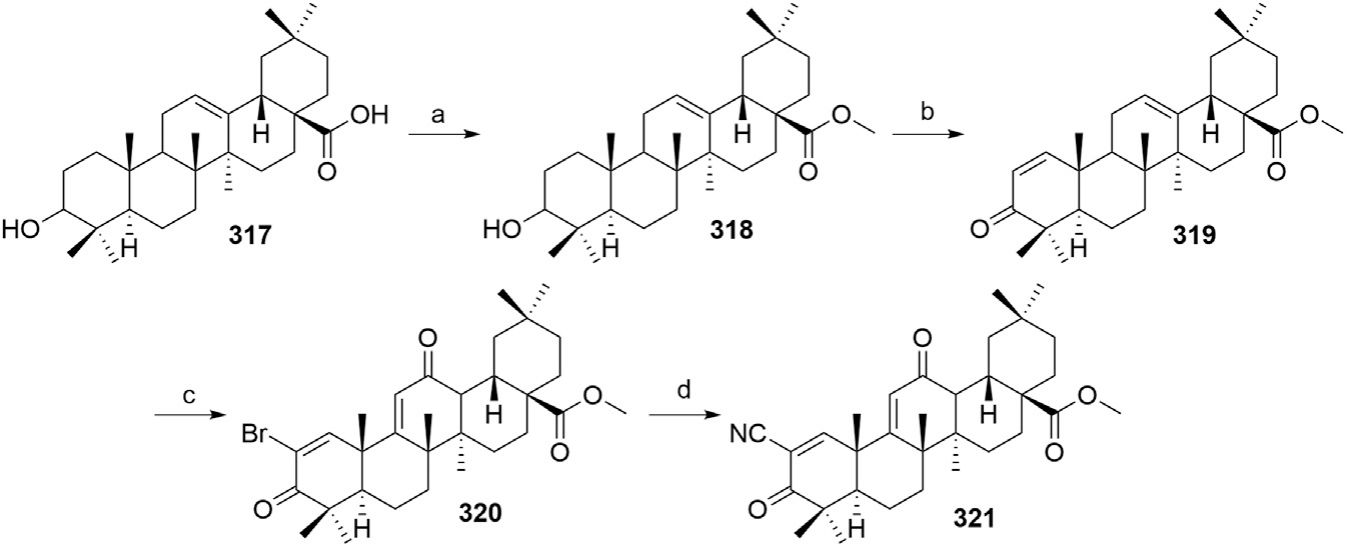
Syntheses of bardoxolone methyl (**321**) from oleanolic acid **317**. Reagents and conditions: a) K_2_CO_3_, MeI, DMF, 0°C to rt, 24 h, 99% yield; b) iodobenzoic acid, fluorobenzene, DMSO, 85°C, 24 h, 87% yield; c) i. *m*CPBA, CH_2_Cl_2_, 0°C to rt, 24 h; ii. HBr, Br_2_, acetic acid, rt to 35°C, 24 h, 82% yield; d) CuCN, KI, DMF, 120°C, 24 h, 73% yield (Fu and Gribble, 2013).

With oleanolic acid **317** as starting material, an efficient, five-step synthesis of bardoxolone methyl (**321**) was accomplished (Fu and Gribble, 2013) (Scheme 57). After forming a methyl ester **318**, the compound was oxidized to give **319**, which was then transformed into **320** over two steps. The final step consisted of substituting the bromo group with a cyano, to form bardoxolone methyl with an overall yield of 50% (Fu and Gribble, 2013). Both bardoxolone methyl and bardoxolone-CO_2_H are commercially available.

**SCHEME 58:**
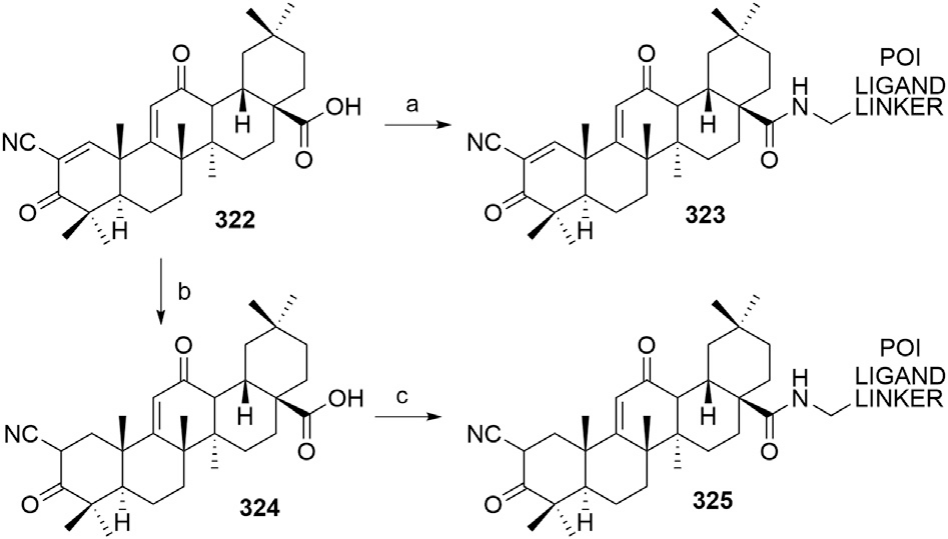
Synthesis of final KEAP1-hijacking PROTAC **323** and negative control compound **325**. Reagents and conditions: a) HATU, DIPEA, CH_2_Cl_2_, rt, 12 h, 48% yield for conjugate used; b) Pd/C, H_2_, EtOAc, rt, 25 min, 96% yield; c) HATU, DIPEA, CH_2_Cl_2_, rt, 12 h, 45% yield for conjugate used (Tong et al., 2020a).

Final PROTACs were assembled by using bardoxolone (**322**) and coupling it with POI ligand-linker-NH_2_ conjugates under standard conditions using HATU and DIPEA. Alternatively, bardoxolone was subjected to Pd/C-catalyzed hydrogenation to give compound **324**, which was incorporated into negative-control compounds **325** with the same coupling procedure (Scheme 58) (Tong et al., 2020a).

### FEM1B

FEM1B was recently discovered to play a role in regulating the cellular response to reductive stress, which can lead to various diseases, such as diabetes, cardiomyopathy, or cancer. During a depletion of reactive oxygen species (ROS), FEM1B recognizes reduced cysteines on its target Folliculin-interacting protein 1 (FNIP1) and induces its ubiquitination and subsequent degradation, which restores mitochondrial activity and redox homeostasis of the cell (Manford et al., 2020). The key cysteine residue C186 was noted to present a possible target for developing a FEM1B recruiter useful in the field of targeted protein degradation. Through screening a library of cysteine-reactive covalent ligands, compound EN106 was identified and its structure modified to be incorporated into BRD4-targeting PROTACs (Henning et al., 2021).

**SCHEME 59:**
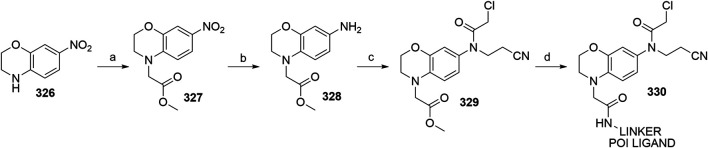
Synthesis of FEM1B-recruiting PROTACs **330**. Reagents and conditions: a) methyl bromoacetate, NaH, DMF, 0°C to rt, 94% yield; b) Fe, NH_4_Cl, EtOH, H_2_O, 80°C; c) i. acrylonitrile, alumina, reflux; ii. chloroacetyl chloride, Et_3_N, CH_2_Cl_2_, 0°C, 65% yield over 3 steps; d) i. LiOH (aq), MeOH, rt; ii. POI ligand-linker-NH_2_, HATU, DIPEA, DMF, rt, 50% yield over 2 steps for conjugate used (Henning et al., 2021).

An acetate spacer was attached to starting material **326** to give methyl ester **327**. After the nitro group was reduced, amine **328** was alkylated with acrylonitrile, followed by acylation with chloroacetyl chloride to yield **329**. After hydrolyzing the methyl ester, POI ligand-linker-NH_2_ conjugate was attached through coupling to provide PROTAC **330** (Scheme 59) (Henning et al., 2021).

### Arylhydrocarbon Receptor

The arylhydrocarbon receptor (AhR), a member of the basic helix-loop-helix (bHLH)/Per- Arnt-Sim (PAS) family of proteins, functions as a ligand-activated transcription factor. In addition to playing a role in response to xenobiotic-induced toxicity and carcinogenesis, it also acts as an adaptor protein for substrate recognition as part of CUL4^AhR^ E3 ligase complex (Ohtake et al., 2007; Luecke-Johansson et al., 2017). Studies of AhR agonists, such as 3-methylcholanthrene, α- and β-naphthoflavone (α- and β-NF) and 2,3,7,8-tetrachlorodibenzo-*p*-dioxin (TCDD), have shown that these hydrophobic compounds activate AhR, leading to the degradation of the sex steroid receptors ERα and AR, along with the self-ubiquitination of AhR (Ma and Baldwin, 2000; Ohtake et al., 2007; Busbee et al., 2013; Luecke-Johansson et al., 2017).

#### β-Naphthoflavone Derivatives

**SCHEME 60:**
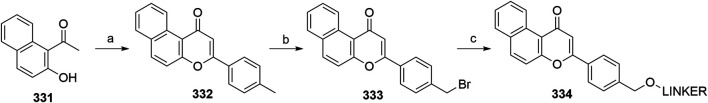
Synthesis of β-NF-derived compound **334**. Reagents and conditions: a) i. *p*-toluoyl chloride, pyridine, rt, 1 h; ii. KOH, pyridine, 60°C, 30 min; iii. H_2_SO_4_, AcOH, 110°C, 6 h, 69% yield; b) NBS, AIBN, CCl_4_, 80°C, 12 h, 82% yield; c) OH-linker, NaH, THF, rt, 24 h, 43% yield for linker used (Ohoka et al., 2019).

Building on established AhR agonists, β-NF and 2-(1′H-indole-3′-carbonyl)-thiazole-4-carboxylic acid methyl ester (ITE), chimeric molecules have been developed that induce the degradation of cellular retinoic acid binding proteins (CRABPs) and BRD by hijacking AhR for its E3 ligase activity. Naphthoflavone derivative **332** was synthesized from 2-hydroxy-1′-acetonaphthone (**331**) over three steps. The product was then subjected to radical bromination using NBS and AIBN to yield **333**, which was then alkylated with a hydroxy-containing linker to give **334** (Scheme 60) (Ohoka et al., 2019).

#### ITE Derivatives

**SCHEME 61:**
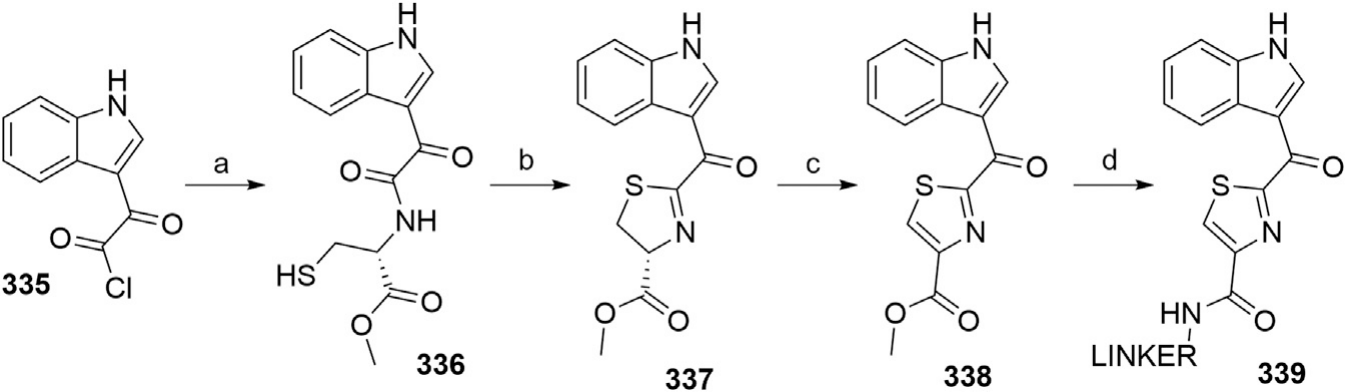
Synthesis of ITE (**338**) and its derivatization for use in degraders. Reagents and conditions: a) l-cysteine methyl ester, Et_3_N, benzene, 0°C to rt, 20 h, then reflux, 2.5 h, 55% yield; b) TiCl_4_, CH_2_Cl_2_, reflux, 5 h, then rt, 16 h, 25% yield; c) MnO_2_, CH_2_Cl_2_, rt, 3 h, 88% yield (DeLuca et al., 2003); d) i. 4 M KOH (aq), THF, rt, 16 h; ii. linker-NH_2_, HATU, DIPEA, DMF, rt, 16 h, 57% yield over 2 steps for linker used (Ohoka et al., 2019).

To synthesise endogenous AhR ligand ITE, l-cysteine methyl ester was acylated with starting material **335** to obtain glyoxylamide **336**. The following cyclization was performed using TiCl_4_ in CH_2_Cl_2_, forming thiazoline ester **337**. Oxidation with MnO_2_ yielded ITE (**338**) (DeLuca et al., 2003), the methyl ester of which was then hydrolyzed and available for coupling with amine linkers to obtain compound **339**, available for incorporation into chimeric degraders (Scheme 61) (Ohoka et al., 2019).

## Conclusion

**FIGURE 9:**
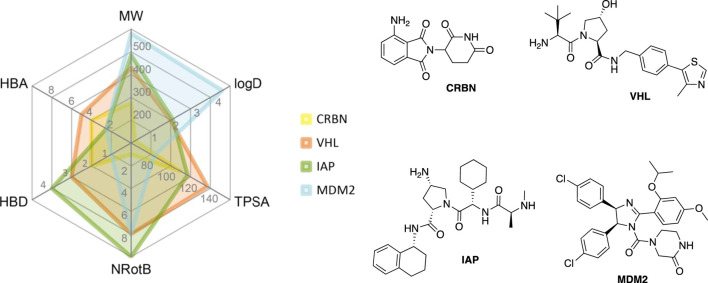
Radar plot incorporating the molecular descriptors of representative CRBN, VHL, IAP, and MDM2 ligands. Values were calculated with SwissADME (Daina et al., 2017).

**FIGURE 10:**
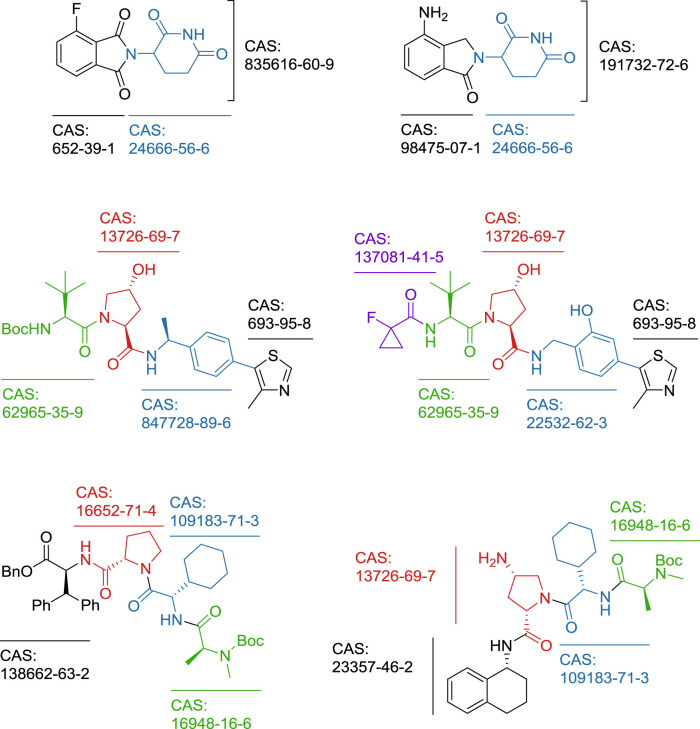
CAS numbers of building blocks for commonly utilized CRBN, VHL, and IAP ligands.

Principles of PROTAC design and tackling the challenges that accompany the field were explored extensively (Maple et al., 2019). To reiterate those findings, we would like to briefly touch on optimization in the early stages of planning chimeric molecules in a way that increases the likelihood of obtaining potent, cell-permeable degraders. In Figure 9, a representative ligand for each of the four most commonly used E3 ligases is presented along with its molecular descriptors. This radar plot can serve as a quick navigational tool to evaluate how the chosen E3 ligase ligand might contribute to the physicochemical properties of final degraders and aid in ligand selection in order to balance out the size, lipophilicity, and related characteristics that affect the success rate of PROTACs. Additionally, a selection of commercially available building blocks for the most widely applied CRBN, VHL, and IAP ligands are collected in Figure 10 and can be used as a quick aid for those starting with E3 ligase ligand synthesis.

This review gives an extensive overview of successful synthetic routes towards functionalized E3 ligase ligands. This enables the reader to better assess which reaction conditions are suitable and which yields can be achieved. Most of the starting materials are either commercially available or can be produced by simple synthesis techniques. Due to the scope of our research, this review may give new impulses in the synthesis laboratories to try out new linker connections or to test novel reactions under proven conditions. Ultimately, it is not only the accessibility and capital efficiency that determine the success of a PROTAC program, but also aspects such as rigidity, hydrolytic and metabolic stability, solubility and cell permeability of the chimeric molecules. This work represents a unique compendium to re-evaluate the many facets involved in the synthesis of such complex molecules.

## Glossary

AhR: arylhydrocarbon receptor
AIBN: azobisisobutyronitrile
AR: androgen receptor
bHLH: basic helix-loop-helix
BIPHEP: bis(diphenylphosphino)biphenyl
BRD: bromodomain-containing protein
CDI: carbonyldiimidazole
BTK: Bruton’s tyrosine kinase
cIAP: cellular inhibitor of apoptosis
CLIPTACs: in-cell click-formed proteolysis targeting chimeras
COMU: (1-Cyano-2-ethoxy-2-oxoethylidenaminooxy)dimethylamino-morpholino-carbenium-hexafluorophosphate
CPME: cyclopentylmethylether
CRABPs: cellular retinoic acid binding proteins
CRBN: cereblon
CRL: cullin/RING E3 ubiquitin ligase
DBAD: di-*tert*-butyl azodicarboxylate
DBU: diazabicycloundecene
DCAF: DDB1-and-CUL4-associated factor
DCC: *N*,*N*′-Dicyclohexylcarbodiimide
DDB: damage-specific DNA binding protein
DEACM: diethylamino coumarin
DIAD: diisopropyl azodicarboxylate
DIPEA: *N*,*N*-diisopropylethylamine
DMA: *N*,*N*-dimethylacetamide
DMAP: 4-(*N*,*N*-dimethylamino)pyridine
DMNB: 4,5-dimethoxy-2-nitro-benzyl
DTBAD: di-*tert*-butyl azodicarboxylate
E1: ubiquitin-activating enzyme
E2: ubiquitin-conjugating enzyme
E3: ubiquitin ligase
EDC: 1-ethyl-3-(3-dimethylaminopropyl)carbodiimide
*Ent*: enantiomeric
ER: Estrogen receptor
FKBP12: peptidyl-prolyl cis-trans isomerase
Fmoc: fluorenylmethyloxycarbonyl
FNIP1: lolliculin-interacting protein 1
HATU: 1-[bis(dimethylamino)methylene]-1*H*-1,2,3-triazolo[4,5-*b*]pyridinium 3-oxid hexafluorophosphate
HCTU: 2-(6-chloro-1*H*-benzotriazol-1-yl)-1,1,3,3-tetramethylaminium-hexafluorophosphate
HECT: homologous to E6AP C-terminus
HIF: hypoxia-inducible factor
HMBC: heteronuclear multiple bond correlation
HOAt: 1-hydroxy-7-azabenzotriazole
HOBt: 1-hydroxybenzotriazole
IAP: inhibitor of apoptosis proteins
IKZF: transcription factors Ikaros (IKZF1) and Aiolos (IKZF3)
IMiDs: immunomodulatory imide drugs
ITE: 2-(1′*H*-indole-3′-carbonyl)-thiazole-4-carboxylic acid methyl ester
KEAP1: Kelch-like ECH-associated protein 1
*m*CPBA: *meta*-chloroperoxybenzoic acid
MDM2: mouse double minute 2 homologue
ML-IAP: melanoma IAP
MTBE: methyl *tert*-butyl ether
MW: microwave
NF: naphthoflavone
NMP: N-methylpyrrolidone
NPOM: 6-nitropiperonyloxymethyl
NRF2: nuclear factor erythroid 2–related factor 2
NS3: nonstructural protein 3, hepatitis C virus protein
PARP1: poly(ADP-ribose)-polymerase 1;
PAS: Per-Arnt-Sim
Pd/C: palladium on activated carbon
Phosferrox: phosphinoferrocenyloxazoline
PHOTACs: photochemically targeting chimeras
POI: protein of interest
PROTACs: proteolysis targeting chimeras
PyBOP: benzotriazol-1-yl-oxytripyrrolidinophosphonium hexafluorophosphate
RBR: RING-between-RING
Rbx-1: RING-box protein 1
RING: really interesting new gene
RNF114: RING-type zinc-finger protein 114
ROS: reactive oxygen species
rt: room temperature
SCF^β-TRCP^: Skp1–Cullin–F box complex
SFC: supercritical fluid chromatography
Smac/DIABLO: second mitochondria-derived activator of caspase/direct inhibitor of apoptosis-binding protein with low pI
SNIPERs: specific and nongenetic IAP-dependent protein erasers
TBAF: tetra-*N*-butylammonium fluoride
TBAI: tetra-*N*-butylammonium iodide
TBDPSCl: *tert*-butyl(chloro)diphenylsilane
TBSCl: *tert*-butyl(chloro)dimethylsilane
TBTU: 2-(1*H*-benzotriazole-1-yl)-1,1,3,3-tetramethylaminium tetrafluoroborate
TCDD: 2,3,7,8-tetrachlorodibenzo-p-dioxin
TEMPO: 2,2,6,6-tetramethylpiperidine-1-oxyl
Teoc-OSu: 1-[2-(trimethylsilyl)ethoxycarbonyloxy]pyrrolidin-2,5-dione
Tf/triflic: trifluoromethanesulfonyl
TIPS: triisopropylsilyl
Tle: 2-amino-3,3-dimethylbutyric acid
TPPO: triphenylphosphine oxide
TrkC: tropomyosin receptor kinase C
Trt/trityl: triphenylmethyl
UIM: ubiquitin-interacting motif
UPS: ubiquitin proteasome system
VHL: von-Hippel–Lindau
XIAP: chromosome-linked IAP
